# Integrating genome-wide polygenic risk scores and non-genetic risk to predict colorectal cancer diagnosis using UK Biobank data: population based cohort study 

**DOI:** 10.1136/bmj-2022-071707

**Published:** 2022-11-09

**Authors:** Sarah E W Briggs, Philip Law, James E East, Sarah Wordsworth, Malcolm Dunlop, Richard Houlston, Julia Hippisley-Cox, Ian Tomlinson

**Affiliations:** 1 Nuffield Department of Medicine, University of Oxford, Oxford, UK; 2 Division of Genetics and Epidemiology, Institute of Cancer Research, London, UK; 3 Translational Gastroenterology Unit, Nuffield Department of Medicine, University of Oxford, Oxford, UK; 4 NIHR Oxford Biomedical Research Centre, University of Oxford, Oxford, UK; 5 Health Economics Research Centre, Nuffield Department of Population Health, University of Oxford, Oxford, UK; 6 Colon Cancer Genetics Group, Medical Research Council Human Genetics Unit, Institute of Genetics and Cancer, University of Edinburgh, Edinburgh, UK; 7 Division of Genetics and Epidemiology, Institute of Cancer Research, London, UK; 8 Nuffield Department of Primary Care Health Sciences, University of Oxford, Oxford, UK; 9 Cancer Research, Institute of Genetics and Cancer, University of Edinburgh, Edinburgh, UK

## Abstract

**Objective:**

To evaluate the benefit of combining polygenic risk scores with the QCancer-10 (colorectal cancer) prediction model for non-genetic risk to identify people at highest risk of colorectal cancer.

**Design:**

Population based cohort study.

**Setting:**

Data from the UK Biobank study, collected between March 2006 and July 2010.

**Participants:**

434 587 individuals with complete data for genetics and QCancer-10 predictions were included in the QCancer-10 plus polygenic risk score modelling and validation cohorts.

**Main outcome measures:**

Prediction of colorectal cancer diagnosis by genetic, non-genetic, and combined risk models. Using data from UK Biobank, six different polygenic risk scores for colorectal cancer were developed using LDpred2 polygenic risk score software, clumping, and thresholding approaches, and a model based on genome-wide significant polymorphisms. The top performing genome-wide polygenic risk score and the score containing genome-wide significant polymorphisms were combined with QCancer-10 and performance was compared with QCancer-10 alone. Case-control (logistic regression) and time-to-event (Cox proportional hazards) analyses were used to evaluate risk model performance in men and women.

**Results:**

Polygenic risk scores derived using the LDpred2 program performed best, with an odds ratio per standard deviation of 1.584 (95% confidence interval 1.536 to 1.633), and top age and sex adjusted C statistic of 0.733 (95% confidence interval 0.710 to 0.753) in logistic regression models in the validation cohort. Integrated QCancer-10 plus polygenic risk score models out-performed QCancer-10 alone. In men, the integrated LDpred2 model produced a C statistic of 0.730 (0.720 to 0.741) and explained variation of 28.2% (26.3 to 30.1), compared with 0.693 (0.682 to 0.704) and 21.0% (18.9 to 23.1) for QCancer-10 alone. In women, the C statistic for the integrated LDpred2 model was 0.687 (0.673 to 0.702) and explained variation was 21.0% (18.7 to 23.7), compared with 0.645 (0.631 to 0.659) and 12.4% (10.3 to 14.6) for QCancer-10 alone. In the top 20% of individuals at highest absolute risk, the sensitivity and specificity of the integrated LDpred2 models for predicting colorectal cancer diagnosis was 47.8% and 80.3% respectively in men, and 42.7% and 80.1% respectively in women, with increases in absolute risk in the top 5% of risk in men of 3.47-fold and in women of 2.77-fold compared with the median. Illustrative decision curve analysis indicated a small incremental improvement in net benefit with QCancer-10 plus polygenic risk score models compared with QCancer-10 alone.

**Conclusions:**

Integrating polygenic risk scores with QCancer-10 modestly improves risk prediction over use of QCancer-10 alone. Given that QCancer-10 data can be obtained relatively easily from health records, use of polygenic risk score in risk stratified population screening for colorectal cancer currently has no clear justification. The added benefit, cost effectiveness, and acceptability of polygenic risk scores should be carefully evaluated in a real life screening setting before implementation in the general population.

## Introduction

Colorectal cancer is the fourth most common cancer in the UK, with increasing incidence in younger ages and countries with historically lower rates.^1^ Population screening is effective in reducing colorectal cancer incidence and mortality, through detection and removal of premalignant adenomas and earlier detection of cancers. Screening modalities vary internationally. Although colonoscopy is the gold standard, this procedure is expensive, invasive, and time consuming. Many countries have adopted a staged process, with initial faecal immunochemical testing, followed by colonoscopy for people who test positive. Approaches stratified by risk which direct screening resources to people at highest risk have the potential to improve screening detection rates, reduce investigative burden of people at lower risk, and potentially improve cost effectiveness.^2^ Improved understanding of cancer risk could also improve informed consent and shared decision making around screening participation.

Both genetic and non-genetic factors contribute to an individual’s risk of colorectal cancer. Some non-genetic factors are modifiable. The top performing risk model by non-genetic factors in external validation is QCancer-10 (colorectal cancer),^3^ ^4^ which has been recommended as a tool to guide shared decision making around colorectal cancer screening.^5^ QCancer-10 is a 15 year colorectal cancer prediction model, developed using the QResearch linked primary care database of almost 5 million individuals aged 25-84 years, registered at QResearch practices across England between 1998 and 2013.^4^ The tool is based on age, ethnic group, family history, alcohol and smoking status, a small number of medical conditions, and for men (value was not sufficient for these to be selected for inclusion in the model in women), Townsend deprivation score and body mass index. As the predictors are derived from electronic health records, this tool could be embedded at point of care and linked with screening records to facilitate risk stratification within the bowel screening programme.

Genetic variants known to predispose to colorectal cancer are mostly single nucleotide polymorphisms identified as significantly associated with riskin genome-wide association studies (GWAS). Genetic risk can be summarised in a polygenic risk score (PRS). Most PRSs have used a limited set (typically tens) of significantly associated single nucleotide polymorphisms, with genotypes weighted by predicted effect sizes.^6^ More recently, genome-wide PRSs have incorporated many more single nucleotide polymorphisms than those reaching GWAS significance, on the basis that many true risk single nucleotide polymorphisms remain unidentified. These models have generally produced better performance than GWAS significant models, but evaluation in colorectal cancer has been limited.^7^ ^8^ A further issue is that several previous evaluations of colorectal cancer PRS in the UK Biobank study are based on summary statistics derived from a GWAS meta-analyses that included findings from UK Biobank.^8^ ^9^ This overlap results in overfitting of models and overestimation biases (known as optimism) in performance estimates.^10^

Integrated models for colorectal cancer, which have combined GWAS significant PRS with non-genetic risk factors, generally do better than non-genetic models or PRS alone.^6^ ^9^ We hypothesised that combining PRS with QCancer-10 will provide enhanced risk prediction and that genome-wide PRSs will give the greatest benefit. We used the UK Biobank study to develop and compare PRS using several approaches in a white British cohort from England and Wales, minimising overfitting and optimism by using summary GWAS data that did not overlap with the UK Biobank study dataset. We validated our findings in geographical (Scotland) and minority ethnic validation cohorts from within UK Biobank. We then derived integrated QCancer-10+PRS risk models, using the top performing, genome-wide PRS and the GWAS significant PRS, which we internally validated and compared with QCancer-10.

## Methods

### Study design

We conducted a development and validation study of PRS and integrated PRS-epidemiological models to predict risk of colorectal cancer in a set of UK individuals of bowel cancer screening age. We followed the PRS-Reporting Standards (PRS-RS) and Transparent reporting of a multivariable prediction model for individual prognosis or diagnosis (TRIPOD) guidelines for PRS and prediction modelling.^11^ ^12^

We used the UK Biobank study to derive and validate our risk models, under application number 8508.^13^ In brief, just over 500 000 participants aged 40-69 (5.5% of invitees) were recruited to UK Biobank from the general population across the UK between March 2006 and July 2010.^14^ Baseline demographics; medical, lifestyle, and physical data; and blood samples were collected at recruitment. Follow-up through linked hospital, general practice, and registry data is ongoing. A detailed description of genetic resources including quality control measures can be found elsewhere^13^ (supplementary methods). Participants were genotyped for genome-wide tag single nucleotide polymorphism panels (49 950 individuals on the Applied Biosystems UK (Waltham, MA, USA) BiLEVE Axiom Array and the remainder (about 450 000 individuals) on the Applied Biosystems UK Biobank Axiom Array, which share over 95% content). Following quality control, genotype phasing was carried out using SHAPEIT3 with 1000 Genomes phase 3 as a reference panel, followed by imputation using IMPUTE4 with the Haplotype Reference Consortium dataset as the main reference panel, and secondarily with merged UK10K and 1000 Genomes phase 3 reference panels, and the datasets combined. Annotation of single nucleotide polymorphisms was based on the Genome Reference Consortium Human Build 37 assembly of the human genome.

### Outcomes

The primary outcome in all models was colorectal cancer diagnosis, identified through self-report at UK Biobank study enrolment visit and International Classification of Diseases-9 (153, 154.0, 154.1) and International Classification of Diseases-10 (C18-C20) codes in linked cancer and death registries and hospital data. For PRS development and evaluation in logistic regression models, we included incident and prevalent cases, with the remaining cohort used as controls. For time-to-event analysis by use of Cox proportional hazards models, we excluded prevalent cases with a diagnosis of colorectal cancer before cohort entry. Follow-up began at the date of enrolment and was censored at the earliest of date of incident colorectal cancer, loss to follow-up, death, or end of available registry follow-up (31 October 2015 for Scottish participants; 13 March 2016 for all other participants).

We calculated age specific and directly standardised colorectal cancer incidence rates in UK Biobank overall and for the Integrated Modelling Cohort used to derive integrated QCancer-10+PRS risk models, and compared these with Office for National Statistics 2013 cancer registry data for England (chosen as the approximate mid-point of available UK Biobank follow-up).^15^ Age specific rates were calculated in five year age bands between 40 and 80 years as the number of first incident colorectal cancers over the number of person years at risk. Age standardised incidence rates were calculated using the 2013 European Standard Population aged 40-80 years.^16^ Rates are presented per 100 000 person years at risk (supplementary methods).

### Polygenic risk scores

We did a meta-analysis of summary data from 14 colorectal cancer GWAS cohorts (which did not include UK Biobank, hereafter termed the base GWAS data), to provide association effect sizes of single nucleotide polymorphisms (supplementary methods^17^). 26 397 cases and 41 481 controls were available, all of European ancestry based on principal components analysis. We performed the meta-analysis using the meta package (version 1.7),^18^ including single nucleotide polymorphisms imputed with an imputation quality (INFO) score of more than 0.8 from each dataset, using the inverse variance method for fixed effects.

We evaluated six PRS models from three broad approaches to PRS development (supplementary methods). Firstly, we used a standard PRS (hereafter GWAS significant), which comprised a manually curated list of 50 sentinel single nucleotide polymorphisms shown in GWAS meta-analyses of European data,^17^ ^19^ to be independently and reproducibly associated with colorectal cancer risk at P<5×10^-8^ in our meta-analysis. This PRS was constructed as a log-additive sum of single nucleotide polymorphism dosages weighted by their betas. Betas were adjusted for winner’s curse using FDR Inverse Quantile Transformation** (**FIQT) correction.^20^ Secondly, we evaluated genome-wide clumping and thresholding (C+T) methods using standard and stacked (SCT) approaches.^21^ Thirdly, we used LDpred2,^22^ which takes a bayesian approach to single nucleotide polymorphism selection, accounting for linkage disequilibrium between the single nucleotide polymorphisms. We used three different LDpred2 options: an infinitesimal model (LDpred2-Inf), a non-sparse grid model (LDpred2-grid), and a sparse grid model (LDpred2-grid-sp).

We show the quality control measures per person for the genetic data and sample exclusions for each modelling cohort. We used imputed dosage data from UK Biobank, and restricted single nucleotide polymorphisms to those included in the HapMap3 reference dataset and those present in the base GWAS data. After quality control, 1 104 409 single nucleotide polymorphisms were available for PRS development (supplementary methods, supplementary fig S1).

PRSs were developed in the derivation dataset, which included participants of white British ancestry (identified through self-reported ethnic group and genetic information)^13^ from England and Wales (supplementary methods). The derivation dataset was divided into a training and a test cohort. Optimal PRS tuning parameters for genome-wide approaches were selected in the training cohort (supplementary methods, supplementary fig S2). For each optimal PRS, we assessed association with colorectal cancer risk in logistic regression and Cox proportional hazards risk models in the test cohort, adjusting for age, sex, genotyping array, and the first four principal components from UK Biobank. We tested for interactions between age and PRS. Case prevalence of colorectal cancer was 1.5% in both cohorts. We compared performance with a reference model containing age, sex, genotyping array, and four principal components, without the PRS. We also evaluated performance without age and sex in the model.

We reported the distribution of standardised PRS and adjusted odds ratios and hazard ratios per standard deviation (supplementary methods). We used the C statistic (Harrell’s C index for Cox proportional hazards models) and Somers’ D_xy_ statistic to assess discrimination, in addition to Royston’s D statistic and separation of Kaplan-Meier curves across four risk groups (cut at 16th, 50th, and 84th centiles, approximating to the mean and 1 standard deviation)^23^ for Cox proportional hazards models. Nagelkerke’s R^2^ was used in logistic regression models and Royston and Sauerbrei's R^2^_D_ in Cox proportional hazards models to assess variance explained, and R^2 ^attributable to the PRS was calculated by R^2^ (full model) minus R^2^ (reference model). These measures were evaluated over the follow-up time of the cohort for Cox proportional hazards models. Scaled Brier scores (derived from the Brier score scaled to the maximum possible score for a given dataset, where a higher percentage score indicates better performance^24^) were used to assess overall model performance, calculated at eight years of follow-up for Cox proportional hazards models. Each model was internally validated. Confidence intervals and internal validation used 500 bootstrap samples.

Before external validation, models were adjusted for optimism. The optimism adjusted calibration slope was used as a global shrinkage factor to adjust the regression coefficients, and the intercept or baseline survival function was re-estimated (by refitting the model with the adjusted linear predictor as an offset).^25^ Adjusted PRS models were then applied to a geographical validation cohort, comprising Scottish participants with European ancestry, and a minority ethnic validation cohort (from any UK region). The null hypothesis of no difference in performance statistics between models was tested using paired t tests. In addition to the performance metrics described previously, calibration was assessed through the calibration slope and visual assessment of calibration plots, with calibration-in-the-large for logistic regression models. For the prespecified subgroups, we analysed the geographical validation cohort by sex, by age, and in people with a first degree family history of colorectal cancer (supplementary methods). We evaluated potential improvements in calibration in validation datasets obtained through recalibration-in-the-large (in which the intercept or baseline survival function is re-estimated in the new dataset).

### Development of QCancer-10+PRS combined models

Coding of QCancer-10 predictors in UK Biobank was matched as closely as possible to the original model.^4^ Ethnic group, previous medical history, alcohol use and smoking status, and family history of colorectal cancer were all obtained from self-reported data in baseline touch screen responses and verbal interviews at UK Biobank assessment centres. Mapping of QCancer-10 predictors to UK Biobank data and coding of predictors is described in supplementary methods and supplementary tables S1 and S2.

The integrated modelling cohort used for QCancer-10 validation and development of integrated models comprised all individuals with imputed genetic data passing quality control, excluding the individuals in the PRS training cohort (supplementary methods), and with complete QCancer-10 predictor data. Since missingness was less than 5% for all predictors (supplementary table S3), we used complete case analysis. Sample size adequacy for integrated model development was calculated following Riley et al^26^ (supplementary methods).

We validated QCancer-10 performance in UK Biobank, and recalibrated the model for the UK Biobank dataset through recalibration-in-the-large. Full QCancer-10 model specification is available at https://www.qcancer.org/15yr/colorectal/. We then developed integrated models including the risk score from QCancer-10 plus either the top performing genome-wide PRS (based on the maximum C statistic and R^2^ in external validation) or the GWAS significant PRS, with PRS adjusted for genotyping array and the first four principal components from UK Biobank, using Cox models, developed in men and women separately. Inspection of Schoenfeld residuals showed that the proportional hazard assumption held. We evaluated the use of multiple fractional polynomials to model the predictors, ultimately using a fractional polynomial term to model the genome-wide PRS in the model for women. We assessed possible interactions between the predictors by visual inspection of plots of marginal effects of the QCancer-10 risk score across PRS values and examining the prognostic strength and significance of interaction terms based on Wald χ^2^statistics.

We used the same metrics to assess the original QCancer-10 model and QCancer-10+PRS model performance as described for Cox PRS models, with paired t tests to compare models as previously described. Confidence intervals and internal validation used 500 bootstrap samples. We undertook a sensitivity analysis excluding people diagnosed within two years of recruitment to evaluate possible reverse causality. Prespecified subgroup analyses for QCancer-10 and QCancer-10+PRS included people with a first degree family history of colorectal cancer, analysis by self-reported ethnic group (minority ethnic participants compared with white participants), and calibration by age. As we observed some miscalibration of PRS models in some age groups, we also evaluated performance of QCancer-10 and QCancer-10+PRS models across three age groups (<50 years, 50-59 years, ≥60 years).

Model sensitivities, specificities, detection rates, and false positive rates were calculated at centile thresholds for absolute risk and relative risk. Relative risks were calculated relative to an individual of the same age and sex, mean PRS (by sex), mean principal components, body mass index of 25, white ethnic group, mean Townsend score, and no other colorectal cancer risk factors.

### Decision curve analysis

A full evaluation of the clinical usefulness of PRS and the integrated risk score in a population screening setting is complex because the assessment must take into account participation rates, screening frequency, method used (eg, faecal immunochemical testing or primary colonoscopy), criteria used to select participants for colonoscopy (eg, by age or faecal immunochemical testing result), and success at preventing colorectal cancer by removal of premalignant lesions. We could, however, consider a simplified situation, in which we assume a single colonoscopy at the start of an eight year follow-up period would detect all colorectal cancers and relevant premalignant lesions, with participant benefit in those cases. We then captured the complex benefits arising from this screening in a simple but standard measure of net benefit obtained using QCancer-10 and QCancer-10+PRS models to select individuals for screening colonoscopy: NB=(true positives÷N)–(false positives÷N)(P_t_ ÷ (1−P_t_)), where N is the number of individuals in the integrated modelling cohort and P_t_ is the probability (or risk) threshold (ie, at P_t_ = 1%, we are willing to perform colonoscopy for 100 individuals to detect one cancer).^27^

We plotted net benefit and unnecessary interventions avoided (which represents true negatives) across relevant risk thresholds over eight years of follow-up in decision curves. We reported values for net benefit, unnecessary interventions avoided, and test trade-off at a range of prespecified risk thresholds (0.5%, 1%, 1.5%, and 2%; supplementary methods).^27^ ^28^ For decision curve and subgroup analyses, QCancer-10+PRS models were first adjusted for optimism, and recalibrated QCancer-10 models were used. We used R (version 3.6.2) for statistical analysis.^29^

### Patient and public involvement

The concept and design of this study was informed by discussions with individuals at Bowel Cancer UK, including patient representatives. Several members of the public reviewed the paper and provided feedback.

## Results

**Fig 1:**
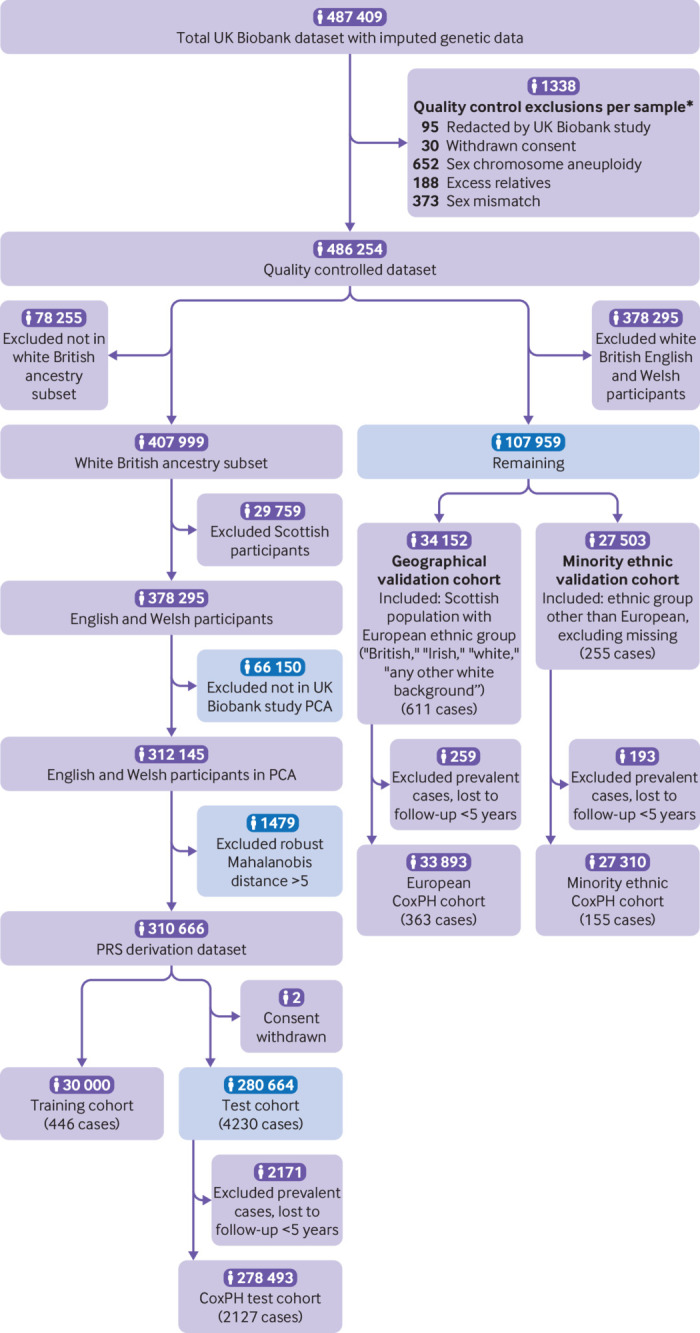
UK Biobank participant flow diagram of quality control and derivation of PRS modelling cohorts. Blue boxes indicate the data that were used in the integrated modelling cohorts, shown in figure 2. CoxPH=Cox proportional hazards modelling; PCA=principal components analysis; PRS=polygenic risk score.*More than one exclusion might apply per person

**Fig 2:**
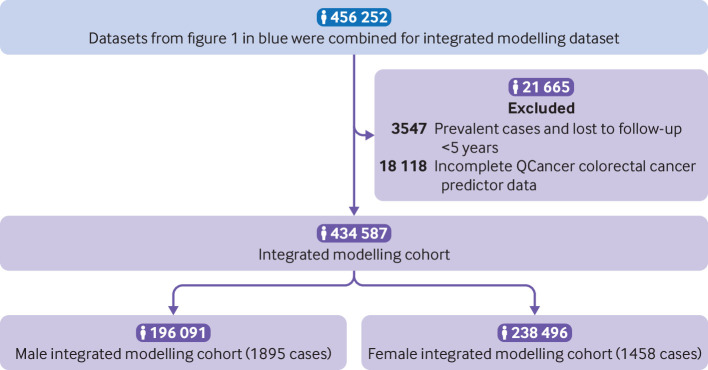
UK Biobank participant flow diagram for the integrated modelling cohorts.

We report the study profile of included participants in the quality controlled dataset for PRS development and validation (fig 1), comprising the PRS training cohort (n=30 000, 446 cases), the test cohort (n=280 664; 4230 cases), the geographical validation cohort (n=34 152) and the minority ethnic cohort (n=27 503), and we also report the integrated modelling cohort (n=434 587) (fig 2). The available sample size and incident cases available for integrated model development for women (n=238 496, 1458 cases) fell below predicted sampled size requirements (n=253 780, 1569 cases); sample size for men was adequate (supplementary methods). Demographics for the integrated modelling cohort, derived from the UK Biobank study, are shown in table 1. Supplementary table S4 gives these values, including numbers not reported, for the whole UK Biobank study cohort; characteristics of each PRS cohort are shown in supplementary table S5. From linked cancer registry data in the whole UK Biobank study cohort versus data from the Office for National Statistics, age standardised colorectal cancer incidence was lower: 108.3 (*v* 127.8) cases in men per 100 000 person years at risk and 73.9 (*v* 80.7) cases in women.^15^ Incidence per 100 000 person years of follow-up in the integrated modelling cohort, with cases of colorectal cancers identified through all linked data, was 118.0 in men and 79.3 in women. Age specific incidence rates in UK Biobank (supplementary fig S3) closely followed those data from the Office for National Statistics until the age of 70 years, after which UK Biobank rates were lower.

**Table 1:** Demographic data and medical conditions included in QCancer-10 models, in the male and female integrated modelling cohorts, and in cases of colorectal cancer. Values are numbers (%) unless otherwise indicated

	Male cohort (n=196 091)	Cases in men (n=1895)	Female cohort (n=238 946)	Cases in women (n=1458)
Follow-up (years), median (IQR)	7.1 (1.3)	3.7 (3.5)	7.1 (1.3)	3.8 (3.4)
Age (years), median (IQR)	58 (13)	63 (8)	57 (13)	61 (10)
Geographical region:				
East Midlands	13 254 (6.8)	127 (6.7)	16 175 (6.8)	88 (6.0)
London	25 843 (13.2)	203 (10.7)	33 080 (13.9)	150 (10.3)
North East	22 789 (11.6)	221 (11.7)	27 688 (11.6)	174 (11.9)
North West	30 259 (15.4)	325 (17.2)	35 278 (14.8)	203 (13.9)
Scotland	14 690 (7.5)	173 (9.1)	18 729 (7.9)	150 (10.3)
South East	16 812 (8.6)	165 (8.7)	21 367 (9.0)	179 (12.3)
South West	16 467 (8.4)	150 (7.9)	21 086 (8.8)	136 (9.3)
Wales	8150 (4.2)	90 (4.7)	9942 (4.2)	63 (4.3)
West Midlands	18 530 (9.4)	154 (8.1)	19 783 (8.3)	128 (8.8)
Yorkshire and Humber	29 297 (14.9)	287 (15.1)	35 368 (14.8)	187 (12.8)
Ethnic group:				
White*	185 016 (94.4)	1836 (96.9)	225 078 (94.4)	1396 (95.7)
Indian	2510 (1.3)	11 (0.6)	2601 (1.1)	12 (0.8)
Pakistani	903 (0.5)	1 (0.1)	616 (0.3)	4 (0.3)
Bangladeshi	132 (0.1)	0 (0.0)	61 (0.0)	0 (0.0)
Other Asian	841 (0.4)	2 (0.1)	748 (0.3)	2 (0.1)
Black African	1397 (0.7)	5 (0.3)	1412 (0.6)	6 (0.4)
Caribbean	1363 (0.7)	8 (0.4)	2498 (1.0)	10 (0.7)
Chinese	516 (0.3)	2 (0.1)	865 (0.4)	5 (0.3)
Other ethnic group	2616 (1.3)	18 (0.9)	3980 (1.7)	20 (1.4)
Not recorded*	797 (0.4)	12 (0.6)	637 (0.3)	3 (0.2)
Townsend deprivation index, median (IQR)	–2.18 (4.19)	–2.33 (4.19)	–2.17 (4.09)†	–2.38 (3.96)†
Body mass index, median (IQR)	27.28 (5.04)	27.92 (5.11)	26.08 (6.23) †	26.42 (6.00)†
Smoking status:				
Non-smoker	97 088 (49.5)	739 (39.0)	142 569 (59.8)	820 (56.2)
Ex-smoker	75 100 (38.3)	935 (49.3)	74 934 (31.4)	525 (36.0)
Light smoker	9361 (4.8)	84 (4.4)	8885 (3.7)	43 (2.9)
Moderate smoker	5816 (3.0)	43 (2.3)	7235 (3.0)	43 (2.9)
Heavy smoker	8726 (4.4)	94 (5.0)	4873 (2.0)	27 (1.9)
Alcohol intake:				
Non-drinker	11 985 (6.1)	89 (4.7)	22 415 (9.4)	171 (11.7)
Trivial drinker	41 810 (21.3)	335 (17.7)	96 085 (40.3)	591 (40.5)
Light drinker	57 817 (29.5)	521 (27.5)	76 942 (32.3)	433 (29.7)
Moderate drinker	60 694 (31.0)	624 (32.9)	37 830 (15.9)	234 (16.0)
Heavy drinker	14 960 (7.6)	205 (10.8)	3797 (1.6)	25 (1.7)
Very heavy drinker	8825 (4.5)	121 (6.4)	1427 (0.6)	4 (0.3)
Medical history:				
Ulcerative colitis	1053 (0.5)	17 (0.9)	1211 (0.5)	12 (0.8)
Colorectal polyps	616 (0.3)	11 (0.6)	612 (0.3)	6 (0.4)
Diabetes	12 893 (6.6)	184 (9.7)	7885 (3.3)	62 (4.3)
Breast cancer	NA	NA	9448 (4.0)	71 (4.9)
Uterine cancer	NA	NA	1030 (0.4)	16 (1.1)
Ovarian cancer	NA	NA	724 (0.3)	11 (0.8)
Cervical cancer	NA	NA	1711 (0.7)	10 (0.7)
Lung cancer	125 (0.1)	1 (0.1)	NA	NA
Blood cancers	1146 (0.6)	10 (0.5)	NA	NA
Oral cancer	483 (0.2)	12 (0.6)	NA	NA
Family history of colorectal cancer	19 505 (9.9)	266 (14.0)	22 252 (9.3)	169 (11.6)

IQR=interquartile range; NA=not applicable.

* White and not recorded ethnic groups are combined in the QCancer-10 model but presented separately here for information.

† Not included in model for women but provided for information.

### Polygenic risk score models

Of the six PRS models assessed (supplementary fig S4), LDpred2-grid had the highest odd ratios per standard deviation of PRS (1.584, 95% confidence interval 1.536 to 1.633; table 2) and performed best in the test cohort (fig 1), with a C statistic of 0.717 (0.711 to 0.725) and an R^2^ of 6.3% (5.9 to 6.8%) (table 2). A weak interaction between age and PRS was noted, with a reduced effect size of PRS with increasing age (supplementary table S6, fig S5), but this interaction effect was not included in the models. All genome-wide models performed better than the GWAS significant model, and all PRS showed improved performance over the reference model of age, sex, genotyping array, and four principal components (table 2). Performance without adjustment for age and sex is shown in supplementary table S7. Internal validation showed low bias in all measures as shown in the very little difference between apparent performance and internal validation (table 2).

**Table 2:** Apparent and internally and externally validated polygenic risk score (PRS) performance in logistic regression models (adjusting for age, sex, genotyping array, and first four principal components)

	LDpred2-inf	LDpred2-grid	LDpred2-grid-sp	SCT	C+T	GWAS significant	Reference
Number of single nucleotide polymorphisms	1 104 409	1 104 409	616 956	194 756	13 446	50	NA
Apparent performance							
PRS odds ratio per SD	1.435 (1.391 to 1.480)	1.584 (1.536 to 1.633)	1.571 (1.524 to 1.620)	1.417 (1.375 to 1.461)	1.425 (1.382 to 1.470)	1.390 (1.348 to 1.433)	NA
C statistic	0.704 (0.697 to 0.712)	0.717 (0.711 to 0.725)	0.716 (0.710 to 0.723)	0.702 (0.695 to 0.711)	0.704 (0.697 to 0.711)	0.700 (0.693 to 0.707)	0.680 (0.672 to 0.687)
Dxy	0.407 (0.394 to 0.423)	0.435 (0.422 to 0.451)	0.432 (0.419 to 0.446)	0.404 (0.389 to 0.422)	0.407 (0.394 to 0.423)	0.400 (0.386 to 0.414)	0.359 (0.344 to 0.374)
R^2^ (%)	5.5 (5.1 to 5.9)	6.3 (5.9 to 6.8)	6.2 (5.8 to 6.7)	5.4 (5.0 to 5.9)	5.4 (5.1 to 5.9)	5.3 (4.9 to 5.7)	4.2 (3.8 to 4.6)
R^2^ PRS (%)	1.3 (1.1 to 1.5)	2.1 (1.9 to 2.4)	2.0 (1.8 to 2.3)	1.2 (1.0 to 1.4)	1.2 (1.0 to 1.5)	1.1 (0.9 to 1.3)	NA
Scaled Brier (%)	0.87	1.05	1.03	0.86	0.85	0.83	0.60
Internal validation							
C statistic	0.703	0.717	0.716	0.701	0.703	0.700	0.679
Dxy	0.406	0.434	0.432	0.403	0.406	0.400	0.358
R^2^ (%)	5.4	6.3	6.2	5.4	5.4	5.3	4.2
Calibration slope	0.996	0.997	0.998	0.996	0.995	0.999	0.996
Scaled Brier (%)	0.85	0.94	1.06	0.84	0.76	0.85	0.58
Geographical validation							
C statistic	0.726 (0.704 to 0.748)	0.732 (0.710 to 0.752)	0.733 (0.710 to 0.753)	0.718 (0.696 to 0.739)	0.719 (0.696 to 0.740)	0.703 (0.679 to 0.724)	0.677 (0.654 to 0.699)
Dxy	0.452 (0.408 to 0.496)	0.464 (0.420 to 0.504)	0.466 (0.421 to 0.507)	0.436 (0.392 to 0.477)	0.438 (0.392 to 0.480)	0.405 (0.358 to 0.447)	0.353 (0.308 to 0.397)
R^2^ (%)	7.0 (5.7 to 8.4)	7.6 (6.1 to 8.9)	7.6 (6.1 to 8.9)	6.4 (5.0 to 7.7)	6.6 (5.2 to 7.9)	5.4 (4.0 to 6.7)	3.8 (2.6 to 5.0)
Calibration slope	1.137 (1.010 to 1.268)	1.091 (0.967 to 1.199)	1.104 (0.980 to 1.213)	1.076 (0.946 to 1.200)	1.098 (0.958 to 1.222)	0.994 (0.861 to 1.113)	0.936 (0.795 to 1.075)
CITL	0.206 (0.120 to 0.272)	0.198 (0.113 to 0.262)	0.199 (0.115 to 0.264)	0.194 (0.107 to 0.260)	0.195 (0.110 to 0.261)	0.191 (0.104 to 0.258)*	0.191 (0.105 to 0.257)*
Scaled Brier (%)	1.48	1.64	1.66	1.27	1.38	1.08	0.67
Minority ethnic validation†							
C statistic	0.588 (0.545 to 0.627)	0.602 (0.558 to 0.640)	0.601 (0.559 to 0.640)	0.589 (0.546 to 0.626)	0.597 (0.554 to 0.636)	0.587 (0.543 to 0.624)	0.585 (0.542 to 0.623)
Dxy	0.176 (0.090 to 0.254)	0.203 (0.116 to 0.279)	0.203 (0.118 to 0.281)	0.179 (0.093 to 0.253)	0.195 (0.108 to 0.271)	0.174 (0.086 to 0.247)	0.171 (0.084 to 0.245)
Calibration slope	0.175 (0.096 to 0.258)	0.204 (0.122 to 0.288)	0.208 (0.126 to 0.294)	0.161 (0.088 to 0.240)	0.195 (0.110 to 0.281)	0.143 (0.071 to 0.213)	0.144 (0.069 to 0.217)
CITL	1.299 (1.155 to 1.417)	1.336 (1.194 to 1.456)	1.325 (1.183 to 1.446)	1.360 (1.217 to 1.479)	1.310 (1.167 to 1.429)	1.392 (1.251 to 1.511)	1.343 (1.200 to 1.459)
Scaled Brier (%)	–0.02	0.04	0.03	–0.06	–0.06	–0.07	–0.15

Values are performance indices plus 95% confidence intervals. Internal validation used 500 bootstrap samples. Pairwise comparisons of performance metrics in validation cohorts were all significantly different P<0.001 except comparisons marked. LDpred2-inf=LDpred2 infinitesimal model; LDpred2-grid=LDpred2 grid model; LDpred2-grid-sp=LDpred2 sparse grid model; SCT=stacked clumping and thresholding; C+T=clumping and thresholding; PRS OR per SD=odds ratio per standard deviation of PRS in the age and sex adjusted model; Dxy=Somers’ D_xy_ rank correlation; R^2^=Nagelkerke’s R^2^(explained variation); CITL=calibration-in-the-large.

* P=0.60.

† R^2^ for all models in the minority ethnic validation cohort <0 (indicating poorer performance than a model with no explanatory variables).

In the geographical validation cohort, discrimination and explained variation improved compared with the test cohort for all PRS models. LDpred2-derived models performed best, and all genome-wide models showed improved performance over the GWAS significant model (table 2). All models underpredicted risk (calibration-in-the-large >0; table 2) particularly in the highest PRS groups (fig S6), and genome-wide models were slightly overfitted (calibration slope >1, ie, insufficient variation at the extremes of prediction; table 2, supplementary fig S6).

In subgroup analyses of logistic regression models (supplementary table S8, supplementary fig S7), discrimination and explained variation were better in men; models were better fitted in women but underpredicted risk to a greater extent, particularly in higher risk groups. Discrimination and explained variation were poorer in individuals with a first degree family history of colorectal cancer, with models systematically underpredicting risk across PRS risk groups. All models tended to underpredict risk across age groups, with more marked miscalibration in the 55-59 years age group due to a step in observed risk (supplementary fig S8). PRS performance was poor in the minority ethnic validation cohort (table 2). Models systematically underpredicted risk and were highly over-fitted (ie, predictions were too extreme, table 2), with modest improvement after recalibration (supplementary fig S6). In general, PRS performance in Cox models supported the logistic regression analysis (supplementary tables S9-S10, supplementary figs S9-S14).

### QCancer-10 non-genetic model

Comparative demographics of the original QCancer-10 derivation cohort^4^ and the integrated modelling cohort are shown in supplementary table S11. Notably the integrated modelling cohort is older, less ethnically diverse, has a lower Townsend deprivation score, has fewer smokers, and has higher prevalence of reported family history of colorectal cancer than does the QCancer-10 cohort. Model performance in the integrated modelling cohort (table 3) was concordant with previously published validation studies.^3^ As expected, the model for women did less well than the model for men.^3^ Both models tended to overpredict risk, which was corrected through recalibration, although for women, the model continued to overpredict in the top 10% of risk (supplementary fig S15). In a subgroup analysis, models were well calibrated across age groups; they underpredicted risk in individuals from minority ethnic backgrounds; and the model for women tended to overpredict risk in people with a first degree family history of colorectal cancer, particularly in higher risk groups (supplementary table S12, supplementary figs S16-S17).

**Table 3:** Apparent and internally validated performance of QCancer-10 risk score with LDpred2 sparse grid PRS (QCancer-10+LDP) and QCancer-10 with GWAS significant PRS (QCancer-10+GWS) models, compared with external validation of QCancer-10 in the same participants. Values are performance indices (95% confidence intervals), unless otherwise stated

	QCancer-10+LDP			QCancer-10+GWS		QCancer-10
	Apparent	Internal validation		Apparent	Internal validation	
**Men**						
QCancer-10 HR per SD	2.295 (2.151 to 2.449)	NA		2.302 (2.157 to 2.457)	NA	NA
PRS HR per SD	1.605 (1.531 to 1.683)	NA		1.466 (1.396 to 1.539)	NA	NA
Harrell’s C index	0.730 (0.720 to 0.741)	0.730		0.715 (0.706 to 0.726)	0.715	0.693 (0.682 to 0.704)
Dxy	0.460 (0.440 to 0.481)	0.459		0.430 (0.411 to 0.452)	0.430	0.847 (0.841 to 0.852)
Royston’s D statistic	1.283 (1.224 to 1.342)	1.281		1.201 (1.148 to 1.259)	1.199	1.058 (0.987 to 1.121)
R^2^_D_ (%)	28.2 (26.3 to 30.1)	28.1		25.6 (23.9 to 27.5)	25.6	21.1 (18.9 to 23.1)
Scaled Brier (%)	0.82	0.81		0.79	0.78	0.59
Calibration slope	NA	0.998		NA	0.998	0.995 (0.914 to 1.063)
**Women**						
QCancer-10 HR per SD*	—	NA		1.756 (1.647 to 1.873)	NA	NA
PRS HR per SD*	—	NA		1.368 (1.298 to 1.443)	NA	NA
Harrell’s C index	0.687 (0.673 to 0.702)	0.686		0.669 (0.655 to 0.683)	0.668	0.645 (0.631 to 0.659)
Dxy	0.374 (0.347 to 0.404)	0.372		0.338 (0.310 to 0.367)	0.337	0.822 (0.816 to 0.830)
Royston’s D statistic	1.056 (0.983 to 1.141)	1.055		0.926 (0.852 to 1.002)	0.925	0.769 (0.695 to 0.847)
R^2^_D_ (%)	21.0 (18.7 to 23.7)	21.0		17.0 (14.8 to 19.3)	17.0	12.4 (10.3 to 14.6)
Scaled Brier (%)	0.34	0.34		0.28	0.28	0.20
Calibration slope	NA	0.996		NA	0.996	0.805 (0.724 to 0.899)

QCancer-10 HR per SD=adjusted hazard ratio of QCancer-10 score; NA=not available; PRS HR per SD=adjusted hazard ratio per standard deviation of the PRS; Dxy=Somers’ D_xy_ rank correlation; R^2^_D_=Royston and Sauerbrei’s R^2^_D _(explained variation). Pairwise comparisons of performance metrics were all significantly different P<0.001.

* Modelled using multiple fractional polynomials in QCancer-10+LDP model and therefore not presented.

### QCancer-10+PRS models

Given the similarities in performance of LDpred2-grid and LDpred2-grid-sp models, we selected LDpred2-grid-sp as the top performing genome-wide PRS for integrated modelling with QCancer-10, favouring sparsity (ie, a PRS containing fewer single nucleotide polymorphisms; see supplementary results for full model specifications and baseline hazards). We found no evidence of interactions between QCancer-10 and PRS terms in the models (supplementary table S13, fig S18).

**Fig 3:**
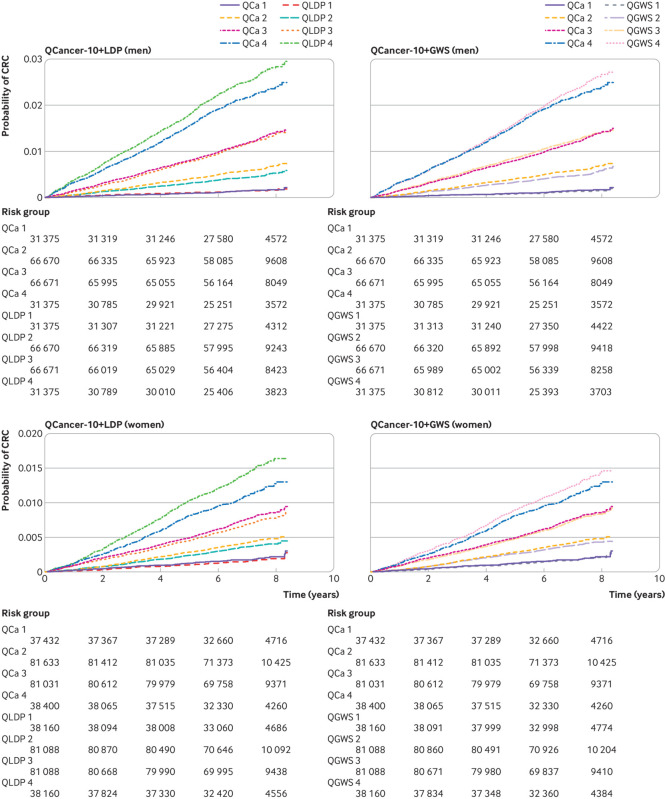
Kaplan-Meier cumulative incidence curves across four risk groups (group 4 being highest risk) for QCancer-10 risk score with LDpred2 sparse grid PRS (QCancer-10+LDP) and QCancer-10 risk score with GWAS significant PRS (QCancer-10+GWS) models compared with QCancer-10 in men and women. CRC=colorectal cancer; QCa=QCancer-10 model; QLDP=Qcancer-10+LDP model; QGWS=QCancer-10+GWS model

Cox models combining the QCancer-10 risk score with LDpred2 sparse grid model (QCancer-10+LDP), and the GWAS significant PRS (QCancer-10+GWS) both outperformed QCancer-10 (table 3). Figure 3 shows Kaplan-Meier curves across four risk groups in integrated QCancer-10+PRS models compared with QCancer-10 alone, showing improved separation between risk groups with the addition of PRS. Internal validation of the QCancer-10+PRS models showed very little optimism in performance estimates. Sensitivity analysis excluding cancer cases diagnosed within two years of recruitment did not support a significant effect of reverse causality (supplementary table S14).

Models predicting risk in men had better discrimination and explained more of the variation in risk than models for women (table 3). Calibration by age was good in these models (supplementary fig S16), with slight underprediction of risk in the oldest age group in women. Discrimination and explained variation were higher for each model in people younger than 50 years compared with older age groups, and improvements in model performance in Cancer-10+PRS models compared with the QCancer-10 model were more marked in the youngest age groups. For example, QCancer-10+LDP explained 11.9% more variation than QCancer-10 in men younger than 50 years, compared with 7.6% more variation in men older than 60 years; in women, these figures were 15.8% compared with 9.0% (supplementary table S15). As with QCancer-10, in people with a first degree family history of colorectal cancer, QCancer-10+PRS models for women tended to overpredict risk, particularly in higher risk groups, whereas male QCancer-10+PRS models were well calibrated (supplementary table S12, supplementary fig S17). In minority ethnic groups, QCancer-10+PRS models underpredicted risk (expected to observed risk ratio of <1; supplementary table S12) to a greater extent than QCancer-10, subject to the caveat of low colorectal cancer case numbers (46 in men, 58 in women) in this subgroup; calibration was excellent for white participants (expected/observed risk=1).

QCancer-10+LDP consistently provided the best risk prediction. Table 4 shows the sensitivity, specificity, detection rate, and false positive rate of the Qancer-10+LDP model in predicting colorectal cancer risk across the top quarter of absolute risk. To illustrate, individuals predicted to be in the top 20% of absolute risk by QCancer-10+LDP accounted for 47.8% of cases in men and 42.7% of cases in women, with detection rates of 0.46% and 0.26% respectively. QCancer-10 and QCancer-10+GWS had lower sensitivity and slightly lower detection rates than QCancer-10+LDP; the difference was minimal in specificity or false positive rates (supplementary tables S16-S19). Men in the top 5% of absolute risk by the QCancer-10+LDP model had more than 3.47-fold increased absolute five year risk compared with the median, with a comparable 2.77-fold increase in women. For QCancer-10+GWS, this figure was 3.06-fold in men and 2.35-fold in women, and for QCancer-10 was 2.37-fold in men and 2.06-fold in women (supplementary table S20). Differences in absolute risk predicted by the models for a given risk quantile were small. For example, the difference in five year absolute risk threshold between QCancer-10+LDP and QCancer-10 models for the top 5% of highest risk was 0.34% in men and 0.15% in women.

**Table 4:** Sensitivity, specificity, detection rate, and false positive rate of QCancer-10 risk score with LDpred2 sparse grid PRS (QCancer-10+LDP) models for colorectal cancer diagnosis across the top 25% of absolute risk in men and women

Percentage	Population per percentage	Absolute five year risk centile cut-off (%)	Cases per percentage	Cumulative % cases based on absolute risk (sensitivity)	Specificity (%)	Detection rate (%)
**Men**						
1	1960	2.75	68	3.6	99.0	0.03
2	1961	2.33	59	6.7	98.0	0.06
3	1961	2.10	61	9.9	97.1	0.10
4	1961	1.94	69	13.5	96.1	0.13
5	1961	1.81	55	16.4	95.1	0.16
6	1961	1.71	62	19.7	94.1	0.19
7	1961	1.62	43	22.0	93.1	0.21
8	1961	1.55	53	24.8	92.2	0.24
9	1961	1.49	37	26.8	91.2	0.26
10	1961	1.43	36	28.7	90.2	0.28
11	1961	1.38	49	31.3	89.2	0.30
12	1960	1.33	53	34.1	88.2	0.33
13	1961	1.29	34	35.9	87.2	0.35
14	1961	1.25	31	37.5	86.2	0.36
15	1961	1.21	39	39.6	85.2	0.38
16	1961	1.17	28	41.1	84.2	0.40
17	1961	1.14	38	43.1	83.3	0.42
18	1961	1.11	26	44.5	82.3	0.43
19	1961	1.08	34	46.3	81.3	0.45
20	1961	1.05	28	47.8	80.3	0.46
21	1961	1.03	25	49.1	79.3	0.47
22	1961	1.00	39	51.2	78.3	0.49
23	1960	0.98	30	52.8	77.3	0.51
24	1961	0.95	26	54.2	76.3	0.52
25	1961	0.93	26	55.6	75.3	0.53
**Women**						
1	2384	1.54	58	4.0	99.0	0.02
2	2385	1.27	48	7.3	98.0	0.05
3	2385	1.14	50	10.7	97.0	0.07
4	2385	1.04	36	13.2	96.1	0.08
5	2385	0.97	43	16.1	95.1	0.10
6	2385	0.92	28	18.0	94.1	0.11
7	2385	0.87	36	20.5	93.1	0.13
8	2385	0.83	33	22.8	92.1	0.14
9	2385	0.80	38	25.4	91.1	0.15
10	2385	0.77	27	27.3	90.1	0.17
11	2385	0.74	28	29.2	89.1	0.18
12	2385	0.72	26	31.0	88.1	0.19
13	2385	0.70	27	32.9	87.1	0.20
14	2385	0.68	20	34.3	86.1	0.21
15	2385	0.66	26	36.1	85.1	0.22
16	2385	0.64	21	37.5	84.1	0.23
17	2385	0.63	15	38.5	83.1	0.23
18	2385	0.61	15	39.5	82.1	0.24
19	2385	0.60	24	41.1	81.1	0.25
20	2385	0.59	23	42.7	80.1	0.26
21	2385	0.57	19	44.0	79.1	0.27
22	2385	0.56	20	45.4	78.1	0.28
23	2385	0.55	18	46.6	77.1	0.28
24	2385	0.54	23	48.2	76.1	0.30
25	2385	0.53	17	49.4	75.1	0.30

By way of illustrating a possible clinical use of the model, enhanced screening is frequently offered for people with at least one first degree relative with colorectal cancer, corresponding to an about 2.2-fold increased risk.^30^ QCancer-10+LDP identified 18.4% of men (34.5% of cases) and 7.4% of women (16.7% of cases) as having a relative risk of more than 2.2, of whom 76.0% and 69.8%, respectively, had no first degree relative with colorectal cancer (see supplementary table S21 for equivalent values for QCancer-10+GWS and QCancer-10).

**Fig 4:**
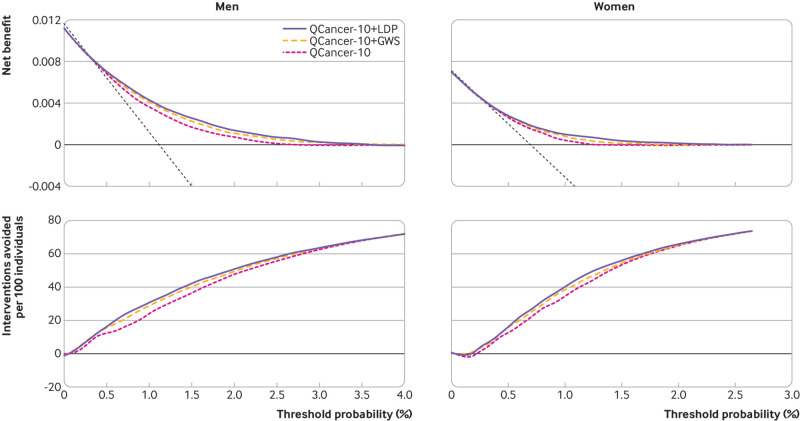
Decision Curve Analysis for QCancer-10 risk score with LDpred2 sparse grid PRS (QCancer-10+LDP), QCancer-10 risk score with GWAS significant PRS (QCancer-10+GWS), and QCancer-10 models. Figures show net benefit and unnecessary interventions avoided per 100 individuals tested in men (left) and women (right), calculated at eight years of follow-up. The dashed black line in net benefit curves indicates intervention for all, the thick black line no intervention

Illustrative decision curve analyses supported the findings that, across a range of threshold probabilities, QCancer-10+LDP gave greater net benefit than did QCancer-10+GWS and QCancer-10, for both men and women, and predicted a greater number of interventions avoided across clinically relevant thresholds (fig 4). Taking a threshold probability of 1%, the net benefit for QCancer-10+LDP in men is 0.00430 true positives, or 0.4 net detected cancers without an increase in unnecessary colonoscopies per 100 individuals. In women, these values were 0.00098 true positives (0.1 net cancers per 100 individuals). The difference in net benefit between the QCancer-10+LDP model and QCancer-10 model was 0.00068 for men, equating to a test trade-off of 1478 tests (ie, 1478 PRS tests needed to detect one additional cancer over the QCancer-10 model), and 0.00056 for women, equating to a test trade-off of 1789 tests. This finding indicates that use of PRS on 1478 men or 1789 women would detect one additional cancer over using the QCancer-10 model alone. Analysis of interventions avoided at the same threshold showed that using QCancer-10 to risk stratify 100 individuals would save 24.2 colonoscopies for men and 34.9 for women, for the same number of cancers detected, compared with the baseline approach of colonoscopy for all individuals. Adding the LDpred2 PRS, would save an additional 6.7 colonoscopies for men and 5.5 for women per 100 individuals, compared with using QCancer-10 alone. Net benefit, test trade-off, and interventions avoided at additional prespecified threshold probabilities are presented in supplementary tables S22 and S23.

## Discussion

### Principal findings

We have developed and validated new prediction models for colorectal cancer that combine phenotypic risk with genome-wide PRS.^6^ ^9^ QCancer-10+LDP performed best across all metrics. The sensitivities realised using QCancer-10+PRS (particularly QCancer-10+LDP) exceed those of QCancer-10 alone and of other integrated models validated in UK Biobank.^6^ Although QCancer-10+PRS risk models could provide more accurate information for screening decisions, the extent of the improvement in performance obtained through adding PRS must be carefully considered. QCancer-10^4^ can be derived from existing health records and has been recommended for guiding shared decision making around colorectal cancer screening.^5^ However, these benefits need to take into consideration the logistical implications, cost, and potential ethical issues of implementing PRS-based screening.

The incremental benefit of PRS over use of QCancer-10 alone is modest. The increase in C statistic is 0.04 (although this statisticis notoriously difficult to shift for models even with the addition of a predictor with a large effect size).^31^ The improvement in explained variation was 7.1% in men and 8.4% in women. A greater improvement in explained variation was noted with the addition of PRS in men and women younger than 50 years, potentially reflecting the greater influence of genetic predisposition at this age. Of note, clinical decision making is generally driven by absolute risk,^32^ and the difference in absolute risk discerned by QCancer-10+PRS over QCancer-10 at a given risk threshold is small (eg, 0.34% difference in five year colorectal cancer risk for men and 0.15% for women in the top 5% highest risk group). Detection rates were modestly improved with the addition of PRS. Furthermore, our test trade-off calculations under a simplified scenario of colorectal cancer screening by colonoscopy alone every eight years showed that a large number of PRS tests might need to be performed for each additional cancer detected by adding the LDpred2 PRS to QCancer-10. We caution that this analysis has several limitations and is illustrative only: for example, we used a simple (albeit widely used) measure of test net benefit and we did not consider the longitudinal nature of screening, where the costs of PRS are incurred with the first screening round, whilst the benefit applies to successive rounds. Nevertheless, considerable infrastructural change would be needed to implement PRS assessment within a national screening programme, in contrast to QCancer-10 alone, which could be relatively easily implemented through primary care data at far lower cost. Overall, our findings raise concerns that the relatively small benefits reported of adding PRS will be insufficient to warrant implementation. We note, however, that PRS could become part of population health records, making implementation more feasible, should current initiatives such as Our Future Health come to fruition.^33^

Of the PRS methods evaluated, LDpred2-grid and LDpred2-grid-sp models had the highest discrimination, explained more of the variation in risk, and were well calibrated. The improvement in performance between the derivation and validation cohorts when using the PRS models probably results from lower genetic homogeneity in the validation cohort. Evaluation of the PRS in a geographically external cohort shows portability of the PRS models. The geographical validation cohort was well matched in age to the derivation cohort but had a higher proportion of women and prevalence of colorectal cancer was higher at 1.79% compared with 1.51% in the derivation cohort. All models tended to underpredict risk in the top risk group in the geographical validation cohort, probably due to demographic differences between the cohorts, which improved with recalibration. This miscalibration was greater for women, which might reflect a greater difference in demographics and cancer risk between derivation and validation cohorts for women compared with men. In addition, we noted some miscalibration by age due to a jump in observed risk in the 55-64 year age groups (supplementary figs S8 and S12). This increase could be due to early detection of prevalent colorectal cancer on entry to the bowel screening programme, which begins at 50 in Scotland compared with 60 in the rest of the UK during the study period. This miscalibration was not seen in the integrated modelling cohort. We would expect performance for northern European individuals in the general population to be similar to that of the validation cohort.

### Comparison with other studies

Our PRS findings are in line with a recent study in which a PRS derived using LDpred software (an earlier version of LDpred2) out-performed both machine learning approaches and a 140 GWAS significant single nucleotide polymorphism PRS.^7^ Previous studies have found that models combining GWAS significant PRS and non-genetic risk predictors perform better than PRS alone^6^ or non-genetic risk factors alone.^9^ Our work supports and extends this finding by showing the stepwise improvement in performance obtained with genome-wide PRS. A key strength of our study is the avoidance of overlap between our GWAS meta-analysis datasets and modelling cohorts, thus reducing overfitting of the PRS and performance optimism.^10^ We used expected genotype dosages rather than allele counts in each PRS, incorporating uncertainty in genotype imputation, and applied correction for ascertainment bias to effect sizes in the GWAS significant model. Our GWAS significant PRS used stringent inclusion criteria, including only single nucleotide polymorphisms that were replicated in our GWAS after excluding UK Biobank samples.

### Strengths and limitations of this study

The UK Biobank study provides a large sample size, extensive phenotyping, data completeness, and linkage to external datasets. In general, UK Biobank represents a healthy population with, for example, lower prevalence of smoking and of most self-reported health conditions than in national Health Survey for England data.^34^ Whilst self-reported exposures are subject to misclassification bias, they are often used clinically. Linkage to cancer registry data in UK Biobank ended in 2015-16 at the time of analysis so follow-up is limited to a median of seven years. The UK Biobank study age range of about 40-70 years is similar to the age of people eligible for bowel cancer screening (soon to be 50-74 in both England and Scotland), although narrower than the range 25-84 years that was used in the original QCancer-10 study.^4^ However, model performance in UK Biobank is arguably unlikely to reflect relative performance in the general population, for several reasons. 

Firstly, because the UK Biobank study has a lower incidence of disease than does the general population of screening age, sensitivity is expected to be higher in the screening population, which is at higher risk.^35^ Secondly, all of our models appeared to perform less well in women. For PRS, wide confidence intervals in the geographical validation cohort mean that this finding should be interpreted with caution; however, for models that include QCancer-10, this difference was not unexpected because the known healthy volunteer bias in UK Biobank is especially marked in women.^34^ Thirdly, the available sample size and number of incident cases for women in our integrated modelling cohort fell slightly short of requirements (supplementary methods). As a result, our estimates of risk might be less precise for women, and further validation is essential before implementation. Fourthly, the QCancer-10 model performs worse when validated in UK Biobank than in the QResearch validation cohort.^4^ We suspect that this effect is due to the differences in age distribution between the general population sample used to develop the original QCancer-10 score and the more restricted UK Biobank study sample.^36^ Overall, further risk model development and evaluation should occur in a population representative of the screening population. 

Further limitations of our study might include unknown differences in the demographics of the contributing base GWAS datasets and UK Biobank. Additionally, we did not include mendelian colorectal cancer syndromes in the genetic model, probably resulting in poorer calibration in people with a family history of colorectal cancer.^37^ Furthermore, detailed information on colorectal polyp diagnosis and pathology is not available in UK Biobank at present, and therefore, we were unable to evaluate colorectal cancer precursors, such as advanced adenoma, as an outcome. Another major limitation of our study, and PRS generally, is that most models are developed in individuals of European ethnicity. Although most colorectal cancer risk single nucleotide polymorphisms appear to be shared across ethnic groups, quantitative risk estimates cannot readily be transferred across populations,^38^ and, as anticipated, our PRS performed poorly in the minority ethnic validation cohort. As minority ethnic populations often have higher mortality associated with colorectal cancer and lower screening uptake, further work is urgently needed to expand PRS for colorectal cancer in these populations to avoid exacerbating existing health inequalities.^38^ ^39^ ^40^

### Policy implications

In existing cancer screening programmes in the general population, the risk models perform at a level that might be clinically useful.^32^ About 10% of the study population aged 40-70 years have predicted relative risks of colorectal cancer that are high enough (>2.2-fold) to warrant enhanced colonoscopic surveillance under guidelines that are used for individuals at high familial risk.^41^ A single risk threshold for enhanced colorectal cancer screening could be established across the entire population, although resource considerations imply a much higher threshold than is currently in use, or a primary screening method other than colonoscopy, or both. For people below the enhanced screening threshold, use of the risk score would be adapted to existing screening programmes. For example, risk scores derived from primary care data, with or without PRS from saliva samples, could be used alongside faecal immunochemical testing to decide who proceeds to colonoscopy (with a lower threshold for positive faecal immunochemical testing in people at higher risk), so that universal access to screening is maintained, whilst improving performance. An alternative or complementary approach would be to for individuals to undertake risk profiling at 40 years, allowing younger individuals at high risk to begin screening earlier, and so addressing the increasing incidence of early onset colorectal cancer. Our analysis by age group shows that the greatest improvements with the addition of PRS are noted in people younger than 50 years. Detailed assessment of these and any other strategies for risk score use will be essential (eg, the positive predictive value of faecal immunochemical testing has been shown to vary by PRS based risk 42 and the added value of risk scores to faecal immunochemical testing could be low).

### Conclusions

Colorectal cancer is arguably the best placed of all cancers to benefit from stratified screening. Although we have shown that risk stratification in some form is likely in principle to improve resource use and performance of colorectal cancer screening, the added benefit of adding PRS to QCancer-10 is modest, and we find no clear justification for implementing PRS based risk stratification at present. Risk assessment, particularly PRS, also has the potential to reduce screening participation and widen existing health disparities. Thus, if the potential clinical benefit of our integrated risk model is deemed acceptable to policy makers, a thorough real world evaluation of both QCancer-10 and QCancer-10+PRS, including cost effectiveness, should be undertaken before implementation. We contend that an introduction of risk stratified screening for colorectal cancer or other common cancers is premature without a full assessment alongside current screening methods in a cohort representative of the screening population.

**What is already known on this topic**

Risk stratification based on genetic or environmental risk factors could improve cancer screening outcomes
No previously published study has examined integrated models combining genome-wide polygenic risk scores and non-genetic risk factors beyond age
QCancer-10 (colorectal cancer) is the top performing non-genetic risk prediction model for colorectal cancer

**What this study adds**

Adding PRS to the QCancer-10 (colorectal cancer) risk prediction model modestly improves performance and clinical benefit, with greatest gain from the LDpred2 genome-wide PRS
The challenges and costs of implementing stratification based on polygenic risk scores in population screening might not be justified by the incremental benefit over QCancer-10 alone
Detailed real world evaluation, including value added to screening regimens, economic assessment, and effects on participant participation are needed before PRS implementation

## Data Availability

UK Biobank data can be obtained through https://www.ukbiobank.ac.uk/. Genotype data are available in the European Genome-phenome Archive under accession numbers EGAS00001005412, EGAS00001005421, or from the Edinburgh University DataShare Repository (https://datashare.ed.ac.uk/). Finnish cohort samples can be requested from the THL Biobank https://thl.fi/en/web/thl-biobank. PRS single nucleotide polymorphism inclusion lists and model specifications will be deposited in the PGS catalogue repository (https://www.pgscatalog.org/). Risk scores for UK Biobank study participants will be returned to UK Biobank for use by approved researchers.

## Web extra

Extra material supplied by authors

Web appendix: Online appendix

## Notes

Contributors: All authors contributed to study conception and design, with development of polygenic risk scores and statistical analysis led by SEWB, IT, and JH-C. IT, MD, and RH provided data. SEWB, IT, PL, and RH have accessed and verified the underlying data. SEWB carried out primary data analysis. SEWB completed the statistical analysis under supervision of IT and JH-C, SW and JEE supervised the project. SEWB and IT wrote the first draft of the manuscript. All authors contributed to critical revision and editing of the manuscript, and have approved the final version. JH-C and IT contributed equally, and are guarantors. The corresponding author attests that all listed authors meet authorship criteria and that no others meeting the criteria have been omitted.

The lead author (IT) affirms that the manuscript is an honest, accurate, and transparent account of the study being reported; that no important aspects of the study have been omitted; and that any discrepancies from the study as planned (and, if relevant, registered) have been explained.

Dissemination to participants and related patient and public communities: Dissemination to the general public and clinicians will be provided through social media, an online plain language summary, and a video discussion (vlog) through The Centre for Personalised Medicine at the University of Oxford.

Provenance and peer review: Not commissioned; externally peer reviewed.
