# Supplementary material for: Integrating genome-wide polygenic risk scores and non-genetic risk to predict colorectal cancer diagnosis using UK Biobank data: population based cohort study
Source: BMJ. 2022 Nov 9;379:e071707. doi: 10.1136/bmj-2022-071707 (PMC9644277; doi:10.1136/bmj-2022-071707)
Supplement: Supplementary file 1 — Web appendix: Online appendix [file bris071707.ww.pdf]

Supplementary Material

Integrating genome-wide polygenic risk scores and non-genetic risk to predict colorectal cancer diagnosis: a cohort study in UK Biobank

Table of Contents

Supplementary Methods *4*

Base Genome-wide Association Study Meta-analysis 4

Cancer Incidence Calculation 4

PRS Sample QC and dataset definitions 4

Figure S1. SNP QC 5

GWAS significant PRS 6

C+T and SCT PRS 6

LDpred2 PRS 6

Table S1: Single nucleotide polymorphisms included in the GWAS-significant risk model 7

Figure S2. Z-scores for LDpred2-grid PRS calculated across a grid of tuning parameters 8

Evaluation of polygenic risk score performance 8

Validation of QCancer-10 8

Table S2: Mapping of UK Biobank ethnicity, smoking and alcohol data to QCancer-10 coding 9

Table S3: UK Biobank codes self-reported medical history for QCancer-10 predictors 10

Integrated model development 10

Decision Curve Analysis 10

Software 11

Supplementary Results *12*

Table S4: Characteristics and missingness of predictor values for the whole UKB cohort, excluding individuals with prevalent CRC 12

Table S5: Demographics of derivation and validation cohorts used in PRS development 14

Figure S3. Age specific CRC rates 15

Figure S4. Distributions of standardised PRS for PRS Test Cohort and Validation Cohorts. 16

Interactions between PRS and Age 17

Table S6. Wald $\chi 2$ of interaction terms between PRS and age in logistic regression and Cox models 17

Figure S5. Marginal effect of standardised PRS in interaction with age 17

Table S7. Apparent performance of PRS assessed in logistic regression models in the Test Cohort, with and without adjustment for sex and age 18

Figure S6. Calibration plots of PRS models in logistic regression analyses in Validation Cohorts. 19

Table S8. Subgroup analysis of PRS logistic regression model performance 20

Figure S7. Calibration of PRS in logistic regression models in subgroup analysis 21

Figure S8. Observed and predicted risk of CRC for PRS logistic regression models across 5 year age bands (in the Geographic Validation Cohort). 22

Table S9. Apparent, internally and externally validated polygenic risk score (PRS) performance in Cox's proportional hazards models (adjusting for age, sex, array and first 4 principal components). 23

Figure S9. Kaplan-Meier cumulative incidence curves across four risk groups (group 4 being highest risk) for PRS in the Geographic Validation Cohort compared with the Test Cohort 24

Figure S10. Calibration plots of PRS models in Cox models in the Geographic Validation Cohort 25

Table S10. Subgroup analysis of PRS Cox model performance by sex 26

Figure S11. Calibration of PRS in Cox models by sex in the Geographic Validation Cohort. 27

Figure S12. Observed and predicted risk of CRC for PRS Cox models across 5 year age bands in the Geographic Validation Cohort 28

Figure S13. Kaplan-Meier cumulative incidence curves across four risk groups (group 4 being highest risk) for PRS in the Minority Ethnic Validation Cohort compared with the Test Cohort 29

Figure S14. Calibration plots of PRS models in Cox model in the Minority Ethnic Validation Cohort. 30

Table S11. Characteristics of the UKB Integrated Modelling Cohort used for QCancer-10 validation, compared with the QCancer-10 derivation cohort 31

Figure S15. Calibration of QCancer-10 over 5-8 years of follow-up. 32

Table S12. Expected/observed ratio of risk over 5-8 years of follow-up for male and female for QCancer-10+LDP, QCancer-10+GWS, and QCancer-10 models in subgroup analyses. . 33

Figure S16. Calibration by age for QCancer-10+LDP, QCancer-10+GWS, and QCancer-10 models. 34

Figure S17. Calibration plots for individuals with a first-degree family history of CRC in QCancer-10+PRS and QCancer-10 models. 35

QCancer-10+PRS model specification 36

Table S13. Interaction terms in QCancer-10+LDP and QCancer-10+GWS models. 36

Figure S18. Marginal effect of QCancer-10 risk score in interaction with PRS in male QCancer-10+LDP and QCancer-10+GWS models 36

Table S14. Sensitivity analysis of QCancer-10+LDP, QCancer-10+GWS, and QCancer-10 model performance with CRC cases diagnosed within 2 years of recruitment excluded from the analysis 38

Table S15. Performance of QCancer-10+LDP, QCancer-10+GWS, and QCancer-10 models across age groups (<50 years, 50-59 years, ≥60 years) 39

Table S16. Sensitivity, specificity, detection rate, and false positive rate of QCancer-10+GWS models for CRC diagnosis across top 25 centiles of absolute risk 40

Table S17. Sensitivity, specificity, detection rate, and false positive rate of QCancer-10 for CRC diagnosis across top 25 centiles of absolute risk 41

Table S18. Sensitivity, specificity, detection rate, and false positive rate of QCancer-10+LDP across top 25 centiles of relative risk 42

Table S19. Sensitivity, specificity, detection rate, and false positive rate of QCancer-10+GWS across top 25 centiles of relative risk 43

Table S20. Fold-increase in absolute risk between 95th centile and median risk for QCancer-10+LDP, QCancer-10+GWS, and QCancer-10 models 44

Table S21. Percentage of population and cases with relative risk > 2.2 for QCancer-10+LDP, QCancer-10+GWS, and QCancer-10 models 44

Table S22. Net benefit and test trade-off across threshold probabilities of 0.5% to 2% for QCancer-10+LDP, QCancer-10+GWS, and QCancer-10 models 45

Table S23. Unnecessary interventions avoided per 100 individuals across threshold probabilities of 0.5% to 2% for QCancer-10+LDP, QCancer-10+GWS, and QCancer-10 models 45

References 46

Supplementary Methods

Base Genome-wide Association Study Meta-analysis

The base dataset for polygenic risk score (PRS) development was obtained through meta-analysis of the datasets included in Law *et al.*,[1] excluding the UK Biobank dataset. Summary data from the following genome-wide association study (GWAS) datasets was therefore included: NSCCG-OncoArray; SCOT; SOCCS/GS; SOCCS/LBC; CCFR1; CCFR2; COIN; CORSA; Croatia; DACHS; FIN; UK1; Scotland1; VQ58. The contributing datasets, genotyping and imputation information, quality control (QC) and study approvals are described in detail in Law *et al.*[1]

Cancer Incidence Calculation

Whole UKB cohort CRC incidence rates were calculated based on linked registry cases, without removal of prevalent cases, to reflect registration as would occur in national data. In addition ASIRs were calculated in the Integrated Modelling Cohort, in which prevalent cases were removed and cases identified through cancer and death registry, and linked hospital inpatient data; follow-up duration was as defined in the main methods. This analysis used R packages 'survival' and 'epitools'.[2,3]

PRS Sample QC and dataset definitions

We performed standard per-person QC on all individuals with imputed genetic data available, removing those with sex chromosome aneuploidy, sex-mismatch and an excess of relatives in the dataset. The Derivation Dataset (see Figure 1) included individuals identified by UKB as having white-British ancestry (on the basis of self-report and principal components analysis), and recruited through English and Welsh centres. We performed further QC on this cohort,[4] removing those who were not included in the PCA calculation (which removes related individuals at 3 degrees of relatedness or closer from the dataset), and restricting further to a genetically homogeneous subset (those within log-distance of 5 following computation of a robust Mahalanobis distance), resulting in a dataset of 310664 individuals.

The Geographic Validation Cohort comprised 34152 individuals recruited in Scotland and of European ancestry (UK Biobank self-reported ethnicities of "British", "Irish", "White", and "Any other white background") passing standard QC. Scotland was chosen for validation as this cohort contained more than the recommended number of cases for model validation (a minimum of 100, and ideally 200, cases),[5] and represents a population with different demographics to England and Wales, testing the models portability.

A Minority Ethnic Validation Cohort (n = 27503) comprised all UK Biobank participants passing standard QC with self-reported ethnicities not in the above categories (including individuals who responded "Do not know" and "Prefer not to answer", but not those with missing ethnicity data).

Thirty thousand randomly selected individuals from the white-British Derivation Dataset were used to derive a Training Cohort for PRS hyper-parameter selection. Ten thousand individuals from within this Training Cohort were used for linkage disequilibrium (LD) matrix construction (used for C+T, SCT and LDpred2 models).[4] The remaining 280664 individuals in the Derivation Dataset comprised the Test Cohort in which PRS performance was evaluated.

We used imputed SNP allele dosage data from UK Biobank, restricting variants to those included in HapMap3, and with matched SNPs in the base data. Of 12972739 SNPs present in the base GWAS summary statistics, 1798524 ambiguous SNPs were removed and 1117002 variants matched with UK Biobank data. QC was performed as recommended by Privé *et al.*[4] on the summary statistics, comparing standard deviations of genotypes in the summary statistics (SD_{ss}) and 10000 individuals from the LDpred2 Training Cohort (SD_{ldtr}), and removing variants where $SD_{ss} < 0.5(SD_{ldtr})$, $SD_{ss} > 0.1 + SD_{ldtr}$, $SD_{ss} < 0.1$, or $SD_{ldtr} < 0.05$ (Figure S1),[4] leaving 1104409 SNPs included in analysis. Following QC, the minimum INFO score was 0.411.

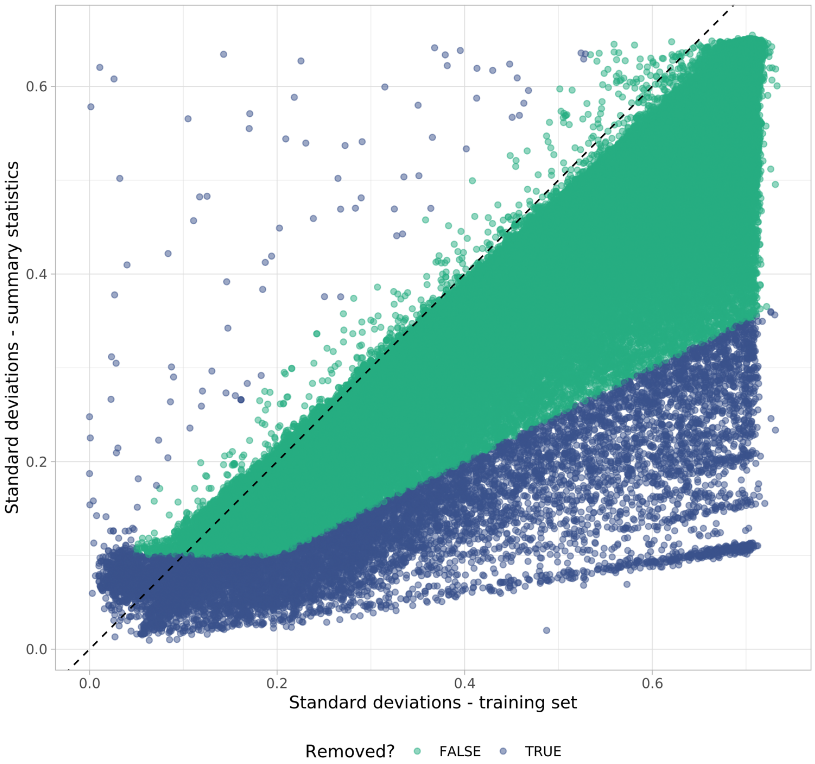

Figure S1. SNP QC based on standard deviations of genotypes in LDpred2 'validation' dataset and base data summary statistics (after Privé et al.)

GWAS significant PRS

We manually curated a list of SNPs derived from previously published GWAS in European populations including Law *et al.* and Huyghe *et al.*[1,6] and the references within these (Table S1). We excluded SNPs which did not reach genome-wide significance ($p<5x10^{-8}$) in our base meta-analysis, and used the effect sizes from our meta-analysis, adjusted for the winner's curse using the False Discovery Rate Inverse Quantile Transformation (FIQT) method.[7] Where SNPs were reported at the same loci in different studies and were correlated at $r^2>0.1$ we retained the most significantly associated SNP. We confirmed that all of the included SNPs imputed well in the UKB data with INFO scores > 0.9. The PRS was calculated as the sum of allele dosages weighted by their effect sizes.

C+T and SCT PRS

Clumping and thresholding approaches to SNP selection generate PRS scores across a range of LD r^2 values (with a given window size for clumping selected) and association p value thresholds. We used R package *bigsnpr* by Privé et al.[8] to generate scores across a grid of r^2, p-value threshold, and clumping window size values. Default parameters were used: clumping $r^2$of 0.01, 0.05, 0.1, 0.2, 0.5, 0.8, 0.95; 50 p-value thresholds spaced equally between 0.1 and the most significant p-value on the log scale; and a base clumping window size of 50, 100, 200 and 500 (where actual window size in kb is the base size divided by clumping r^2).

From this grid, a maximum score was selected based on AUC (the C+T score in this paper), and stacking used to learn the optimal linear combination of scores generated through efficient penalised regression (the SCT score).[8]

LDpred2 PRS

LDpred2[4] uses a Bayesian approach to SNP selection and shrinkage for PRS, based on an LD matrix and GWAS summary statistics, implemented in the R package bigsnpr. This updated version of LDpred has been demonstrated to provide higher predictive performance, particularly with large GWAS sample size as in this study,[4] and also addresses previous instability issues.[9] The use of a larger window of 3cM (using genetic distance rather than number of bases) improves performance when causal variants are located in regions of long-range LD, such as HLA regions. Colorectal cancer-associated variants in these regions have recently been reported,[1,6] and this improvement may therefore be of benefit in CRC-prediction. LDpred2 also evaluates more hyper-parameters (a grid of 126 instead of 7 in LDpred).

There are multiple options for PRS construction within LDpred2. An infinitesimal model (LDpred2-inf), in which all makers are assumed to be causal; grid models (LDpred2-grid) in the hyper-parameters SNP heritability, h^2, proportion of causal variants, *p,* and optionally sparsity, are tuned in a validation set; and an auto model (LDPred2-auto) in which sparsity and SNP heritability are estimated automatically, negating the need for a validation set. LDpred2 estimates heritability calculated from constrained LD score regression. The estimate for this dataset was 0.1602065.

We evaluated LDpred2-inf and LDpred2-grid models (sparse and non-sparse), running them genome-wide as recommended. LDpred2-grid outputs SNP effect sizes for each of the grid values; the optimally performing model was then selected based on best Z-score for the logistic regression slope (Figure S2), in which we adjusted for array platform and first 4 principal components (PCs).

Clumping and thresholding and LDpred2 modelling code was adapted from code provided by Privé *et al.* at https://github.com/Privéfl/paper-ldpred2/tree/master/code, and their accompanying LDpred2 tutorial.[4]

Table S1: Single nucleotide polymorphisms included in the GWAS-significant risk model. OR – odds ratio

rsID	Locus	Chromosome	Position	Risk Allele	P	Beta	OR
rs61776719	1p34.3	1	38461319	C	4.13x10-8	0.071137938	1.07
rs12143541	1p32.3	1	55247852	G	3.39x10-8	0.095046583	1.10
rs4546885	1q25.3	1	183025555	G	1.72x10-11	0.083451882	1.09
rs6658977	1q41	1	222049820	T	3.97x10-8	0.069390267	1.07
rs13020391	2q35	2	219184436	C	2.78x10-10	0.080517316	1.08
rs35470271	3p22.1	3	40915239	G	3.15x10-9	0.099473272	1.10
rs12635946	3q13.2	3	112916918	C	4.16x10-12	0.087357462	1.09
rs17035289	4q24	4	106048291	T	7.03x10-10	0.099808681	1.10
rs75686861	4q31.21	4	145621328	A	1.89x10-8	0.117767505	1.12
rs1445011	5p13.1	5	40280202	C	7.05x10-13	0.094764335	1.10
rs639933	5q31.1	5	134467751	C	3.44x10-8	0.072046433	1.07
rs16878812	6p21.31	6	35569562	A	4.34x10-8	0.106248	1.11
rs1321310	6p21.2	6	36623124	C	7.65x10-10	0.086498504	1.09
rs62404966	6p12.1	6	55712124	C	2.88x10-8	0.079004514	1.08
rs6928864	6q21	6	105966894	C	1.37x10-8	0.125385062	1.13
rs3801081	7p12.3	7	47511161	G	6.28x10-9	0.075362357	1.08
rs16892766	8q23.3	8	117630683	C	7.35x10-28	0.225967872	1.25
rs6983267	8q24.21	8	128413305	G	7.59x10-39	0.156647548	1.17
rs1412834	9p21.3	9	22110131	T	4.56x10-15	0.093182893	1.10
rs7894531	10p14	10	8734761	G	2.91x10-21	0.1225597	1.13
rs704017	10q22.3	10	80819132	G	1.13x10-14	0.102074303	1.11
rs2193352	10q24.2	10	101346609	G	2.4x10-13	0.109003814	1.12
rs57796856	11q13.4	11	74338355	T	6.25x10-13	0.086000801	1.09
rs4944940	11q13.4	11	74415252	G	2.49x10-16	0.261710362	1.30
rs3087967	11q23.1	11	111156836	T	9.41x10-28	0.141866065	1.15
rs10849438	12p13.31	12	6412036	G	2.17x10-8	0.111707504	1.12
rs11169572	12q13.12	12	51216890	C	1.49x10-12	0.086648644	1.09
rs597808	12q24.12	12	111973358	G	1.09x10-12	0.086470461	1.09
rs7315438	12q24.21	12	115891403	T	4.02x10-11	0.080816822	1.08
rs12427600	13q13.3	13	37460648	C	1.71x10-9	0.084433206	1.09
rs45597035	13q22.1	13	73649152	A	1.26x10-8	0.073302126	1.08
rs1330889	13q22.3	13	78609615	C	2.05x10-8	0.102996202	1.11
rs35107139	14q22.2	14	54419106	C	1.11x10-10	0.084891777	1.09
rs16969681	15q13.3	15	32993111	T	1.07x10-20	0.190961426	1.21
rs73376930	15q13.3	15	33012502	G	3.24x10-25	0.151244549	1.16
rs17816465	15q13.3	15	33156386	A	1.38x10-10	0.09697371	1.10
rs7495132	15q26.1	15	91172901	T	7.74x10-9	0.107898606	1.11
rs61336918	16q23.2	16	80007266	A	1.57x10-11	0.091874735	1.10
rs899244	16q24.1	16	86700030	T	8.13x10-9	0.084950266	1.09
rs1078643	17p12	17	10707241	A	8.25x10-9	0.089463518	1.09
rs7226855	18q21.1	18	46454048	A	2.44x10-57	0.193705878	1.21
rs73039434	19q13.11	19	33524919	T	5.9x10-15	0.263650803	1.30
rs12979278	19q13.33	19	49218602	T	8.18x10-9	0.071678575	1.07
rs961253	20p12.3	20	6404281	A	4.23x10-17	0.103634512	1.11
rs994308	20p12.3	20	6603622	C	2.55x10-10	0.077897577	1.08
rs6085661	20p12.3	20	6693128	T	1.45x10-12	0.086373253	1.09
rs6066825	20q13.13	20	47340117	A	7.02x10-12	0.087063514	1.09
rs4811050	20q13.13	20	48980670	A	1.63x10-10	0.099815626	1.10
rs6091213	20q13.13	20	49384745	C	3.75x10-8	0.077514504	1.08
rs1741640	20q13.33	20	60932414	C	5.38x10-25	0.161518862	1.18

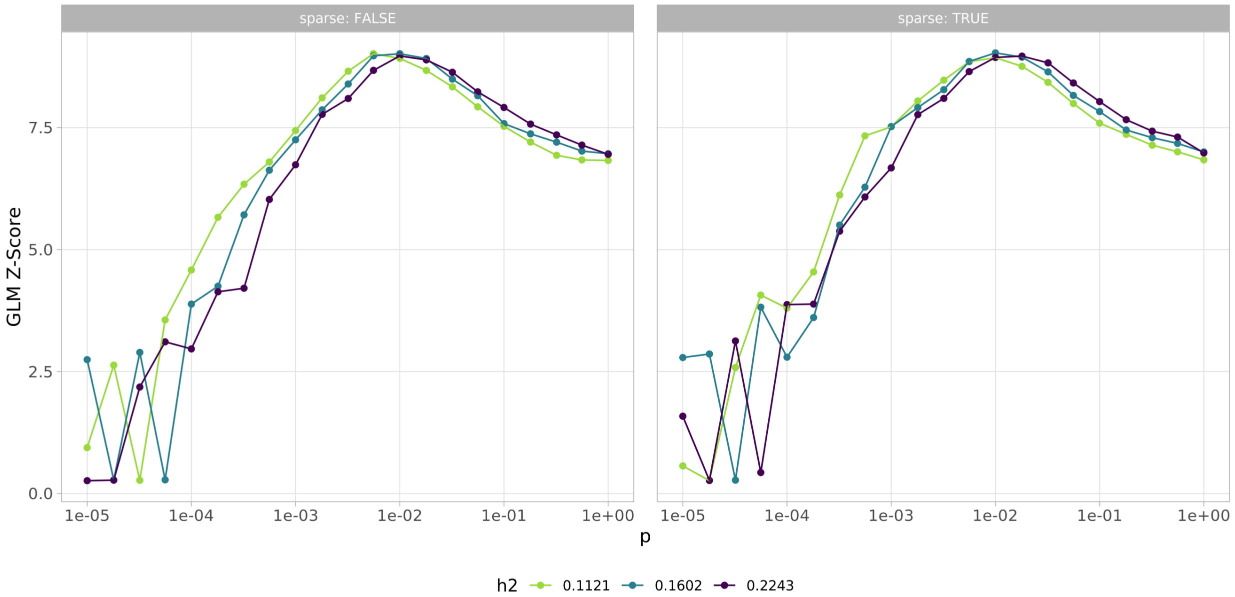

Figure S2. Z-scores for LDpred2-grid PRS calculated across a grid of tuning parameters: estimated heritability (h2), proportion of causal variants, p, and sparsity (true or false) (after Privé *et al.*) For the top performing non-sparse grid PRS, the proportion of causal variants was 0.0056, and heritability of 0.1121; for the top-performing sparse model, proportion of causal variants was 0.01, and heritability 0.1602, with sparsity 0.44137.

Evaluation of polygenic risk score performance

Each PRS was evaluated in logistic regression and Cox models, adjusting for age, sex, array and 4 principal components. Age, sex and PCs were all modelled as continuous variables, assuming a linear relationship. For Cox models we confirmed proportional hazards assumptions held through visual inspection of plots of Schoenfeld residuals. We evaluated potential interactions between PRS and age by examining the prognostic strength and significance of interaction terms based on Wald χ^2statistics, and plotting marginal effects of PRS with age. We compared model performance to a reference model, containing age, sex, array and 4 principal components, to assess the contribution of the PRS to model performance. Further models were also derived which did not adjust for age and sex,[10] to evaluate the contribution which these factors (known to be independent predictors for CRC risk) made to the performance of the full model.

In order to compare PRS distributions for each cohort, and effect sizes per SD of each PRS, we standardised the PRS to have a mean of 0 and standard deviation of 1 in the Test Cohort. We also used these standardised scores in plots of marginal effects of PRS in interaction with age. Remaining analyses used non-standardised scores.

Validation of QCancer-10

Validation of QCancer-10 in UKB permits evaluation of model performance in a population of approximately bowel screening age. Full QCancer-10 model specification is available at https://www.qcancer.org/15yr/colorectal/.[11]

CRC outcomes were identified as described in the main paper. Of note, in QCancer-10 (colorectal cancer) development ICD-10 codes for anal cancer were included in case definition. We did not include these in this study, as anal cancers are of a different aetiology to CRC, and bowel cancer screening does not aim to detect these lesions. Previous medical history, alcohol and smoking status, and family history were all taken from self-reported data in baseline touch-screen and verbal UKB assessment centre interviews.

Mapping of ethnicity, smoking and alcohol intake is given in Table S2. Ethnicity was coded from self-reported ethnicity (UKB field 21000). Smoking history was compiled from the smoking summary field (field 20116), frequency of smoking (field 1239) and number of cigarettes smoked (field 3456). To calculate alcohol intake, reported alcohol intake frequency (field 1558) was combined with detailed drink-based intake reported in

glasses/pints at touchscreen interview. Drinks intake was converted to units using NHS Choices Livewell alcohol units (as in Usher-Smith *et al.*[12]), and average daily units calculated.

Previous medical history was taken from self-reported cancer and non-cancer illnesses (fields 20001 and 20002) at touch-screen interview (Table S3).

Family history in UKB is for first degree relatives, detailed for father, mother and siblings separately; we considered positive family history to be CRC in any of these relatives. In QCancer-10 development, absence of data carries the assumption that the individual does not have any family history; family history was therefore coded as missing only if the answer for all of these was either 'Do not know' or 'Prefer not to answer'.

Distributions of continuous predictors were evaluated. One implausible value for BMI was set to missing and otherwise all values were retained. Of note there are a very small number of UKB participants aged 38-39 and 71-73 years at baseline assessment, who were included in our modelling.

Table S2: Mapping of UK Biobank ethnicity, smoking and alcohol data to QCancer-10 coding

QCancer-10 Coding	UK Biobank Coding
**Ethnicity**	
White/not recorded	White, British, Irish, Any other white background, Prefer not to answer, Do not know, Missing
Indian	Indian
Pakistani	Pakistani
Bangladeshi	Bangladeshi
Other Asian	Asian or Asian British, Any other Asian background
Caribbean	Caribbean
Black African	African
Chinese	Chinese
Other	Black or Black British, Any other Black background, Mixed, White and Black Caribbean, White and Black African, White and Asian, Any other mixed background, Other ethnic group
**Smoking**	
Non-smoker	Smoking summary = 'Never'
Ex-smoker	Smoking summary = 'Previous'
Light smoker	Smoking summary = 'Current' AND Cigarettes < 10 OR frequency = 'Only occasionally'
Moderate smoker	Smoking summary = 'Current' AND Cigarettes = 10-19
Heavy smoker	Smoking summary = 'Current' AND Cigarettes >20
Missing	Smoking summary = 'Missing'/ 'Prefer not to answer'
**Alcohol**	
Non-drinker	Alcohol frequency = 'Never'
Trivial drinker	<1 calculated daily unit
Light drinker	1-2 calculated daily units
Moderate drinker	3-6 calculated daily units
Heavy drinker	7-9 calculated daily units
Very heavy drinker	10 or more calculated daily units
Missing	Alcohol frequency = 'Missing'/ 'Prefer not to answer'

Table S3: UK Biobank codes self-reported medical history for QCancer-10 predictors

Medical condition	UK Biobank codes
Diabetes	1223, 1220
Ulcerative colitis	1463
Bowel polyps	1460
Breast cancer	1002
Uterine cancer	1040
Ovarian cancer	1039
Cervical cancer	1041
Lung cancer	1001
Blood cancers	1047, 1048, 1050, 1051, 1052, 1053, 1055, 1056, 1058
Oral cancers	1004, 1005, 1006, 1010, 1011, 1012, 1015, 1077, 1078, 1079

Integrated model development

Riley *et al.* propose minimum sample size requirements for developing new prediction models which go beyond the historically recommended 20 events per variable, implemented in R package pmsampsize.[13] This uses the anticipated Cox-Snell R^2, number of predictors considered in the model, duration of follow-up, and expected event rate to calculate sample size and number of cases required.

We derived the Cox-Snell R^2 as described by Riley *et al.*[13] from the C-statistics from the open cohort of QCancer-10 validation performed in UK Biobank by Usher-Smith *et al.*[12] (0.70 and 0.65 for male and female models respectively), and mean follow-up and CRC rates calculated for individuals available for the Integrated Modelling Cohort. The number of predictors included in the integrated (genetic + non-genetic) model for each sex was calculated as follows for QCancer-10 risk score components: 1 for each degree of freedom of each categorical variable (alcohol intake = 5; ethnicity = 8; smoking = 4); 1 each for continuous variables (BMI, Townsend Deprivation Score); 1 for each boolean predictor; 1 parameter for each fractional polynomial term for age; and 2 parameters for each interaction term calculated; 1 for the QCancer-10 risk score itself. With 1 additional parameter added for the PRS, this totalled 34 parameters for men and 33 for women.

Sample size calculations indicated that for the integrated male model, 27.43 events per candidate predictor parameter (EPP) are needed, giving a minimum sample size of 94996 and 933 events. As a result of lower CRC incidence and expected model performance in women, the EPP required was 47.53, minimum sample size 253780, with 1569 events. Whilst the numbers required for the male model are readily achievable, the sample size and cases available for the female model fall slightly short in the our available Integrated Modelling Cohort (n = 238496, including 1458 cases). Whilst we continued with model development, for the female integrated models the estimate of outcome risk may be less precise, and the model may be more subject to over-fitting.[14] External validation of the integrated model will be essential prior to implementation.

We constructed Cox models in the Integrated Modelling Cohort including two predictors: the risk score from QCancer-10 and a PRS. PRS were adjusted for genotyping array and the first four principal components from UK Biobank study. We developed male and female models separately, and compared the use of the top-performing genome-wide PRS, and the GWAS-sig PRS. We truncated the lower and upper 0.5% of the distributions of each predictor to the outer bounds.[15] Inspection of Schoenfeld residuals showed that the proportional hazard assumption held. We evaluated the use of multiple fractional polynomials to model the predictors. We assessed possible interactions between the predictors by visual inspection of plots of marginal effects of the QCancer-10 risk score across PRS values, and examining the prognostic strength and significance of interaction terms based on Wald χ^2 statistics.

Decision Curve Analysis

We used decision curve analysis (DCA) to evaluate the potential impact of our models on clinical decision making.[16,17] We assumed the decision in question was whether an individual in the general population ought to undergo screening colonoscopy, based on their risk. The outcome of DCA, net benefit (NB), was calculated as the number of true positives minus the number of false positives (i.e. unnecessary procedures), with an

"exchange rate" applied to false positives by weighting them by the odds at the given risk threshold.[18] As given in Vickers *et al.*,[16]

$$NB = \frac{True\ Positives}{N} - \frac{False\ Positives}{N}\left(\frac{p_t}{1 - p_t}\right)$$

where p_t is the probability threshold and N is the sample size.

For a survival model, survival data is converted to a binary outcome at a given timepoint; here we used 8 years of follow-up. Where an individual's probability of disease using the prediction model is ≥ p_t, x = 1, otherwise x = 0. Then, the number of true positives is [1 - (S_t| x = 1)] × P(x = 1) × *N*, and the number of false positives is (S_t | x = 1) × P(x = 1) × *N*, where S_t is the Kaplan Meier probability at the time point in question.[19]

One can also evaluate NB in terms of true negatives rather than true positives, which equates to the number of unnecessary interventions avoided in using a risk model. We used the R function dca::stdca to calculate NB and unnecessary interventions avoided at 8 years of follow-up, and plotted these across the full range of thresholds in which the models provided benefit.

Overall, the model with the highest NB is considered the "best" strategy in DCA. However this evaluation does not incorporate the added implications of undertaking PRS. Whether the additional burden is worthwhile can be evaluated by calculating the test trade-off. We calculated the increase in net benefit (ΔNB) at pre-specified thresholds (see below) afforded by adding the LDpred2 PRS to the QCancer-10 model, and used this to calculated the test trade-off, which is 1/ΔNB.[18] This indicates the number of additional tests (here PRS) which would be needed in order to obtain one more true positive CRC diagnosis using the model. Future analyses to measure and evaluate financial costs of risk score tests, environmental impact,[20] and potential effects on screening participation would be required to investigate this issue in detail.

Vickers *et al.* note that the probability threshold may also be considered as the number needed to test to identify a cancer,[21] which here would be the number needed to screen. In order to identify relevant thresholds in which to evaluate NB and test trade-off, we searched the existing literature to identify potentially relevant thresholds which have been deemed acceptable in clinical practice. In randomised trials of colonoscopy based screening, the NNS for CRC was 1000 in two studies based in Spain and the USA,[22,23] 182 in a Dutch colonoscopy trial,[24] and 202 in a multi-country European study,[25] equating to probability thresholds of 0.1-0.5%. Of note, however, these thresholds reflect immediate risk of CRC, rather than longer term risk (e.g. 8-year risk in our DCA).

The threshold probability considered is context/patient specific and is the level at which one feels equivocally about the benefits and harms of the intervention.[26] In a population-based screening programme, concern around cost of colonoscopy (in terms of financial and opportunity costs due to capacity) will be greater than in a randomised trial, resulting in a higher threshold probability. In informing individual patient choice, patient and clinician preference around risk may vary considerably depending on their level of concern around cancer and the colonoscopy procedure. We therefore evaluated thresholds of 0.5%, 1%, 1.5% and 2% in our calculations, to provide a range of potentially relevant values for policy makers, clinicians and screening participants.

We note also that a significant portion of the benefit of colonoscopy screening arises from preventing neoplasia through the removal of advanced adenomas (AA) at colonoscopy, which is not reflected in these numbers, and could not be readily evaluated in this study due to lack of sufficiently high resolution data on colorectal polyps in UKB. For all advanced neoplasia (i.e. AAs and CRC), NNS in the above trials ranged from 9-49, equating to probability thresholds of 2-10% and reflecting the higher prevalence of AA compared to CRC.

Software

R package bigsnpr v1.5.2[27] was used for genome-wide PRS development, epitools v 0.5-10.1,[2] rms v5.1-4,[28] mfp v1.5 .2,[29] and survival v3.1-8[3] for modelling, and packages from the tidyverse suite[30] for data analysis and presentation.

Supplementary Results

Table S4: Characteristics and missingness of predictor values for the whole UKB cohort, excluding individuals with prevalent CRC. Values are numbers (%) unless otherwise indicated. CRC – colorectal cancer, IQR – interquartile range, NA – not applicable. *not included in model for females but provided for information.

	Male	Female
Age (years), median (IQR)	58.0 (14.0)	57.0 (13.0)
Ethnicity		
White/not recorded	215121 (94.6)	257402 (94.6)
Indian	3003 (1.3)	2933 (1.1)
Pakistani	1118 (0.5)	716 (0.3)
Bangladeshi	159 (0.1)	74 (0.0)
Other Asian	996 (0.4)	857 (0.3)
Caribbean	1637 (0.7)	2855 (1.0)
Black African	1701 (0.7)	1677 (0.6)
Chinese	581 (0.3)	989 (0.4)
Other	3107 (1.4)	4529 (1.7)
Follow-up (years), median (IQR)	7.08 (1.34)	7.09 (1.31)
< 5 years	405 (0.2)	490 (0.2)
Townsend deprivation index, median (IQR)	-2.1 (4.3)	-2.1 (4.1)*
Missing	293 (0.1)	325 (0.1)
BMI (kg/m2), median (IQR)	27.3 (5.1)	26.1 (6.3)*
Missing	1639 (0.7)	1448 (0.5)
Smoking status		
Non-smoker	110840 (48.7)	161336 (59.3)
Ex-smoker	86721 (38.1)	84928 (31.2)
Light smoker	11009 (4.8)	10158 (3.7)
Moderate smoker	6958 (3.1)	8402 (3.1)
Heavy smoker	10474 (4.6)	5708 (2.1)
Missing	1421 (0.6)	1500 (0.6)
Alcohol intake		
Non-drinker	14472 (6.4)	25889 (9.5)
Trivial drinker	48955 (21.5)	109497 (40.3)
Light drinker	66332 (29.2)	87095 (32)
Moderate drinker	69467 (30.5)	42795 (15.7)
Heavy drinker	17115 (7.5)	4374 (1.6)
Very heavy drinker	10323 (4.5)	1646 (0.6)
Missing	759 (0.3)	736 (0.3)
Family history of CRC	21638 (9.5)	24773 (9.1)
Missing	9652 (4.2)	7262 (2.7)
Diabetes	15513 (6.8)	9294 (3.4)
Missing	358 (0.2)	298 (0.1)
Colorectal polyps	711 (0.3)	708 (0.3)
Missing	378 (0.2)	310 (0.1)
Ulcerative colitis	1187 (0.5)	1379 (0.5)
Missing	378 (0.2)	310 (0.1)
Breast cancer	NA	11165 (4.1)
Missing	NA	649 (0.2)

Uterine cancer	NA	1194 (0.4)
Missing	NA	649 (0.2)
Ovarian cancer	NA	811 (0.3)
Missing	NA	649 (0.2)
Cervical cancer	NA	1985 (0.7)
Missing	NA	649 (0.2)
Lung cancer	149 (0.1)	NA
Missing	631 (0.3)	NA
Blood cancer	1356 (0.6)	NA
Missing	631 (0.3)	NA
Oral cancer	576 (0.3)	NA
Missing	631 (0.3)	NA
Imputed genetic data passing standard QC	220923 (97.1)	262403 (96.5)
Missing	6500 (2.9)	9629 (3.5)

Table S5: Demographics of derivation and validation cohorts used in PRS development (logistic regression modelling cohorts)

	Derivation Training		Derivation Test		Geographic Validation		Minority Ethnic Validation	
	**Controls (n = 29554)**	**Cases (n = 446)**	**Controls (n = 276436)**	**Cases (n = 4230)**	**Controls (n = 33541)**	**Cases (n = 611)**	**Controls (n = 27248)**	**Cases (n = 255)**
Male (n, %)	13751 (46.5)	254 (57.0)	127823 (46.2)	2425 (57.3)	14851 (44.3)	330 (54.0)	12746 (46.8)	128 (50.2)
Female (n, %)	15803 (53.5)	192 (43.0)	148611 (53.8)	1805 (42.7)	18690 (55.7)	281 (46.0)	14502 (53.2)	127 (49.8)
Age (mean, SD)	56.82 (8.01)	61.64 (6.10)	56.84 (7.99)	61.41 (6.15)	56.31 (8.05)	61.00 (6.51)	52.75 (8.25)	58.25 (7.97)
Age (min-max)	40-70	40-70	39-72	40-70	40-70	40-70	39-72	40-70

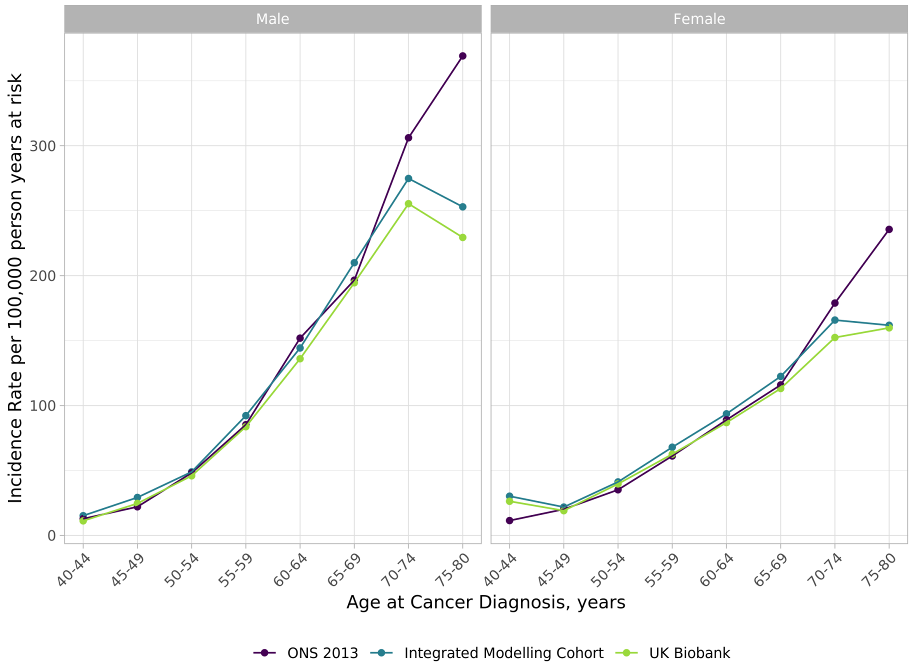

Figure S3. Age specific CRC rates in men and women in the UK Biobank cohort overall and Integrated Modelling cohorts, compared to Office for National Statistics 2013 Cancer Registry data.[31] Cases for the whole UK Biobank cohort are from linked cancer registry data; cases for the Integrated Modelling Cohort are from linked cancer registry, death registry, and hospital data.

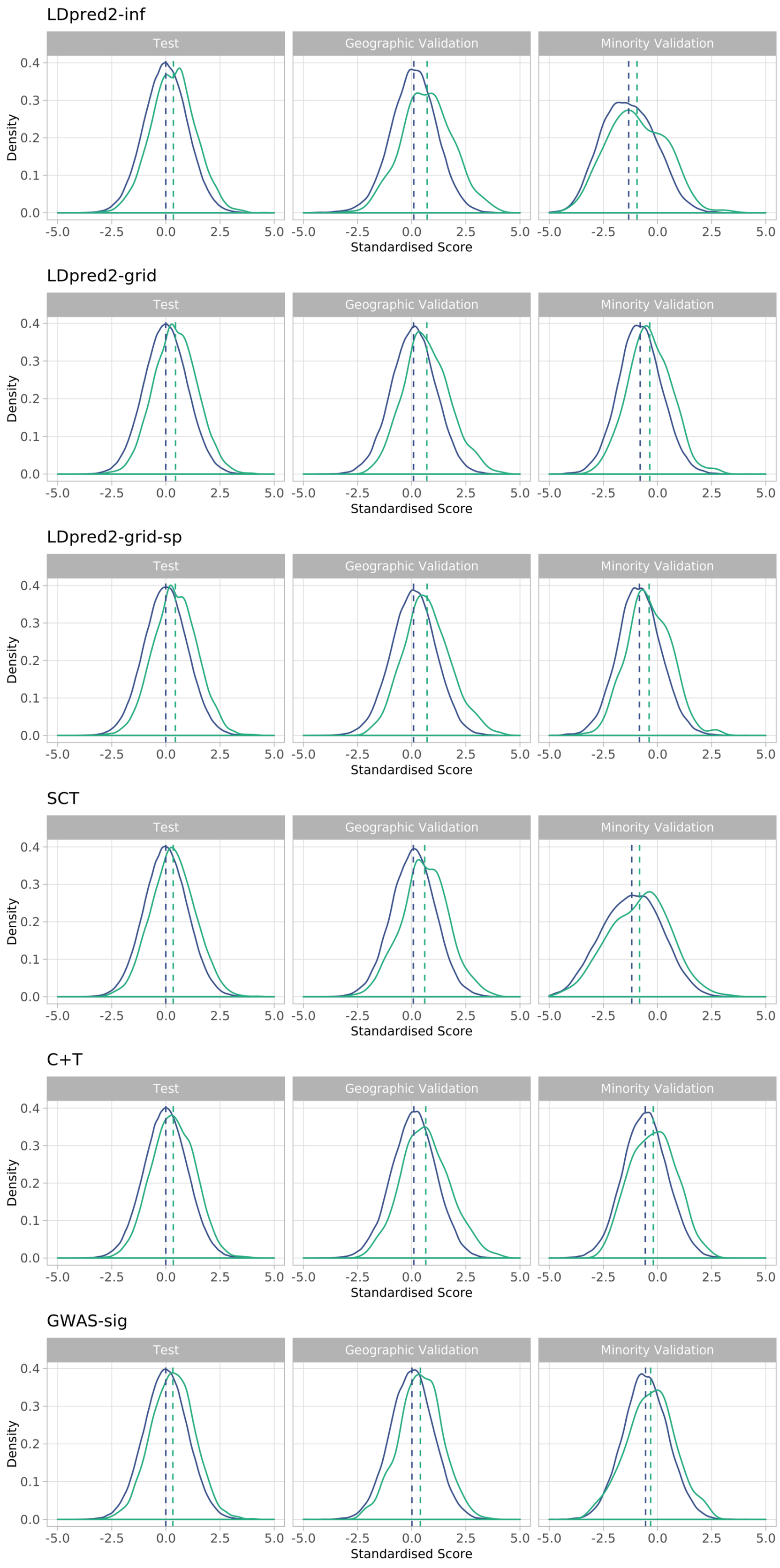

Figure S4. Distributions of standardised PRS for PRS Test Cohort and Validation Cohorts. Case distribution is shown in green, controls in blue.

Interactions between PRS and Age

Evaluation of interaction terms (Table S6) indicated a significant interaction between age and PRS (at p < 0.01) for the LDpred-inf model only in logistic regression models, and for LDpred2-grid, LDpred2-grid-sp and C+T Cox models. Plots of marginal effects (shown for logistic regression models in Figure S5) indicated a reduction in effect of PRS with increasing age. Plots for Cox models were similar. Given the weakness of the interaction terms relative to the other predictors based on Wald χ^2, we elected not to include interaction terms in the models.

Table S6. Wald χ^2 of interaction terms between PRS and age in logistic regression and Cox models

	χ2 (p value)		
	PRS	age	PRS * age
**Logistic regression**			
LDpred2-inf	529 (<0.001)	1254 (<0.001)	8 (0.004)
LDpred2-grid	860 (<0.001)	1254 (<0.001)	3 (0.065)
LDpred2-grid-sp	829 (<0.001)	1254 (<0.001)	3 (0.068)
SCT	500 (<0.001)	1254 (<0.001)	2 (0.136)
C+T	509 (<0.001)	1252 (<0.001)	3 (0.064)
GWAS-sig	447 (<0.001)	1248 (<0.001)	1 (0.457)
**Cox regression**			
LDpred2-inf	207 (<0.001)	575 (<0.001)	4 (0.038)
LDpred2-grid	428 (<0.001)	578 (<0.001)	9 (0.003)
LDpred2-grid-sp	405 (<0.001)	577 (<0.001)	8 (0.005)
SCT	222 (<0.001)	576 (<0.001)	4 (0.035)
C+T	242 (<0.001)	576 (<0.001)	9 (0.003)
GWAS-sig	225 (<0.001)	574 (<0.001)	7 (0.011)

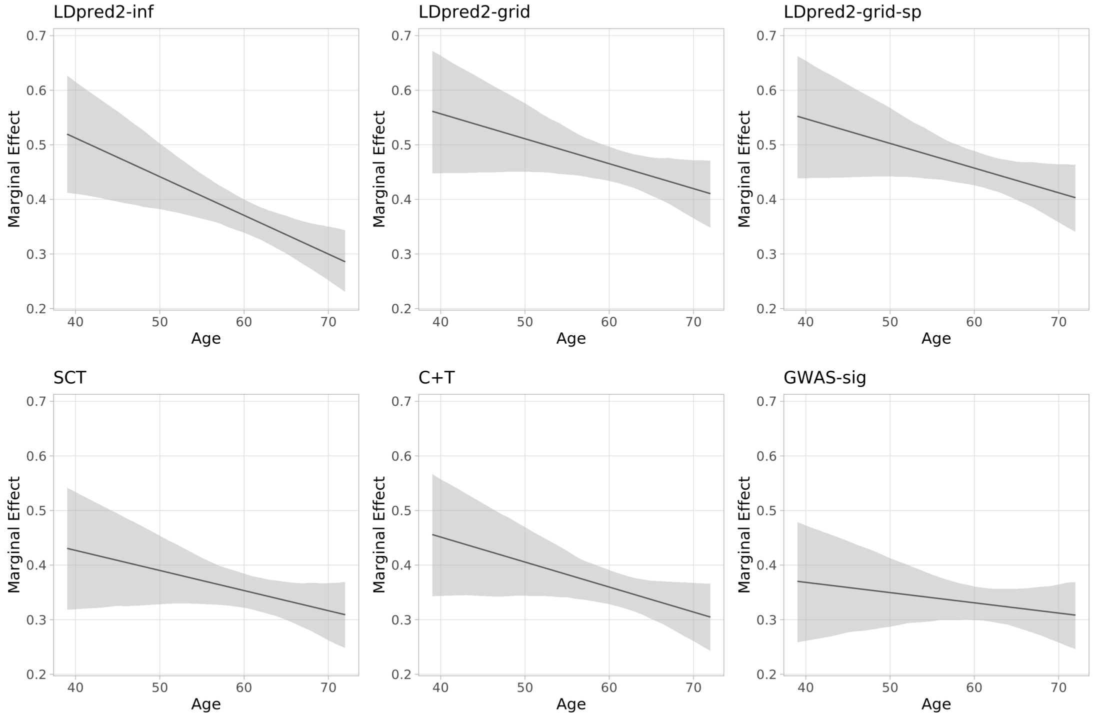

Figure S5. Marginal effect of standardised PRS in interaction with age in linear regression models.

Table S7. Apparent performance of PRS assessed in logistic regression models in the Test Cohort, with and without adjustment for sex and age

	LDpred2-inf	LDpred2-grid	LDpred2-grid-sp	SCT	C+T	GWAS-sig
**With sex and age**						
C	0.704 (0.697 - 0.712)	0.717 (0.711 - 0.725)	0.716 (0.710 - 0.723)	0.702 (0.695 - 0.711)	0.704 (0.697 - 0.711)	0.700 (0.693 - 0.707)
Dxy	0.407 (0.394 - 0.423)	0.435 (0.422 - 0.451)	0.432 (0.419 - 0.446)	0.404 (0.389 - 0.422)	0.407 (0.394 - 0.423)	0.400 (0.386 - 0.414)
R2 (%)	5.5 (5.1 - 5.9)	6.3 (5.9 - 6.8)	6.2 (5.8 - 6.7)	5.4 (5.0 - 5.9)	5.4 (5.1 - 5.9)	5.3 (4.9 - 5.7)
Scaled Brier (%)	0.87	1.05	1.03	0.86	0.85	0.83
**Without sex and age**						
C	0.597 (0.589 - 0.606)	0.626 (0.618 - 0.634)	0.623 (0.614 - 0.632)	0.594 (0.587 - 0.603)	0.597 (0.589 - 0.606)	0.592 (0.584 - 0.601)
Dxy	0.194 (0.178 - 0.212)	0.251 (0.235 - 0.268)	0.247 (0.229 - 0.264)	0.189 (0.175 - 0.206)	0.193 (0.178 - 0.211)	0.185 (0.169 - 0.202)
R2 (%)	1.3 (1.1 - 1.5)	2.1 (1.8 - 2.4)	2.0 (1.8 - 2.3)	1.2 (1.0 - 1.5)	1.3 (1.1 - 1.5)	1.1 (0.9 - 1.3)
Scaled Brier (%)	0.21	0.34	0.33	0.19	0.19	0.17

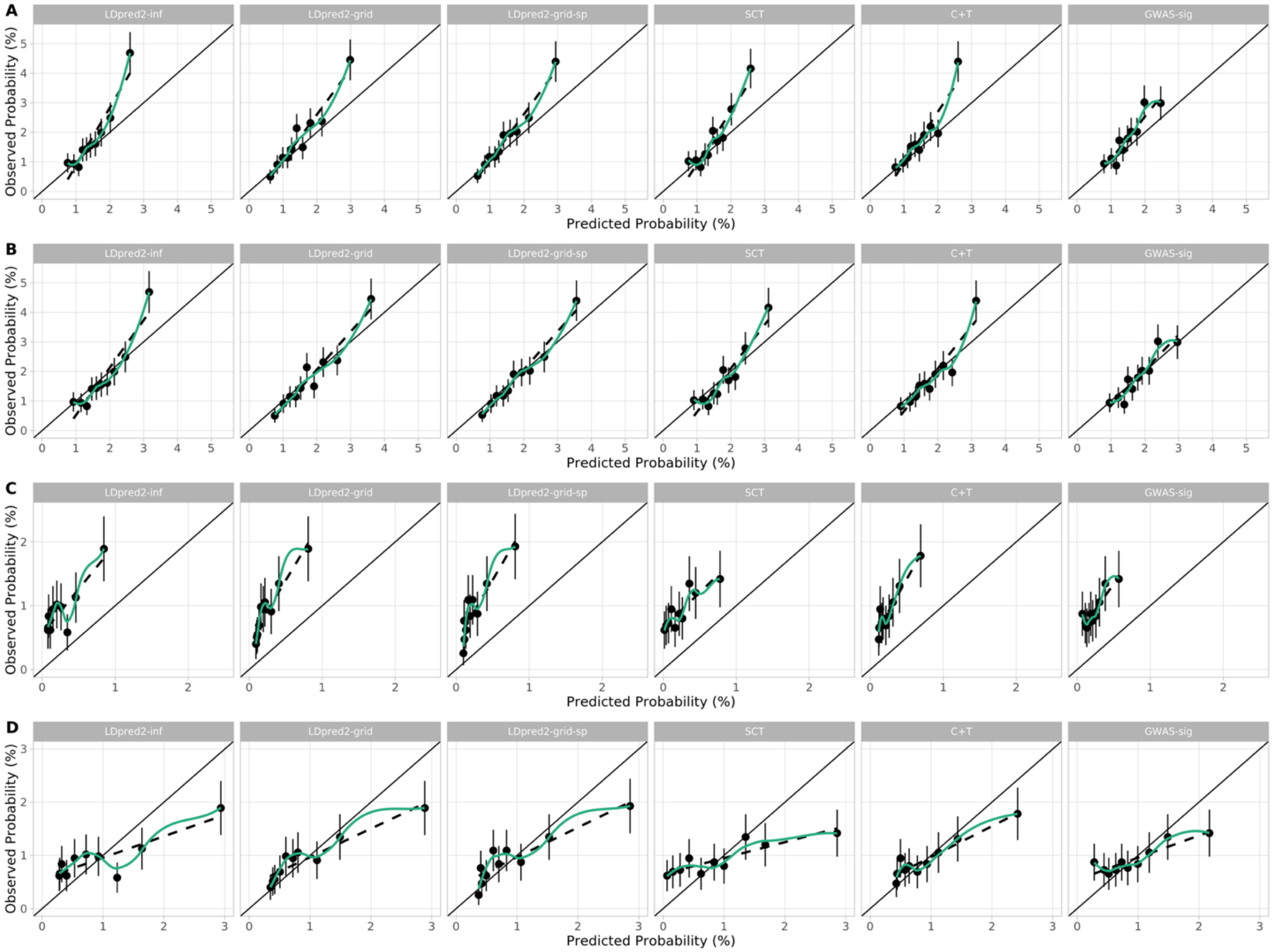

Figure S6. Calibration plots of PRS models in logistic regression analyses in Validation Cohorts. Panels show calibration for the Geographic Validation Cohort before (A) and after (B) recalibration, and in the Minority Ethnic Validation Cohort before (C) and after (D) recalibration. Plots show predicted and observed probability of CRC by tenths of PRS for each model.

Table S8. Subgroup analysis of PRS logistic regression model performance by sex and in individuals with a first degree family history of CRC in the Geographic Validation Cohort.

	LDpred2-inf	LDpred2-grid	LDpred2-grid-sp	SCT	C+T	GWAS-sig
**Males**						
C	0.731 (0.705 - 0.760)	0.740 (0.716 - 0.767)	0.741 (0.715 - 0.768)	0.728 (0.702 - 0.753)	0.726 (0.702 - 0.755)	0.716 (0.689 - 0.743)
Dxy	0.463 (0.410 - 0.519)	0.481 (0.433 - 0.534)	0.481 (0.431 - 0.536)	0.455 (0.404 - 0.507)	0.453 (0.403 - 0.510)	0.433 (0.378 - 0.486)
R2 (%)	7.6 (6.0 - 9.3)	8.3 (6.6 - 10.1)	8.3 (6.6 - 10.1)	7.2 (5.5 - 8.7)	7.2 (5.6 - 8.9)	6.6 (5.0 - 8.2)
Slope	1.216 (1.047 - 1.409)	1.171 (1.025 - 1.343)	1.182 (1.034 - 1.357)	1.178 (1.006 - 1.354)	1.187 (1.026 - 1.371)	1.137 (0.968 - 1.320)
CITL	0.186 (0.075 - 0.287)	0.178 (0.068 - 0.279)	0.180 (0.070 - 0.281)	0.174 (0.067 - 0.275)	0.176 (0.066 - 0.278)	0.170 (0.061 - 0.273)
Scaled Brier (%)	1.73	1.90	1.90	1.47	1.60	1.37
**Females**						
C	0.709 (0.680 - 0.739)	0.712 (0.682 - 0.741)	0.714 (0.684 - 0.743)	0.694 (0.666 - 0.723)	0.699 (0.669 - 0.731)	0.673 (0.643 - 0.703)
Dxy	0.419 (0.360 - 0.477)	0.423 (0.365 - 0.481)	0.427 (0.368 - 0.486)	0.387 (0.331 - 0.447)	0.397 (0.338 - 0.462)	0.346 (0.287 - 0.406)
R2 (%)	5.7 (4.0 - 7.4)	6.0 (4.1 - 7.7)	6.1 (4.3 - 7.8)	4.8 (3.2 - 6.6)	5.2 (3.3 - 6.9)	3.4 (1.8 - 5.1)
Slope	1.102 (0.919 - 1.292)	1.035 (0.881 - 1.196)	1.055 (0.896 - 1.214)	1.002 (0.839 - 1.185)	1.041 (0.863 - 1.236)	0.862 (0.700 - 1.036)
CITL	0.230 (0.111 - 0.352)	0.221 (0.100 - 0.343)	0.223 (0.101 - 0.344)	0.217 (0.098 - 0.339)	0.218 (0.098 - 0.340)	0.215 (0.097 - 0.342)
Scaled Brier (%)	1.04	1.20	1.22	0.90	0.98	0.59
**First degree family history**						
C	0.697 (0.637 - 0.748)	0.701 (0.642 - 0.754)	0.706 (0.647 - 0.758)	0.685 (0.625 - 0.738)	0.703 (0.646 - 0.752)	0.668 (0.608 - 0.721)
Dxy	0.394 (0.275 - 0.496)	0.402 (0.283 - 0.509)	0.412 (0.293 - 0.515)	0.369 (0.251 - 0.475)	0.406 (0.292 - 0.504)	0.335 (0.217 - 0.443)
R2 (%)	2.4 (-2.2 - 6.0)	3.4 (-1.1 - 7.4)	3.6 (-0.9 - 7.5)	1.8 (-2.8 - 5.8)	2.9 (-1.7 - 6.6)	0.5 (-4.1 - 4.6)
Slope	1.021 (0.714 - 1.322)	0.971 (0.683 - 1.259)	0.997 (0.714 - 1.286)	0.948 (0.633 - 1.246)	1.052 (0.745 - 1.363)	0.838 (0.518 - 1.145)
CITL	0.658 (0.462 - 0.827)	0.607 (0.409 - 0.771)	0.613 (0.414 - 0.776)	0.643 (0.451 - 0.812)	0.644 (0.453 - 0.810)	0.633 (0.443 - 0.809)
Scaled Brier (%)	1.07	1.45	1.45	1.03	1.14	0.77

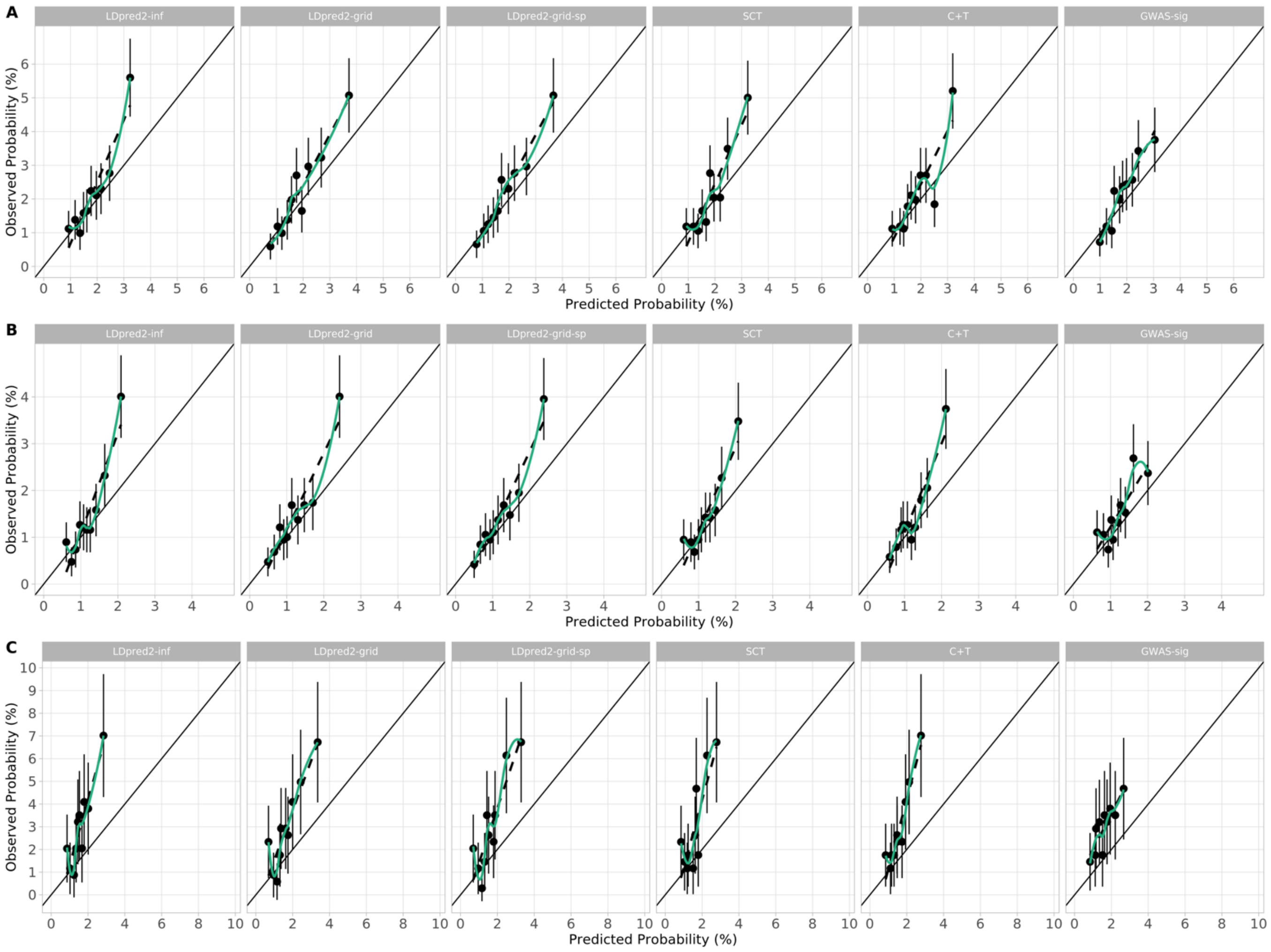

Figure S7. Calibration of PRS in logistic regression models in subgroup analysis in the Geographic Validation Cohort. Plots show predicted and observed probability of CRC by tenths of PRS for each model in males (A), females (B), and those with a first degree family history of CRC (C).

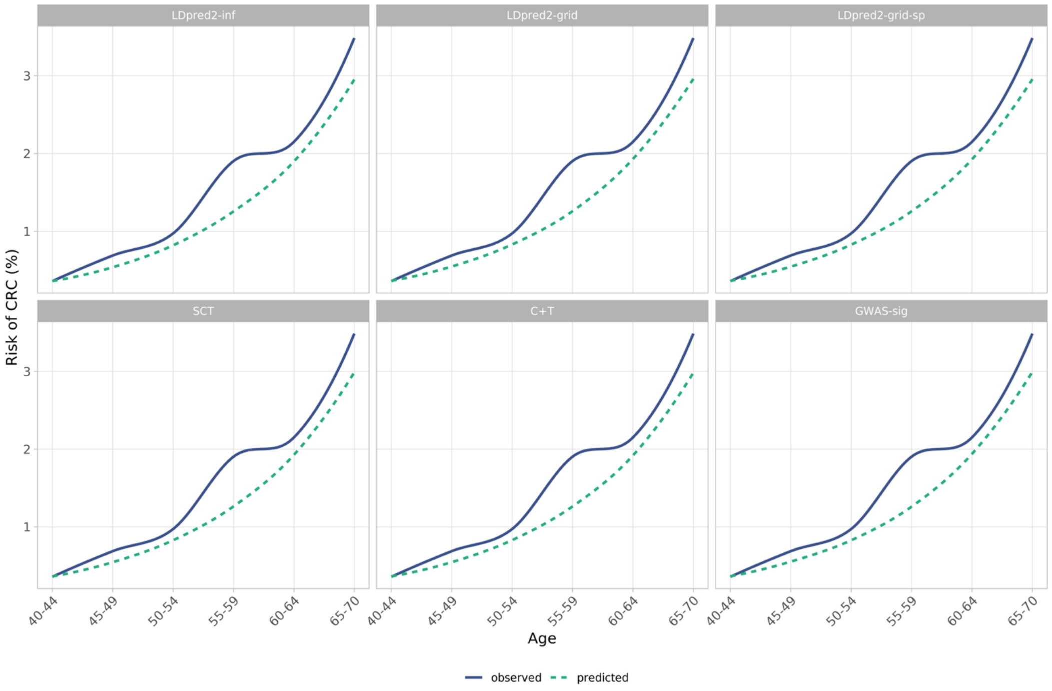

Figure S8. Observed and predicted risk of CRC for PRS logistic regression models across 5 year age bands (in the Geographic Validation Cohort).

Table S9. Apparent, internally and externally validated polygenic risk score (PRS) performance in Cox's proportional hazards models (adjusting for age, sex, array and first 4 principal components). Values are performance indices plus 95% confidence intervals are provided for each cohort. PRS HR per SD – adjusted hazard ratio of PRS in model per standard deviation of the PRS; C – Harrell's C index; Dxy – Somers' Dxy rank correlation; D – Royston's D statistic; R2D – Royston and Sauerbrei's R_D^2 (explained variation); Slope – Calibration Slope. Pairwise comparison of performance metrics in validation cohorts were all significantly different P<0.001 except comparisons marked ^ where P=0.043, * where P=0.009, and ** where P>0.1.

	LDpred2-inf	LDpred2-grid	LDpred2-grid-sp	SCT	C+T	GWAS-sig	Reference
**Apparent performance**							
PRS HR per SD	1.368 (1.310 - 1.428)	1.563 (1.498 - 1.631)	1.545 (1.480 - 1.612)	1.378 (1.321 - 1.438)	1.397 (1.338 - 1.459)	1.377 (1.320 - 1.436)	NA
C	0.696 (0.685 - 0.707)	0.714 (0.704 - 0.726)	0.712 (0.702 - 0.723)	0.695 (0.685 - 0.706)	0.698 (0.689 - 0.709)	0.695 (0.685 - 0.706)	0.675 (0.665 - 0.687)
Dxy	0.391 (0.370 - 0.414)	0.427 (0.409 - 0.451)	0.424 (0.403 - 0.447)	0.391 (0.370 - 0.412)	0.396 (0.378 - 0.417)	0.390 (0.370 - 0.412)	0.350 (0.331 - 0.373)
D	1.085 (1.027 - 1.150)	1.201 (1.143 - 1.268)	1.190 (1.132 - 1.255)	1.096 (1.034 - 1.163)	1.099 (1.043 - 1.162)	1.094 (1.031 - 1.162)	0.961 (0.902 - 1.021)
R2D (%)	22.0 (20.1 - 24.0)	25.6 (23.8 - 27.8)	25.3 (23.4 - 27.3)	22.3 (20.3 - 24.4)	22.4 (20.6 - 24.4)	22.2 (20.2 - 24.4)	18.1 (16.3 - 19.9)
Scaled Brier (%	0.45	0.56	0.55	0.49	0.47	0.50	0.39
**Internal validation**							
C	0.694	0.713	0.711	0.694	0.697	0.694	0.674
Dxy	0.389	0.425	0.422	0.389	0.393	0.387	0.347
D	1.078	1.194	1.183	1.089	1.091	1.088	0.954
R2D	21.7	25.4	25.1	22.1	22.1	22.0	17.8
Slope	0.992	0.994	0.995	0.994	0.992	0.992	0.992
Scaled Brier (%	0.44	0.55	0.54	0.47	0.46	0.49	0.38
**Geographic Validation**							
C	0.715 (0.686 - 0.743)	0.724 (0.696 - 0.751)	0.725 (0.696 - 0.752)	0.713 (0.686 - 0.740)	0.707 (0.681 - 0.734)	0.701 (0.675 - 0.729)	0.673 (0.644 - 0.702)
Dxy	0.430 (0.372 - 0.485)	0.448 (0.391 - 0.501)	0.450 (0.393 - 0.504)	0.426 (0.372 - 0.480)	0.415 (0.361 - 0.468)	0.402 (0.350 - 0.458)	0.345 (0.288 - 0.404)
D	1.243 (1.075 - 1.406)	1.285 (1.124 - 1.448)	1.293 (1.130 - 1.460)	1.184 (1.029 - 1.346)^	1.182 (1.023 - 1.348)^	1.145 (0.992 - 1.319)	0.945 (0.790 - 1.113)
R2D	27.0 (21.7 - 32.1)	28.3 (23.2 - 33.3)	28.5 (23.4 - 33.7)	25.1 (20.2 - 30.2)^	25.1 (20.0 - 30.3)^	23.8 (19.0 - 29.4)	17.6 (13.0 - 22.9)
Slope	1.123 (0.950 - 1.291)	1.058 (0.911 - 1.204)*	1.073 (0.925 - 1.220)**	1.070 (0.919 - 1.234)**	1.054 (0.897 - 1.223)*	1.023 (0.869 - 1.204)	0.947 (0.774 - 1.142)
Scaled Brier (%	0.75	0.76	0.78	0.63	0.61	0.59	0.37
**Minority Ethnic Validation**							
C	0.647 (0.593 - 0.700)**	0.666 (0.610 - 0.720)	0.664 (0.609 - 0.718)	0.650 (0.596 - 0.705)	0.658 (0.606 - 0.710)	0.659 (0.605 - 0.715)	0.647 (0.595 - 0.702)**
Dxy	0.293 (0.185 - 0.399)**	0.331 (0.221 - 0.440)	0.329 (0.219 - 0.437)	0.300 (0.192 - 0.410)	0.316 (0.212 - 0.420)	0.319 (0.210 - 0.430)	0.293 (0.189 - 0.403)**
D	0.931 (0.650 - 1.273)	1.033 (0.736 - 1.374)	1.030 (0.734 - 1.363)	0.940 (0.640 - 1.281)	0.981 (0.682 - 1.320)	0.995 (0.693 - 1.335)	0.889 (0.610 - 1.229)
R2D	17.2 (9.2 - 27.9)	20.3 (11.5 - 31.1)	20.2 (11.4 - 30.7)	17.4 (8.9 - 28.1)	18.7 (10.0 - 29.4)	19.1 (10.3 - 29.9)	15.9 (8.1 - 26.5)
Slope	0.262 (0.161 - 0.397)	0.314 (0.205 - 0.452)	0.318 (0.207 - 0.455)	0.252 (0.154 - 0.384)**	0.297 (0.188 - 0.442)	0.251 (0.151 - 0.389)**	0.232 (0.136 - 0.366)
Scaled Brier (%	0.16	0.26	0.26	0.16	0.21	0.19	0.14

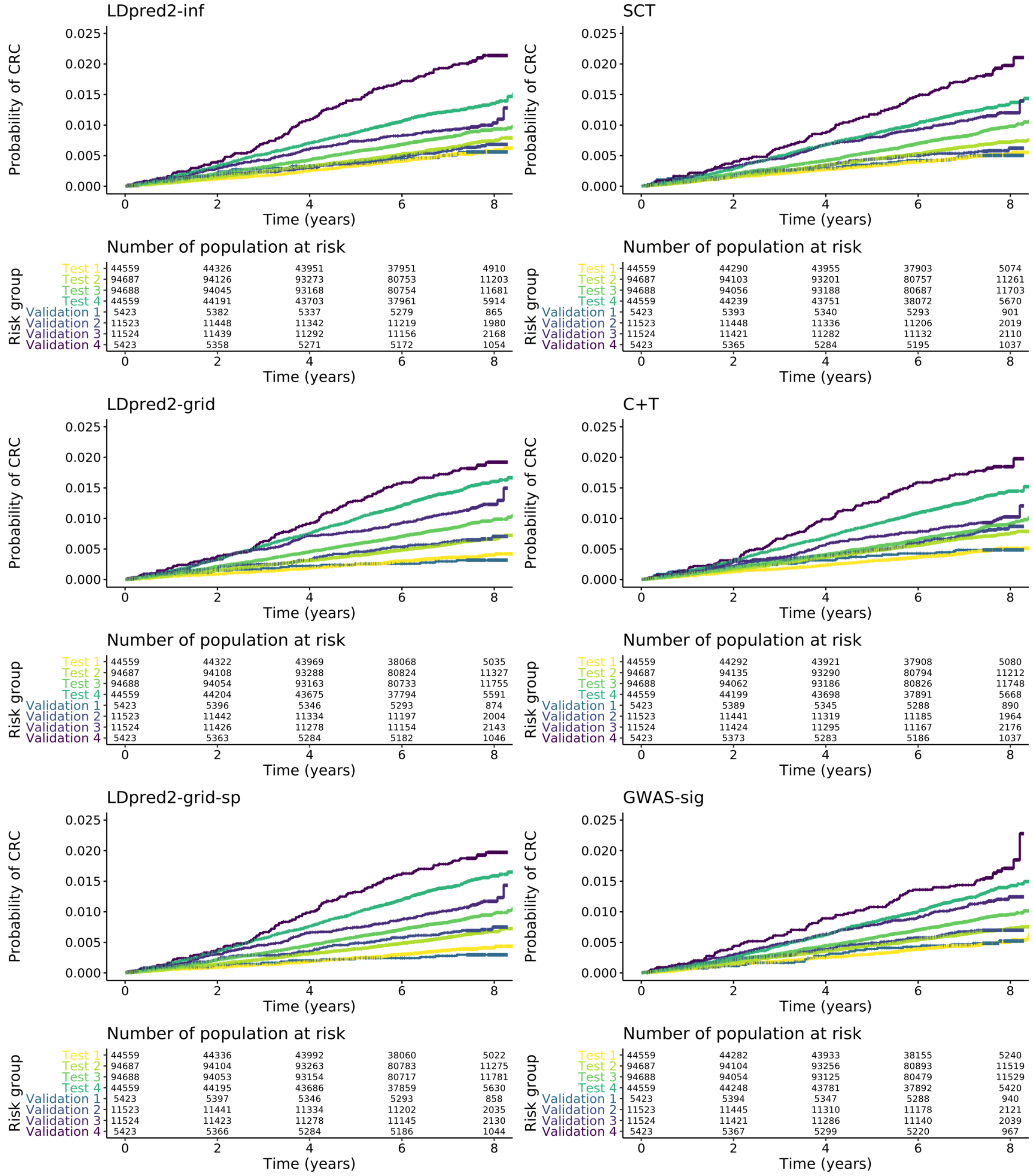

Figure S9. Kaplan-Meier cumulative incidence curves across four risk groups (group 4 being highest risk) for PRS in the Geographic Validation Cohort compared with the Test Cohort

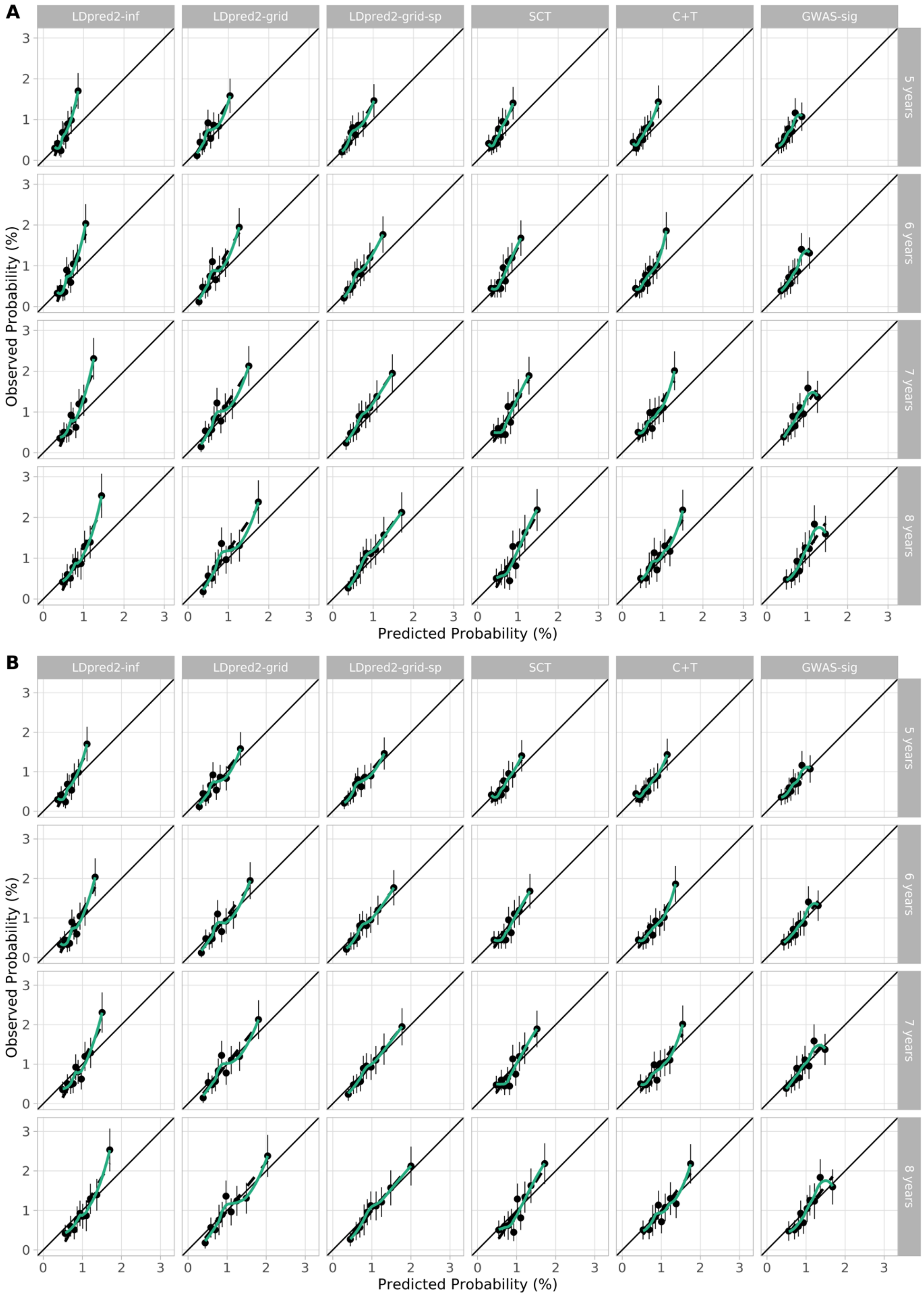

Figure S10. Calibration plots of PRS models in Cox models in the Geographic Validation Cohort. Plots show predicted and observed probability of CRC by tenths of PRS before (A) and after (B) recalibration for each model.

Table S10. Subgroup analysis of PRS Cox model performance by sex in the Geographic Validation Cohort. We did not assess performance specifically in those with a first degree family history, as there were too few incident cases in this group.

	LDpred2-inf	LDpred2-grid	LDpred2-grid-sp	SCT	C+T	GWAS-sig
**Males**						
C	0.709 (0.675 - 0.747)	0.724 (0.691 - 0.761)	0.723 (0.691 - 0.760)	0.711 (0.677 - 0.745)	0.704 (0.668 - 0.740)	0.707 (0.675 - 0.740)
Dxy	0.419 (0.349 - 0.493)	0.448 (0.382 - 0.522)	0.446 (0.382 - 0.520)	0.422 (0.354 - 0.489)	0.408 (0.337 - 0.481)	0.414 (0.350 - 0.481)
D	1.197 (0.989 - 1.430)	1.272 (1.072 - 1.486)	1.271 (1.063 - 1.499)	1.149 (0.963 - 1.359)	1.156 (0.938 - 1.383)	1.185 (0.991 - 1.394)
R2D (%)	25.5 (18.9 - 32.8)	27.9 (21.5 - 34.5)	27.8 (21.2 - 34.9)	24.0 (18.1 - 30.6)	24.1 (17.3 - 31.3)	25.1 (19.0 - 31.7)
Slope	1.172 (0.954 - 1.431)	1.120 (0.942 - 1.327)	1.128 (0.944 - 1.350)	1.139 (0.947 - 1.370)	1.117 (0.896 - 1.365)	1.157 (0.950 - 1.390)
Scaled Brier (%)	0.82	0.84	0.85	0.67	0.67	0.72
**Females**						
C	0.707 (0.670 - 0.745)	0.711 (0.671 - 0.746)	0.713 (0.673 - 0.749)	0.700 (0.657 - 0.738)	0.696 (0.655 - 0.731)	0.680 (0.638 - 0.720)
Dxy	0.414 (0.340 - 0.490)	0.421 (0.342 - 0.492)	0.427 (0.345 - 0.498)	0.399 (0.313 - 0.476)	0.393 (0.309 - 0.461)	0.360 (0.276 - 0.439)
D	1.244 (0.994 - 1.492)	1.227 (0.985 - 1.459)	1.250 (1.005 - 1.485)	1.142 (0.882 - 1.398)	1.133 (0.887 - 1.377)	1.004 (0.769 - 1.245)
R2D (%)	27.0 (19.1 - 34.7)	26.432 (18.8 - 33.7)	27.2 (19.4 - 34.5)	23.8 (15.7 - 31.8)	23.5 (15.8 - 31.1)	19.4 (12.4 - 27.0)
Slope	1.171 (0.912 - 1.460)	1.053 (0.827 - 1.278)	1.080 (0.847 - 1.304)	1.076 (0.822 - 1.354)	1.058 (0.802 - 1.312)	0.944 (0.708 - 1.203)
Scaled Brier (%)	0.55	0.52	0.56	0.44	0.41	0.30

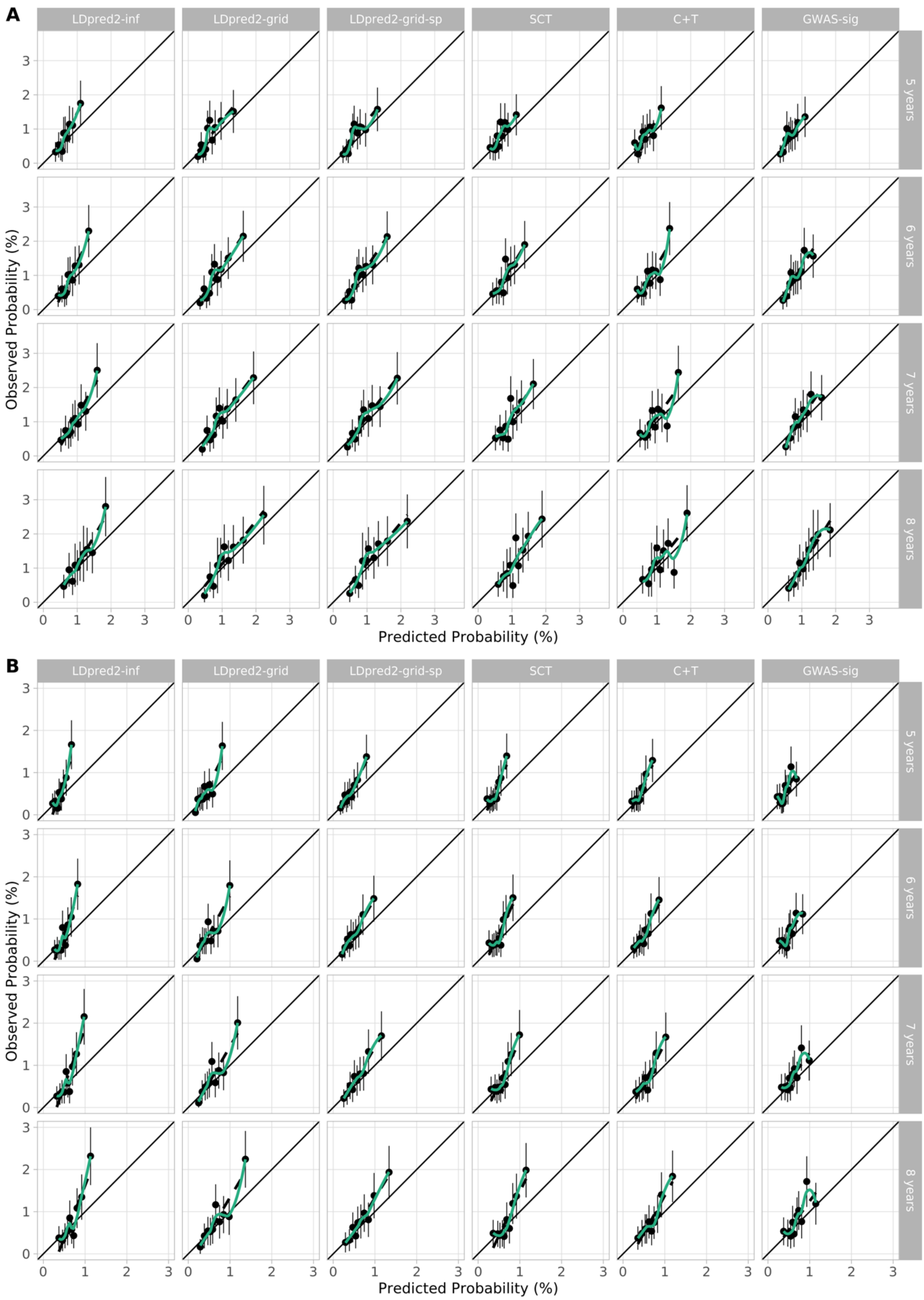

Figure S11. Calibration of PRS in Cox models by sex in the Geographic Validation Cohort. Plots show predicted and observed probability of CRC by tenths of PRS for each model in males (A) and females (B).

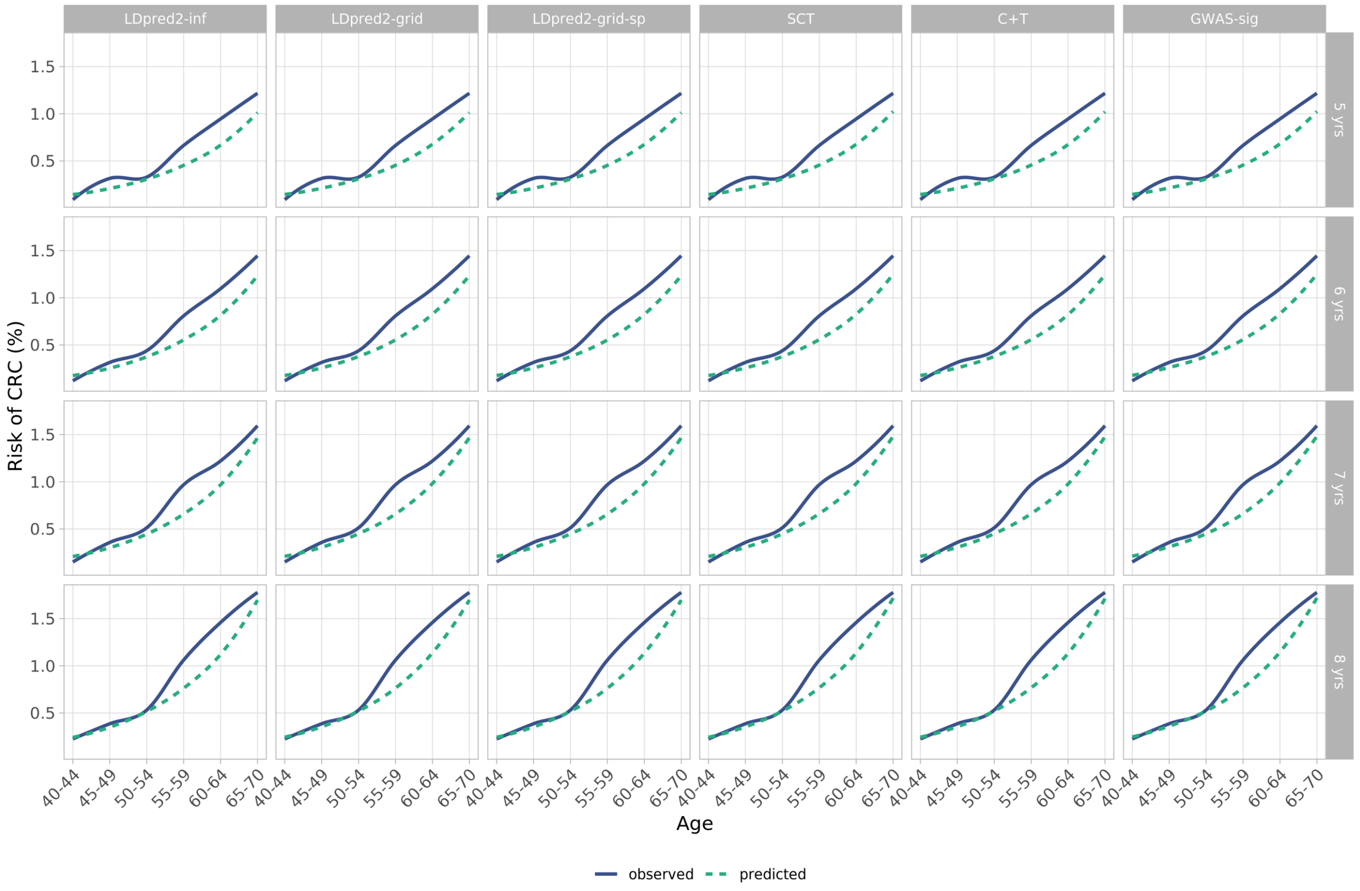

Figure S12. Observed and predicted risk of CRC for PRS Cox models across 5 year age bands in the Geographic Validation Cohort

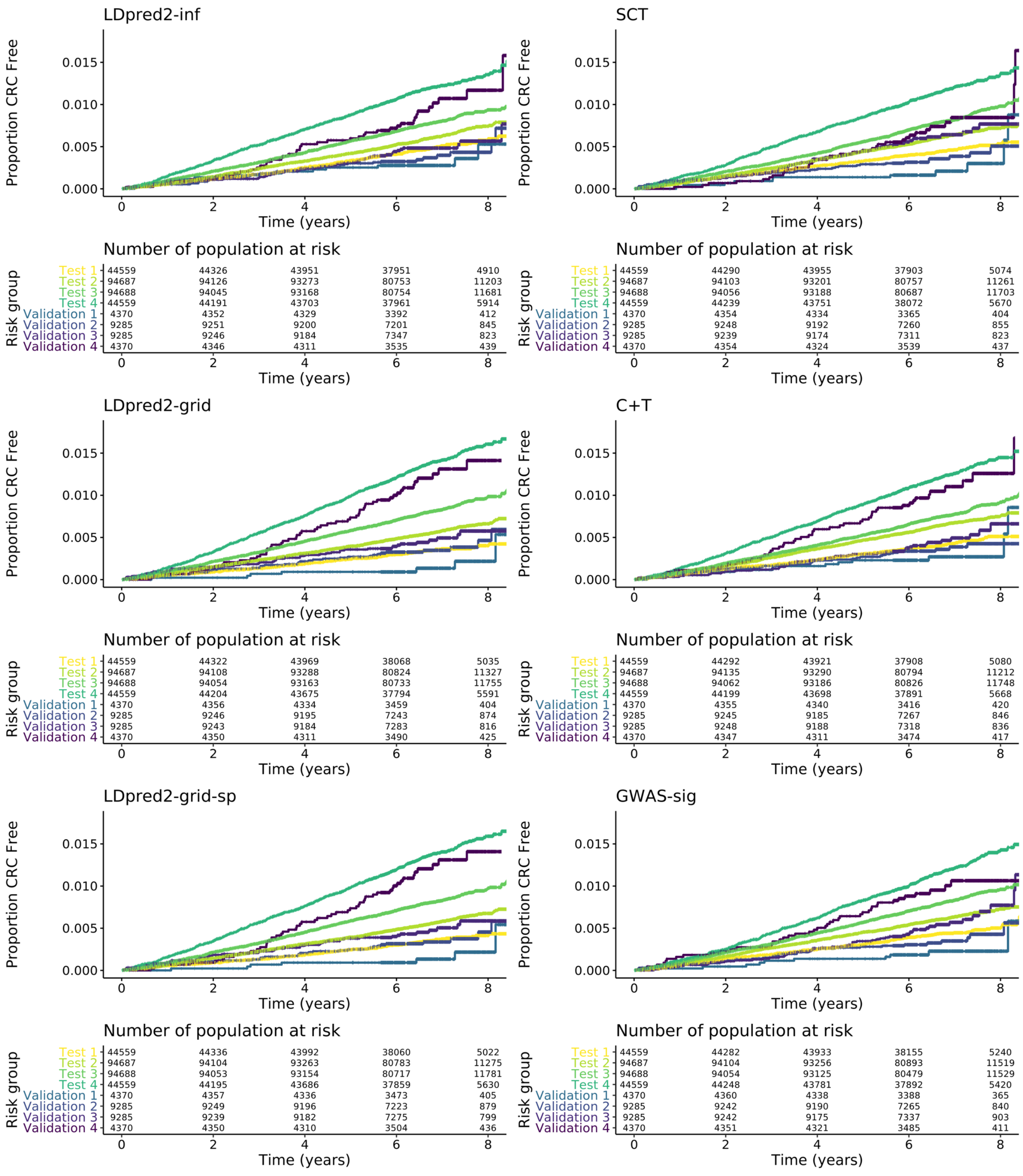

Figure S13. Kaplan-Meier cumulative incidence curves across four risk groups (group 4 being highest risk) for PRS in the Minority Ethnic Validation Cohort compared with the Test Cohort

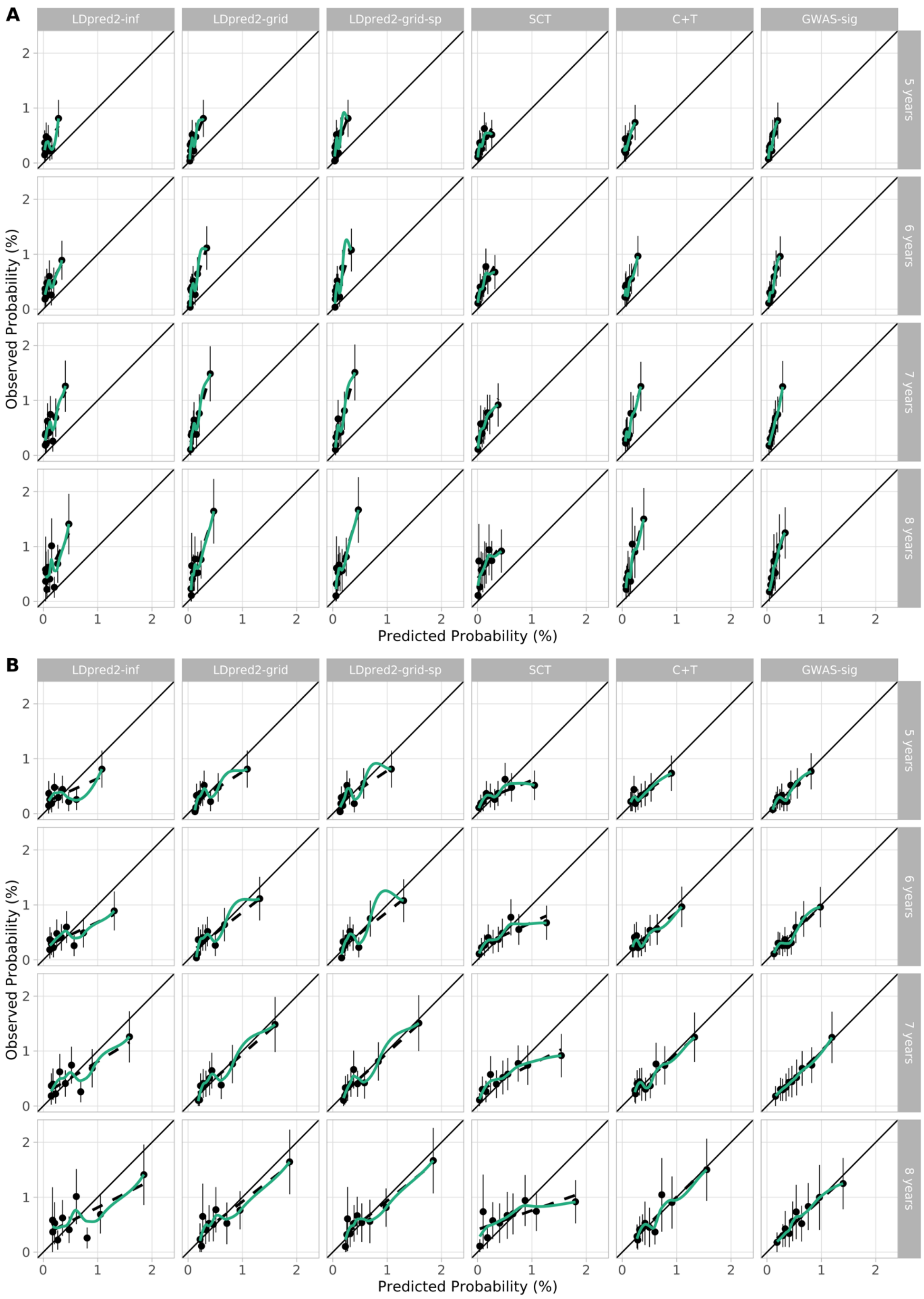

Figure S14. Calibration plots of PRS models in Cox model in the Minority Ethnic Validation Cohort. Plots show predicted and observed probability of CRC by tenths of PRS before (A) and after (B) recalibration for each model.

Table S11. Characteristics of the UKB Integrated Modelling Cohort used for QCancer-10 validation, compared with the QCancer-10 derivation cohort. Values are numbers (%) unless otherwise indicated. CRC – colorectal cancer, NA – not applicable.

	Male UKB cohort (n = 196091)	Male QCancer-10 Derivation (n =2 447 866)	Female UKB cohort (n = 238946)	Female QCancer-10 Derivation (n =2 495 899)
Age (years), mean (SD)	56.7 (8.2)	44.3 (14.8)	56.3 (8.0)	44.9 (15.9)
Ethnicity				
White/not recorded	185813 (94.8)	2 231 641 (91.2)	224316 (94.6)	2 271 520 (91.0)
Indian	2510 (1.3)	42 771 (1.7)	2601 (1.1)	37 773 (1.5)
Pakistani	903 (0.5)	17 169 (0.7)	616 (0.3)	16 893 (0.7)
Bangladeshi	132 (0.1)	17 169 (0.7)	61 (0.0)	13 170 (0.5)
Other Asian	841 (0.4)	24 494 (1.0)	748 (0.3)	27 750 (1.1)
Caribbean	1397 (0.7)	37 003 (1.5)	1412 (0.6)	40 742 (1.6)
Black African	1363 (0.7)	18 553 (0.8)	2498 (1.0)	23 920 (1.0)
Chinese	516 (0.3)	12 493 (0.5)	865 (0.4)	17 702 (0.7)
Other	2616 (1.3)	41 738 (1.7)	3980 (1.7)	46 429 (1.9)
Townsend deprivation index, mean (SD)	-1.3 (3.1)	0.3 (3.6)	-1.4 (3.0)	0.2 (3.6)
BMI (kg/m2), mean (SD)	27.8 (4.2)	26.3 (4.2)	27.0 (5.2)	25.7 (5.0)
Smoking status				
Non-smoker	97088 (49.5)	1 081 822 (44.2)	142569 (59.8)	1 433 446 (57.4)
Ex-smoker	75100 (38.3)	448 480 (18.3)	74934 (31.4)	392 870 (15.7)
Light smoker	9361 (4.8)	351 559 (14.4)	8885 (3.7)	284 482 (11.4)
Moderate smoker	5816 (3.0)	167 089 (6.8)	7235 (3.0)	152 115 (6.1)
Heavy smoker	8726 (4.4)	139 985 (5.7)	4873 (2.0)	86 114 (3.5)
Alcohol intake				
Non-drinker	11985 (6.1)	433 515 (17.7)	22415 (9.4)	753 150 (30.2)
Trivial drinker	41810 (21.3)	585 589 (23.9)	96085 (40.3)	849 734 (34.0)
Light drinker	57817 (29.5)	358 713 (14.7)	76942 (32.3)	295 009 (11.8)
Moderate drinker	60694 (31.0)	486 003 (19.9)	37830 (15.9)	176 644 (7.1)
Heavy drinker	14960 (7.6)	41 223 (1.7)	3797 (1.6)	5332 (0.2)
Very heavy drinker	8825 (4.5)	18 473 (0.8)	1427 (0.6)	3743 (0.1)
Medical history				
Ulcerative colitis	1053 (0.5)	8956 (0.4)	1211 (0.5)	8983 (0.4)
Colorectal polyps	616 (0.3)	3146 (0.1)	612 (0.3)	2447 (0.1)
Diabetes	12893 (6.6)	68 727 (2.8)	7885 (3.3)	53 070 (2.1)
Breast cancer	NA	NA	9448 (4.0)	25 108 (1.0)
Uterine cancer	NA	NA	1030 (0.4)	1987 (0.1)
Ovarian cancer	NA	NA	724 (0.3)	2242 (0.1)
Cervical cancer	NA	NA	1711 (0.7)	3582 (0.1)
Lung cancer	125 (0.1)	1488 (0.1)	NA	NA
Blood cancers	1146 (0.6)	5953 (0.2)	NA	NA
Oral cancer	483 (0.2)	964 (0.0)	NA	NA
Family history of CRC	19505 (9.9)	29 877 (1.2)	22252 (9.3)	43 741 (1.8)

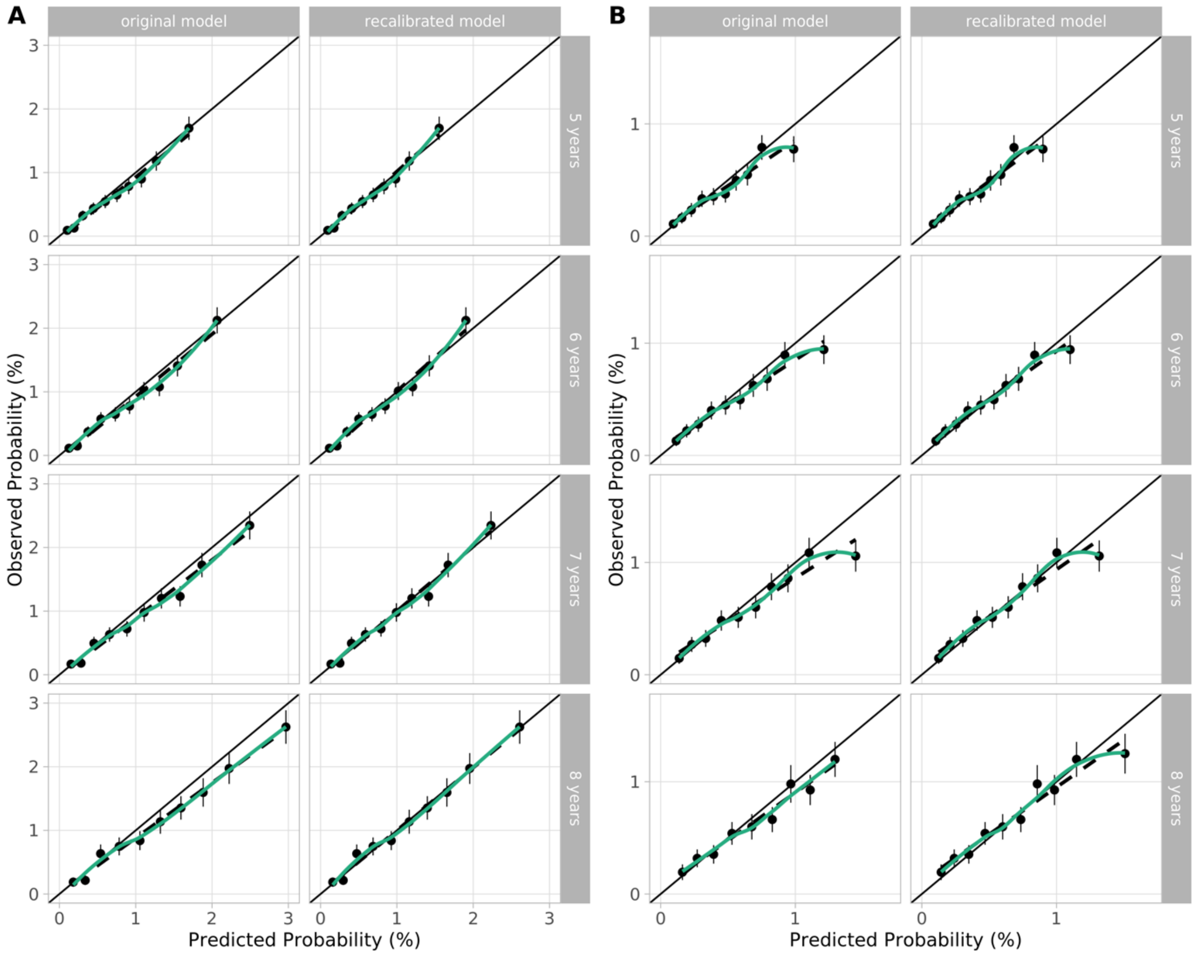

Figure S15. Calibration of QCancer-10 over 5-8 years of follow-up. Plots show predicted and observed probability of CRC by tenths of predicted risk in males (A) and females (B) before and after recalibration.

Table S12. Expected/observed ratio of risk over 5-8 years of follow-up for male and female for QCancer-10+LDP, QCancer-10+GWS, and QCancer-10 models in subgroup analyses.

	Years of follow-up	QCancer-10+LDP	QCancer-10+GWS	QCancer-10
**Family history of CRC**				
Male	5	1.07	1.05	1.02
	6	1.04	1.02	0.99
	7	1.08	1.06	1.04
	8	0.97	0.95	0.93
Female	5	1.28	1.24	1.31
	6	1.22	1.19	1.25
	7	1.26	1.23	1.30
	8	1.19	1.16	1.23
**Minority ethnicity**				
Male	5	0.39	0.40	0.73
	6	0.44	0.45	0.82
	7	0.46	0.47	0.86
	8	0.54	0.55	1.01
Female	5	0.69	0.67	0.74
	6	0.61	0.59	0.65
	7	0.58	0.56	0.62
	8	0.52	0.50	0.56
**White/Not Recorded ethnicitiy**				
Male	5	1.02	1.02	1.01
	6	1.02	1.02	1.01
	7	1.02	1.02	1.01
	8	1.02	1.03	1.02
Female	5	1.01	1.01	1.01
	6	1.02	1.02	1.02
	7	1.02	1.02	1.02
	8	1.02	1.02	1.02

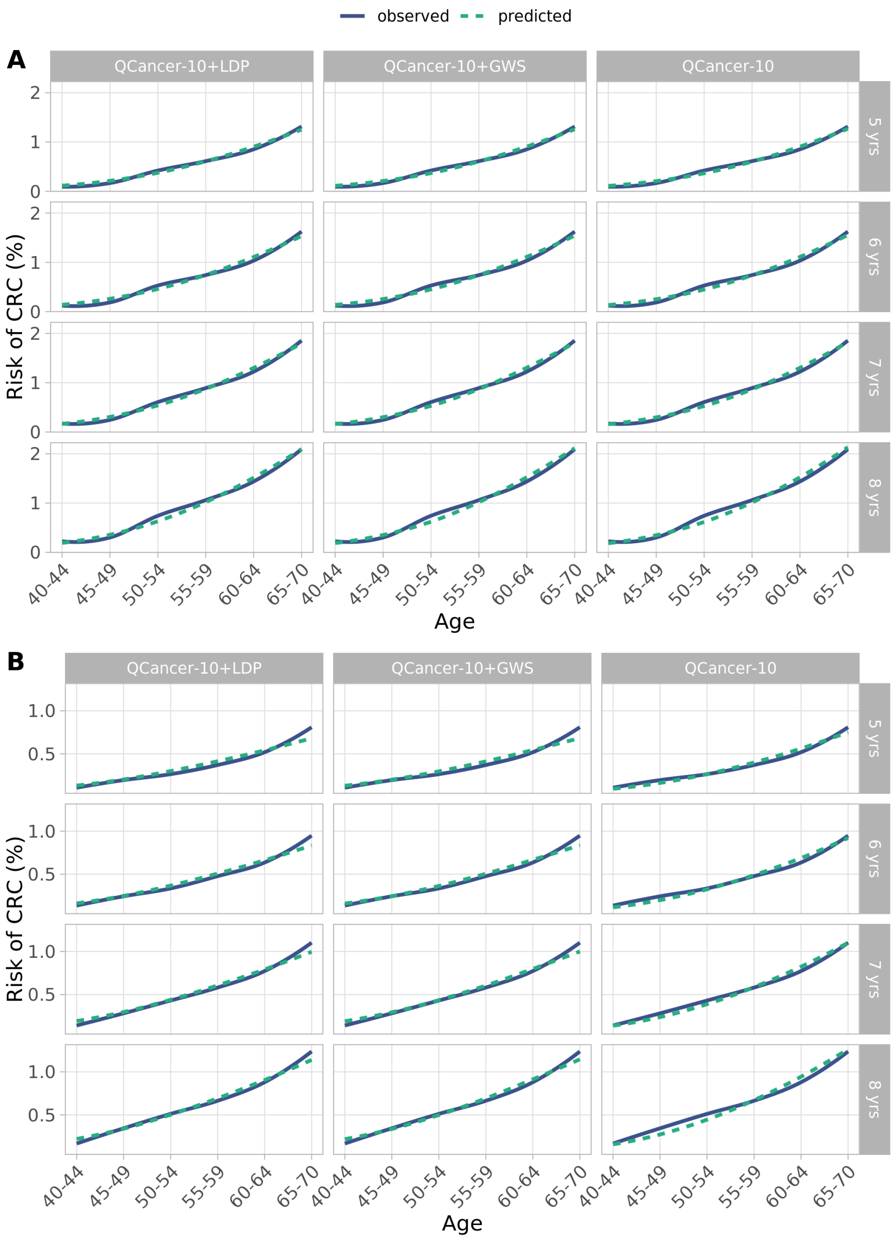

Figure S16. Calibration by age for QCancer-10+LDP, QCancer-10+GWS, and QCancer-10 models. Plots show predicted and observed risk of CRC in 5-year age bands for males (A) and females (B) .

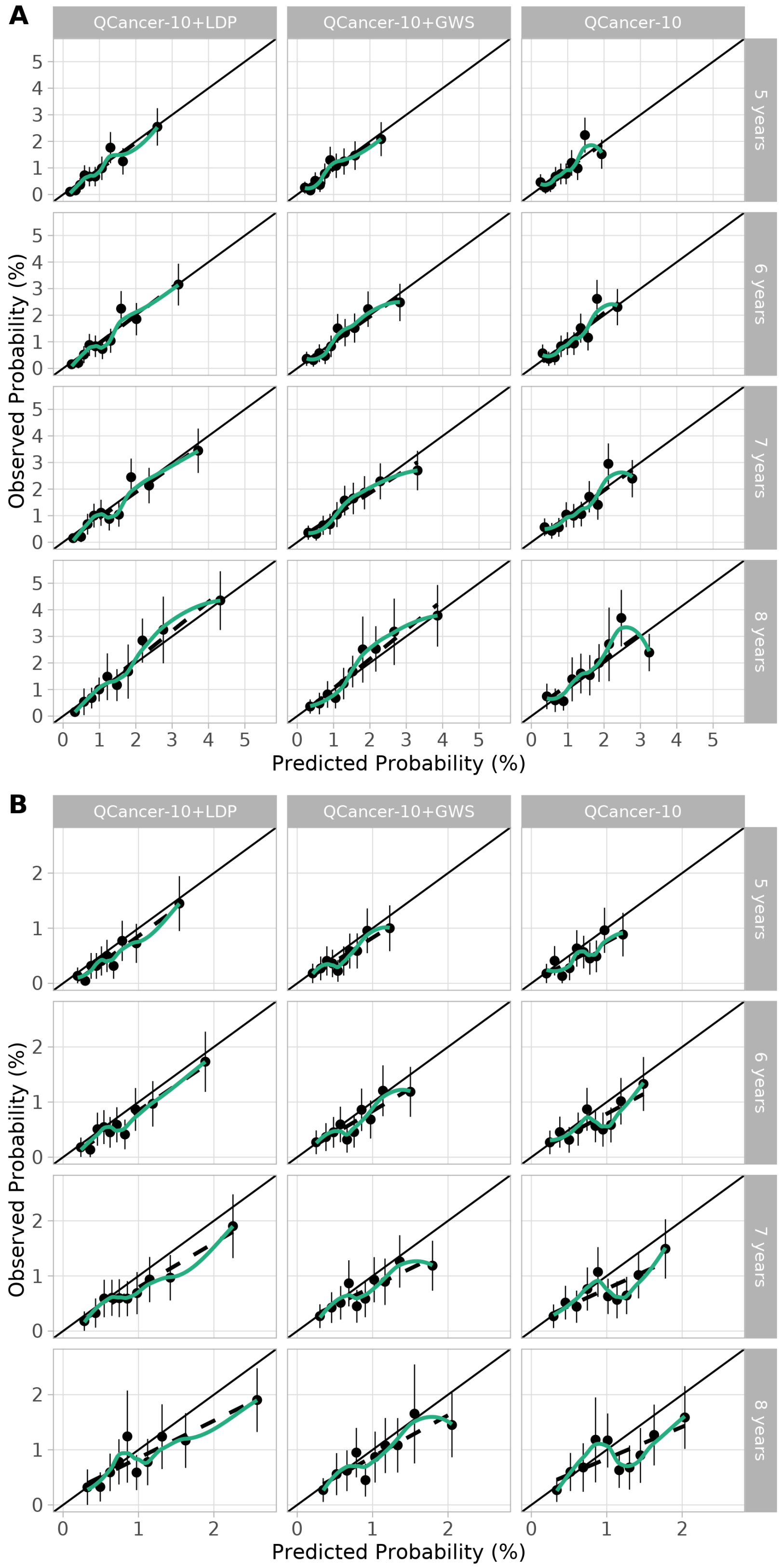

Figure S17. Calibration plots for individuals with a first-degree family history of CRC in QCancer-10+PRS and QCancer-10 models. Plots show predicted and observed probability of CRC by tenths of predicted risk in males (A) and females (B)

QCancer-10+PRS model specification

We confirmed QCancer-10 risk score and PRS fulfilled proportional hazards assumptions. Evaluation of multiple fractional polynomials (MFP) for modelling of these predictors resulted in use of MFP terms for the PRS in the female QCancer-10+LDP model (see Model specification below). Evaluation of interaction terms showed no significant interactions (Table S13). Plots of marginal effects (Figure S18) indicated a reduction in effect of QCancer-10 with increasing PRS score. Given the weakness of the interaction terms relative to the other predictors based on Wald χ^2, we elected not to include interaction terms in the models.

Table S13. Interaction terms in QCancer-10+LDP and QCancer-10+GWS models. Evaluated using MFP terms for female QCancer-10+LDP model

	QCancer-10+LDP	QCancer-10+GWS
**Male**		
QCancer-10 LP	627.71 (<0.001)	630.14 (<0.001)
PRS	387.30 (<0.001)	238.64 (<0.001)
Interaction term	3.75 (0.053)	3.13 (0.077)
**Female**		
QCancer-10 LP	293.43 (<0.001)	296.62 (<0.001)
PRS	297.96 (<0.001)	134.03 (<0.001)
Interaction term	0.15 (0.699)	0.39 (0.532)

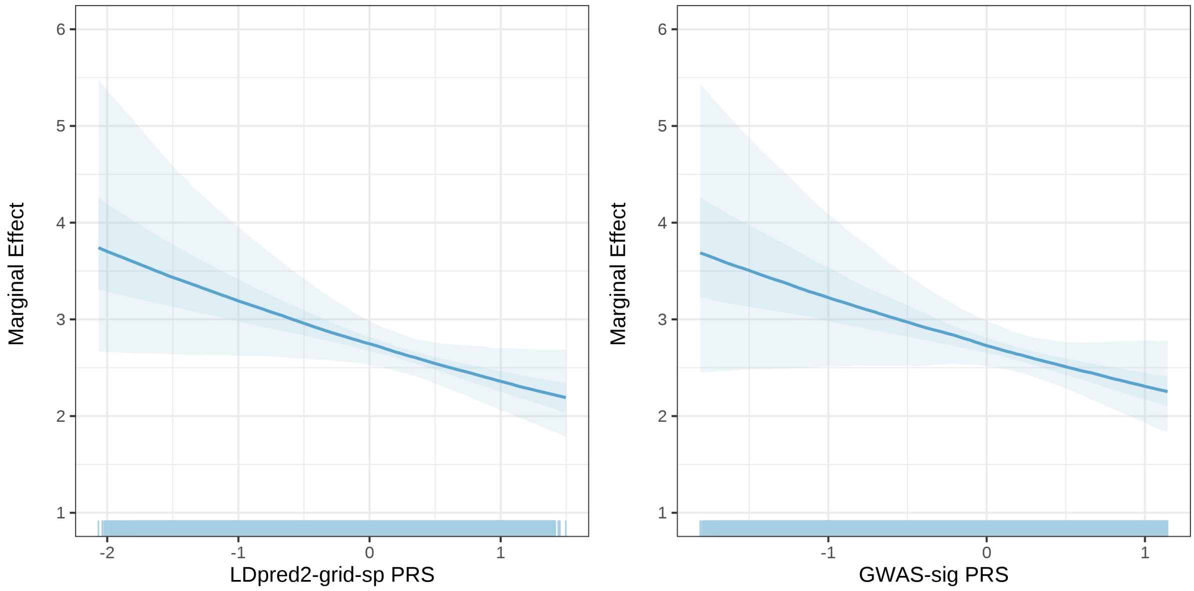

Figure S18. Marginal effect of QCancer-10 risk score in interaction with PRS in male QCancer-10+LDP and QCancer-10+GWS models

QCancer-10+LDP models for males after adjustment for optimism:

$LDpred2\text{-}grid\text{-}sp_{adjusted}$ = 1.04985588662319*LDpred2-grid-sp +
0.090358369892617(if array = UKBL, else 0) +
-0.000646435400853768*PC1 + 0.00203749800858684*PC2 +
-0.00105480195894281*PC3 + -0.00820383472491587*PC4

LP = 0.937200948274397*$LDpred2\text{-}grid\text{-}sp_{adjusted}$ + 0.970057781392185*QCancer-10

Baseline survival function
5 years: 0.995503558989982
6 years: 0.994483895907676
7 years: 0.993524592920923
8 years: 0.992442135730283

QCancer-10+LDP models for females after adjustment for optimism:

$LDpred2\text{-}grid\text{-}sp_{adjusted}$ = 0.981642474963677*LDpred2-grid-sp +
0.0401966124754985 (if array = UKBL, else 0)
+ 0.000393807555057784*PC1 + -0.000202809485666436*PC2 +
-0.000673600666882702*PC3 + -0.00459463357749241*PC4

LP = 0.29625189419672*$(LDpred2\text{-}grid\text{-}sp_{adjusted}+1.4)^2$ + 0.787065583817806*(QCancer-10+0.8)

Baseline survival function
5 years: 0.996690690784853
6 years: 0.99594600188806
7 years: 0.995151013027993
8 years: 0.994454099421112

QCancer-10+GWS models for males after adjustment for optimism:

$GWAS\text{-}sig_{adjusted}$ = 0.8228301262907500*GWAS-sig + 0.0945938015563998(if array = UKBL, else 0) +
-0.00145651937897129*PC1 + 0.0023406651386284*PC2 +
-0.00424001053478569*PC3 + 0.00211400328539279*PC4

LP = 0.915945181249677*$GWAS\text{-}sig_{adjusted}$ + 0.97369868829355*QCancer-10

Baseline survival function
5 years: 0.995329440220753
6 years: 0.994271238507206
7 years: 0.993274810683161
8 years: 0.992138098005285

QCancer-10+GWS models for females after adjustment for optimism:

$GWAS\text{-}sig_{adjusted}$ = 0.68704753784713*GWAS-sig + 0.0404184110549098(if array = UKBL, else 0) +
-0.000461491840519626*PC1 + 0.000199949367935899*PC2 +
-0.00379057680119187*PC3 + 0.00549626461386574*PC4

LP = 0.932748375287305*$GWAS\text{-}sig_{adjusted}$ + 0.792912548535674*QCancer-10

Baseline survival function
5 years: 0.996571629967618
6 years: 0.995800544829255
7 years: 0.994975798123391
8 years: 0.994248190397503

Table S14. Sensitivity analysis of QCancer-10+LDP, QCancer-10+GWS, and QCancer-10 model performance with CRC cases diagnosed within 2 years of recruitment excluded from the analysis

	QCancer-10+LDP	QCancer-10+GWS	QCancer-10
**Males**			
C	0.730 (0.721 - 0.740)	0.720 (0.707 - 0.730)	0.693 (0.682 - 0.704)
Dxy	0.865 (0.860 - 0.870)	0.860 (0.853 - 0.865)	0.847 (0.841 - 0.852)
D	1.283 (1.224 - 1.341)	1.219 (1.150 - 1.284)	1.058 (0.987 - 1.121)
R2D (%)	28.2 (26.3 - 30.0)	26.2 (24.0 - 28.2)	21.1 (18.9 - 23.0)
**Females**			
C	0.687 (0.673 - 0.699)	0.664 (0.647 - 0.679)	0.645 (0.631 - 0.659)
Dxy	0.843 (0.837 - 0.850)	0.832 (0.824 - 0.839)	0.822 (0.816 - 0.830)
D	1.060 (0.983 - 1.141)	0.905 (0.810 - 0.986)	0.769 (0.695 - 0.847)
R2D (%)	21.2 (18.7 - 23.7)	16.3 (13.6 - 18.8)	12.4 (10.3 - 14.6)

Table S15. Performance of QCancer-10+LDP, QCancer-10+GWS, and QCancer-10 models across age groups (<50 years, 50-59 years, ≥60 years)

		QCancer-10+LDP	QCancer-10+GWS	QCancer-10
**Males**				
<50 years	C	0.666 (0.608 - 0.720)	0.685 (0.637 - 0.738)	0.621 (0.559 - 0.676)
	Dxy	0.833 (0.804 - 0.860)	0.843 (0.819 - 0.869)	0.810 (0.779 - 0.838)
	D	1.071 (0.733 - 1.400)	1.074 (0.768 - 1.405)	0.678 (0.370 - 0.979)
	R2D (%)	21.5 (11.4 - 31.9)	21.6 (12.4 - 32.0)	9.9 (3.2 - 18.6)
	Slope	0.956 (0.651 - 1.265)	1.062 (0.743 - 1.421)	0.865 (0.461 - 1.269)
50-59 years	C	0.671 (0.648 - 0.694)	0.643 (0.620 - 0.667)	0.590 (0.565 - 0.615)
	Dxy	0.836 (0.824 - 0.847)	0.822 (0.810 - 0.833)	0.795 (0.782 - 0.807)
	D	1.009 (0.874 - 1.147)	0.808 (0.673 - 0.942)	0.516 (0.382 - 0.655)
	R2D (%)	19.6 (15.4 - 23.9)	13.5 (9.8 - 17.5)	6.0 (3.4 - 9.3)
	Slope	1.044 (0.896 - 1.190)	0.938 (0.769 - 1.101)	0.849 (0.623 - 1.089)
⩾60 years	C	0.656 (0.641 - 0.671)	0.636 (0.621 - 0.652)	0.608 (0.592 - 0.623)
	Dxy	0.828 (0.820 - 0.836)	0.818 (0.810 - 0.826)	0.804 (0.796 - 0.812)
	D	0.860 (0.776 - 0.945)	0.769 (0.686 - 0.861)	0.578 (0.490 - 0.664)
	R2D (%)	15.0 (12.6 - 17.6)	12.4 (10.1 - 15.0)	7.4 (5.4 - 9.5)
	Slope	0.982 (0.886 - 1.081)	1.028 (0.914 - 1.156)	1.163 (0.986 - 1.327)
**Females**				
<50 years	C	0.675 (0.631 - 0.724)	0.641 (0.595 - 0.687)	0.594 (0.540 - 0.644)
	Dxy	0.837 (0.815 - 0.862)	0.821 (0.798 - 0.844)	0.797 (0.770 - 0.822)
	D	1.059 (0.784 - 1.349)	0.756 (0.505 - 1.009)	0.483 (0.189 - 0.773)
	R2D (%)	21.1 (12.8 - 30.3)	12.0 (5.7 - 19.6)	5.3 (0.8 - 12.5)
	Slope	1.186 (0.891 - 1.479)	1.020 (0.680 - 1.374)	0.729 (0.289 - 1.158)
50-59 years	C	0.625 (0.595 - 0.652)	0.629 (0.601 - 0.653)	0.573 (0.547 - 0.599)
	Dxy	0.813 (0.797 - 0.826)	0.814 (0.800 - 0.826)	0.787 (0.773 - 0.800)
	D	0.785 (0.602 - 0.951)	0.709 (0.551 - 0.858)	0.438 (0.280 - 0.597)
	R2D (%)	12.8 (8.0 - 17.8)	10.7 (6.8 - 14.9)	4.4 (1.8 - 7.8)
	Slope	0.975 (0.763 - 1.161)	1.038 (0.797 - 1.277)	0.800 (0.500 - 1.095)
⩾60 years	C	0.627 (0.607 - 0.645)	0.596 (0.576 - 0.617)	0.549 (0.530 - 0.569)
	Dxy	0.813 (0.804 - 0.822)	0.798 (0.788 - 0.808)	0.775 (0.765 - 0.785)
	D	0.701 (0.598 - 0.808)	0.517 (0.408 - 0.629)	0.257 (0.158 - 0.365)
	R2D (%)	10.5 (7.9 - 13.5)	6.0 (3.8 - 8.6)	1.5 (0.6 - 3.1)
	Slope	0.903 (0.784 - 1.027)	0.849 (0.668 - 1.038)	0.563 (0.328 - 0.804)

Table S16. Sensitivity, specificity, detection rate, and false positive rate of QCancer-10+GWS models for CRC diagnosis across top 25 centiles of absolute risk in males and females

Centiles	Population per centile	Absolute 5-year risk centile cut-off (%)	Cases per centile	Cumulative % cases based on absolute risk (sensitivity)	Specificity (%)	Detection Rate (%)
**Men**						
1	1960	2.40	70	3.7	99.0	0.04
2	1961	2.11	60	6.9	98.1	0.07
3	1961	1.93	39	9.0	97.1	0.09
4	1961	1.80	70	12.7	96.1	0.12
5	1961	1.70	48	15.2	95.1	0.15
6	1961	1.62	37	17.2	94.1	0.17
7	1961	1.55	51	19.9	93.1	0.19
8	1961	1.49	45	22.3	92.1	0.21
9	1961	1.44	41	24.5	91.1	0.24
10	1961	1.39	38	26.5	90.2	0.25
11	1961	1.34	51	29.2	89.2	0.28
12	1960	1.30	27	30.6	88.2	0.29
13	1961	1.27	31	32.2	87.2	0.31
14	1961	1.23	44	34.5	86.2	0.33
15	1961	1.20	38	36.5	85.2	0.35
16	1961	1.17	21	37.6	84.2	0.36
17	1961	1.14	28	39.1	83.2	0.38
18	1961	1.11	35	40.9	82.2	0.39
19	1961	1.09	41	43.1	81.2	0.42
20	1961	1.06	24	44.4	80.2	0.43
21	1961	1.04	25	45.7	79.2	0.44
22	1961	1.01	30	47.3	78.2	0.46
23	1960	0.99	39	49.4	77.3	0.48
24	1961	0.97	25	50.7	76.3	0.49
25	1961	0.95	33	52.4	75.3	0.51
**Women**						
1	2384	1.18	38	2.6	99.0	0.02
2	2385	1.05	37	5.1	98.0	0.03
3	2385	0.98	34	7.4	97.0	0.05
4	2385	0.92	29	9.4	96.0	0.06
5	2385	0.88	29	11.4	95.0	0.07
6	2385	0.85	36	13.9	94.0	0.09
7	2385	0.82	28	15.8	93.1	0.10
8	2385	0.79	27	17.7	92.1	0.11
9	2385	0.77	30	19.8	91.1	0.12
10	2385	0.75	24	21.4	90.1	0.13
11	2385	0.73	32	23.6	89.1	0.14
12	2385	0.71	34	25.9	88.1	0.16
13	2385	0.70	28	27.8	87.1	0.17
14	2385	0.68	16	28.9	86.1	0.18
15	2385	0.67	24	30.5	85.1	0.19
16	2385	0.65	29	32.5	84.1	0.20
17	2385	0.64	21	33.9	83.1	0.21
18	2385	0.63	20	35.3	82.1	0.22
19	2385	0.62	31	37.4	81.1	0.23
20	2385	0.61	26	39.2	80.1	0.24
21	2385	0.60	26	41.0	79.1	0.25
22	2385	0.59	24	42.6	78.1	0.26
23	2385	0.58	25	44.3	77.1	0.27
24	2385	0.57	13	45.2	76.1	0.28
25	2385	0.56	31	47.3	75.1	0.29

Table S17. Sensitivity, specificity, detection rate, and false positive rate of QCancer-10 for CRC diagnosis across top 25 centiles of absolute risk in men and women. Calculated following recalibration of the QCancer-10 model.

Centiles	Population per centile	Absolute 5-year risk centile cut-off (%)	Cases per centile	Cumulative % cases based on absolute risk (sensitivity)	Specificity (%)	Detection Rate (%)
**Men**						
1	1960	1.90	49	2.6	99.0	0.02
2	1961	1.71	44	4.9	98.0	0.05
3	1961	1.60	51	7.6	97.0	0.07
4	1961	1.53	48	10.1	96.1	0.10
5	1961	1.47	51	12.8	95.1	0.12
6	1961	1.42	43	15.1	94.1	0.15
7	1961	1.38	35	16.9	93.1	0.16
8	1961	1.34	52	19.7	92.1	0.19
9	1961	1.31	36	21.6	91.1	0.21
10	1961	1.28	41	23.7	90.1	0.23
11	1961	1.25	37	25.7	89.1	0.25
12	1960	1.22	42	27.9	88.2	0.27
13	1961	1.20	38	29.9	87.2	0.29
14	1961	1.18	28	31.4	86.2	0.30
15	1961	1.16	39	33.5	85.2	0.32
16	1961	1.14	28	34.9	84.2	0.34
17	1961	1.12	36	36.8	83.2	0.36
18	1961	1.10	33	38.6	82.2	0.37
19	1961	1.08	27	40.0	81.2	0.39
20	1961	1.07	23	41.2	80.2	0.40
21	1961	1.05	27	42.6	79.2	0.41
22	1961	1.03	31	44.3	78.2	0.43
23	1960	1.01	25	45.6	77.2	0.44
24	1961	1.00	36	47.5	76.2	0.46
25	1961	0.98	22	48.7	75.2	0.47
**Women**						
1	2336	1.10	24	1.6	99.0	0.01
2	2344	0.98	38	4.3	98.1	0.03
3	2364	0.91	22	5.8	97.1	0.04
4	2422	0.86	21	7.2	96.1	0.04
5	2375	0.82	34	9.5	95.1	0.06
6	2203	0.80	18	10.8	94.1	0.07
7	2598	0.78	24	12.4	93.1	0.08
8	1827	0.76	25	14.1	92.3	0.09
9	2991	0.75	24	15.8	91.0	0.10
10	900	0.74	8	16.3	90.7	0.10
11	3846	0.72	38	18.9	89.1	0.12
12	2392	0.71	22	20.4	88.1	0.12
13	1543	0.70	20	21.8	87.4	0.13
14	2417	0.69	35	24.2	86.4	0.15
15	3216	0.68	33	26.5	85.1	0.16
16	2294	0.67	26	28.3	84.1	0.17
17	2328	0.66	23	29.8	83.1	0.18
18	2499	0.65	16	30.9	82.1	0.19
19	2306	0.64	23	32.5	81.1	0.20
20	2434	0.63	30	34.6	80.1	0.21
21	2418	0.62	15	35.6	79.1	0.22
22	2058	0.61	27	37.4	78.2	0.23
23	2388	0.60	16	38.5	77.2	0.24
24	2542	0.59	19	39.8	76.2	0.24
25	2539	0.58	27	41.7	75.1	0.25

Table S18. Sensitivity, specificity, detection rate, and false positive rate of QCancer-10+LDP across top 25 centiles of relative risk. Risk is calculated relative to an individual of the same age and sex, of white-British ethnicity, with no CRC risk factors, BMI of 25, mean Townsend Deprivation Score, and mean PRS.

Centiles	Population per centile	Relative risk centile cut-off (%)	Cases per centile	Cumulative % cases based on absolute risk (sensitivity)	Specificity (%)	Detection Rate (%)
**Men**						
1	1960	4.82	51	2.7	99.0	0.03
2	1961	4.15	58	5.8	98.0	0.06
3	1961	3.78	36	7.7	97.0	0.07
4	1961	3.52	44	10.0	96.1	0.10
5	1961	3.33	27	11.4	95.1	0.11
6	1961	3.16	42	13.6	94.1	0.13
7	1961	3.02	39	15.7	93.1	0.15
8	1961	2.90	24	17.0	92.1	0.16
9	1961	2.80	31	18.6	91.1	0.18
10	1961	2.71	44	20.9	90.1	0.20
11	1961	2.63	36	22.8	89.1	0.22
12	1960	2.56	34	24.6	88.1	0.24
13	1961	2.49	28	26.1	87.1	0.25
14	1961	2.43	41	28.3	86.1	0.27
15	1961	2.37	33	30.0	85.1	0.29
16	1961	2.32	23	31.2	84.1	0.30
17	1961	2.27	25	32.5	83.1	0.31
18	1961	2.22	24	33.8	82.2	0.33
19	1961	2.17	29	35.3	81.2	0.34
20	1961	2.13	34	37.1	80.2	0.36
21	1961	2.09	28	38.6	79.2	0.37
22	1961	2.05	30	40.2	78.2	0.39
23	1960	2.02	28	41.7	77.2	0.40
24	1961	1.98	22	42.9	76.2	0.41
25	1961	1.95	28	44.4	75.2	0.43
**Women**						
1	2384	3.88	49	3.4	99.1	0.02
2	2385	3.27	33	5.7	98.0	0.03
3	2385	2.91	39	8.4	97.0	0.05
4	2385	2.67	32	10.6	96.0	0.06
5	2385	2.50	22	12.1	95.0	0.07
6	2385	2.36	31	14.2	94.1	0.09
7	2385	2.24	28	16.1	93.1	0.10
8	2385	2.15	23	17.7	92.1	0.11
9	2385	2.07	18	18.9	91.1	0.12
10	2385	2.00	31	21.0	90.1	0.13
11	2385	1.93	23	22.6	89.1	0.14
12	2385	1.88	35	25.0	88.1	0.15
13	2385	1.82	19	26.3	87.1	0.16
14	2385	1.78	17	27.5	86.1	0.17
15	2385	1.73	17	28.7	85.1	0.17
16	2385	1.70	19	30.0	84.1	0.18
17	2385	1.66	19	31.3	83.1	0.19
18	2385	1.62	17	32.5	82.1	0.20
19	2385	1.59	19	33.8	81.1	0.21
20	2385	1.56	19	35.1	80.1	0.21
21	2385	1.53	28	37.0	79.1	0.23
22	2385	1.50	18	38.2	78.1	0.23
23	2385	1.48	22	39.7	77.1	0.24
24	2385	1.46	20	41.1	76.1	0.25
25	2385	1.43	22	42.6	75.1	0.26

Table S19. Sensitivity, specificity, detection rate, and false positive rate of QCancer-10+GWS across top 25 centiles of relative risk. Risk is calculated relative to an individual of the same age and sex, of white-British ethnicity, with no CRC risk factors, BMI of 25, mean Townsend Deprivation Score, and mean PRS.

Centiles	Population per centile	Relative risk centile cut-off (%)	Cases per centile	Cumulative % cases based on relative risk (sensitivity)	Specificity (%)	Detection Rate (%)
**Men**						
1	1960	4.00	36	1.9	99.0	0.02
2	1961	3.48	44	4.2	98.0	0.04
3	1961	3.21	41	6.4	97.0	0.06
4	1961	3.02	46	8.8	96.0	0.09
5	1961	2.87	28	10.3	95.1	0.10
6	1961	2.75	34	12.1	94.1	0.12
7	1961	2.65	34	13.9	93.1	0.13
8	1961	2.56	31	15.5	92.1	0.15
9	1961	2.48	40	17.6	91.1	0.17
10	1961	2.42	43	19.9	90.1	0.19
11	1961	2.36	28	21.4	89.1	0.21
12	1960	2.30	34	23.2	88.1	0.22
13	1961	2.25	27	24.6	87.1	0.24
14	1961	2.21	22	25.8	86.1	0.25
15	1961	2.16	24	27.1	85.1	0.26
16	1961	2.12	23	28.3	84.1	0.27
17	1961	2.08	30	29.9	83.1	0.29
18	1961	2.05	19	30.9	82.1	0.30
19	1961	2.01	33	32.6	81.1	0.31
20	1961	1.98	29	34.1	80.1	0.33
21	1961	1.95	26	35.5	79.1	0.34
22	1961	1.92	25	36.8	78.1	0.36
23	1960	1.89	28	38.3	77.1	0.37
24	1961	1.86	17	39.2	76.1	0.38
25	1961	1.84	28	40.7	75.2	0.39
**Women**						
1	2384	2.63	21	1.4	99.0	0.01
2	2385	2.36	33	3.7	98.0	0.02
3	2385	2.22	26	5.5	97.0	0.03
4	2385	2.11	19	6.8	96.0	0.04
5	2385	2.02	22	8.3	95.0	0.05
6	2385	1.95	18	9.5	94.0	0.06
7	2385	1.89	22	11.0	93.0	0.07
8	2385	1.84	24	12.6	92.0	0.08
9	2385	1.79	27	14.5	91.0	0.09
10	2385	1.76	21	15.9	90.0	0.10
11	2385	1.72	22	17.4	89.0	0.11
12	2385	1.69	21	18.8	88.0	0.12
13	2385	1.66	18	20.0	87.0	0.12
14	2385	1.63	25	21.7	86.0	0.13
15	2385	1.60	19	23.0	85.1	0.14
16	2385	1.58	22	24.5	84.1	0.15
17	2385	1.56	21	25.9	83.1	0.16
18	2385	1.53	30	28.0	82.1	0.17
19	2385	1.51	22	29.5	81.1	0.18
20	2385	1.49	16	30.6	80.1	0.19
21	2385	1.47	18	31.8	79.1	0.20
22	2385	1.46	20	33.2	78.1	0.20
23	2385	1.44	22	34.7	77.1	0.21
24	2385	1.42	21	36.1	76.1	0.22
25	2385	1.41	20	37.5	75.1	0.23

Table S20. Fold-increase in absolute risk between 95th centile and median risk for QCancer-10+LDP, QCancer-10+GWS, and QCancer-10 models

	QCancer-10+LDP	QCancer-10+GWS	QCancer-10
Males	3.47	3.06	2.37
Females	2.77	2.35	2.06

Table S21. Percentage of population and cases with relative risk > 2.2 for QCancer-10+LDP, QCancer-10+GWS, and QCancer-10 models

	QCancer-10+LDP		QCancer-10+GWS		QCancer-10	
	Males	Females	Males	Females	Males	Females
% population with RR > 2.2	18.4	7.4	14.2	3.2	4.1	1.2
% of individuals with RR > 2.2 without FDRCRC	76.0	69.8	70.5	43.4	29.4	30.3
% cases with RR > 2.2	34.5	16.7	26.0	5.8	4.9	1.6

Table S22. Net benefit and test trade-off across threshold probabilities of 0.5% to 2% for QCancer-10+LDP, QCancer-10+GWS, and QCancer-10 models

	Threshold Probability(%)	QCancer-10+LDP	QCancer-10+GWS	QCancer-10	Δ with addition of LDP PRS	Test trade-off
Male	0.5	0.00698	0.00693	0.00682	0.00016	6181
	1.0	0.00430	0.00405	0.00362	0.00068	1478
	1.5	0.00254	0.00219	0.00168	0.00086	1165
	2.0	0.00137	0.00113	0.00075	0.00062	1611
Female	0.5	0.00273	0.00272	0.00253	0.00020	5087
	1.0	0.00098	0.00084	0.00042	0.00056	1789
	1.5	0.00036	0.00011	-0.00004	0.00040	2499
	2.0	0.00011	0.00000	-0.00006	0.00017	5830

Table S23. Unnecessary interventions avoided per 100 individuals across threshold probabilities of 0.5% to 2% for QCancer-10+LDP, QCancer-10+GWS, and QCancer-10 models

	Threshold Probability(%)	QCancer-10+LDP	QCancer-10+GWS	QCancer-10	Δ with addition of LDP PRS
Male	0.5	15.5	14.6	12.3	3.2
	1.0	30.9	28.4	24.2	6.7
	1.5	42.2	39.9	36.5	5.6
	2.0	50.8	49.7	47.8	3.0
Female	0.5	16.9	16.7	13.0	3.8
	1.0	40.4	39.1	34.9	5.5
	1.5	56.0	54.4	53.4	2.6
	2.0	65.9	65.4	65.1	0.8

References

1. Law PJ, Timofeeva M, Fernandez-Rozadilla C, et al. Association analyses identify 31 new risk loci for colorectal cancer susceptibility. *Nat Commun*. 2019;**10**(1):2154.DOI: 10.1038/s41467-019-09775-w
2. Aragon TJ. epitools: Epidemiology Tools. 2020 https://CRAN.R-project.org/package=epitools.
3. Therneau T. A Package for Survival Analysis in R. 2015 https://CRAN.R-project.org/package=survival.
4. Prive F, Arbel J, Vilhjalmsson BJ. LDpred2: better, faster, stronger. *Bioinformatics*. 2020;**36**(22-23):5424–31.DOI: 10.1093/bioinformatics/btaa1029
5. Collins GS, Ogundimu EO, Altman DG. Sample size considerations for the external validation of a multivariable prognostic model: a resampling study. *Stat Med*. 2016;**35**(2):214–26.DOI: 10.1002/sim.6787
6. Huyghe JR, Bien SA, Harrison TA, et al. Discovery of common and rare genetic risk variants for colorectal cancer. *Nat Genet*. 2019;**51**(1):76–87.DOI: 10.1038/s41588-018-0286-6
7. Bigdeli TB, Lee D, Webb BT, et al. A simple yet accurate correction for winner's curse can predict signals discovered in much larger genome scans. *Bioinformatics*. 2016;**32**(17):2598–603.DOI: 10.1093/bioinformatics/btw303
8. Prive F, Vilhjalmsson BJ, Aschard H, Blum MGB. Making the Most of Clumping and Thresholding for Polygenic Scores. *Am J Hum Genet*. 2019;**105**(6):1213–21.DOI: 10.1016/j.ajhg.2019.11.001
9. Lloyd-Jones LR, Zeng J, Sidorenko J, et al. Improved polygenic prediction by Bayesian multiple regression on summary statistics. *Nat Commun*. 2019;**10**.DOI: ARTN 5086
10.1038/s41467-019-12653-0
10. Choi SW, Mak TS, O'Reilly PF. Tutorial: a guide to performing polygenic risk score analyses. *Nat Protoc*. 2020;**15**(9):2759-72.DOI: 10.1038/s41596-020-0353-1
11. ClinRisk Ltd. QCancer®(15yr,colorectal). 2015 https://qcancer.org/15yr/colorectal/.
12. Usher-Smith JA, Harshfield A, Saunders CL, et al. External validation of risk prediction models for incident colorectal cancer using UK Biobank. *Br J Cancer*. 2018;**118**(5):750–9.DOI: 10.1038/bjc.2017.463
13. Riley RD, Snell KI, Ensor J, et al. Minimum sample size for developing a multivariable prediction model: PART II - binary and time-to-event outcomes. *Stat Med*. 2019;**38**(7):1276–96.DOI: 10.1002/sim.7992
14. Riley RD, Ensor J, Snell KIE, et al. Calculating the sample size required for developing a clinical prediction model. *BMJ*. 2020;**368**.DOI: ARTN m441
10.1136/bmj.m441
15. Royston P, Sauerbrei W. Improving the robustness of fractional polynomial models by preliminary covariate transformation: A pragmatic approach. *Comput Stat Data An*. 2007;**51**(9):4240–53.DOI: 10.1016/j.csda.2006.05.006
16. Vickers AJ, Elkin EB. Decision curve analysis: a novel method for evaluating prediction models. *Med Decis Making*. 2006;**26**(6):565-74.DOI: 10.1177/0272989X06295361
17. Collins GS, Reitsma JB, Altman DG, Moons KG. Transparent Reporting of a multivariable prediction model for Individual Prognosis Or Diagnosis (TRIPOD). *Ann Intern Med*. 2015;**162**(10):735–6.DOI: 10.7326/L15-5093-2
18. Van Calster B, Wynants L, Verbeek JFM, et al. Reporting and Interpreting Decision Curve Analysis: A Guide for Investigators. *European urology*. 2018;**74**(6):796-804.DOI: 10.1016/j.eururo.2018.08.038
19. Vickers AJ, Cronin AM, Elkin EB, Gonen M. Extensions to decision curve analysis, a novel method for evaluating diagnostic tests, prediction models and molecular markers. *BMC Med Inform Decis Mak*. 2008;**8**:53.DOI: 10.1186/1472-6947-8-53
20. NHS England, NHS Improvement. Delivering a 'Net Zero' National Health Service. 2020.
21. Vickers AJ, van Calster B, Steyerberg EW. A simple, step-by-step guide to interpreting decision curve analysis. *Diagn Progn Res*. 2019;**3**:18.DOI: 10.1186/s41512-019-0064-7
22. Quintero E, Castells A, Bujanda L, et al. Colonoscopy versus fecal immunochemical testing in colorectal-cancer screening. *N Engl J Med*. 2012;**366**(8):697-706.DOI: 10.1056/NEJMoa1108895
23. Kim DH, Pickhardt PJ, Taylor AJ, et al. CT colonography versus colonoscopy for the detection of advanced neoplasia. *N Engl J Med*. 2007;**357**(14):1403-12.DOI: 10.1056/NEJMoa070543
24. Stoop EM, de Haan MC, de Wijkerslooth TR, et al. Participation and yield of colonoscopy versus non-cathartic CT colonography in population-based screening for colorectal cancer: a randomised controlled trial. *Lancet Oncol*. 2012;**13**(1):55-64.DOI: 10.1016/S1470-2045(11)70283-2
25. Bretthauer M, Kaminski MF, Loberg M, et al. Population-Based Colonoscopy Screening for Colorectal Cancer: A Randomized Clinical Trial. *JAMA Intern Med*. 2016;**176**(7):894–902.DOI: 10.1001/jamainternmed.2016.0960
26. Vickers AJ, Van Calster B, Steyerberg EW. Net benefit approaches to the evaluation of prediction models, molecular markers, and diagnostic tests. *BMJ*. 2016;**352**:i6.DOI: 10.1136/bmj.i6

27. Prive F, Aschard H, Ziyatdinov A, Blum MGB. Efficient analysis of large-scale genome-wide data with two R packages: bigstatsr and bigsnpr. *Bioinformatics*. 2018;**34**(16):2781–7.DOI: 10.1093/bioinformatics/bty185
28. Harrell FE. rms: Regression Modeling Strategies. 2019 https://CRAN.R-project.org/package=rms.
29. Ambler G, Benner A. mfp: Multivariable Fractional Polynomials. 2015 https://CRAN.R-project.org/package=mfp.
30. Wickham H, Averick M, Bryan J, et al. Welcome to the tidyverse. *Journal of Open Source Software*. 2019;**4**(43):1686.DOI: https://doi.org/10.21105/joss.01686
31. Office for National Statistics. Cancer registration statistics, England [Available from: https://www.ons.gov.uk/peoplepopulationandcommunity/healthandsocialcare/conditionsanddiseases/datasets/cancerregistrationstatisticscancerregistrationstatisticsengland. Accessed September 2020].
